# Macrocyclic squaramides: anion receptors with high sulfate binding affinity and selectivity in aqueous media†

**DOI:** 10.1039/c6sc01011c

**Published:** 2016-04-01

**Authors:** Lei Qin, Anna Hartley, Peter Turner, Robert B. P. Elmes, Katrina A. Jolliffe

**Affiliations:** a School of Chemistry, The University of Sydney Sydney NSW 2006 Australia kate.jolliffe@sydney.edu.au +61 2 9351 3329 +61 2 9351 2297

## Abstract

A number of macrocyclic squaramide-containing receptors (MSQs) have been designed and synthesised and their interaction with a range of inorganic anions was studied in solution by ^1^H NMR spectroscopy and ESI-HRMS. The binding data revealed remarkable binding of sulfate in aqueous mixtures from 0.5 to 50% v/v H_2_O/DMSO-*d*_6_. The larger [3]-MSQs were found to better match the size and shape of the sulfate ion than the [2]-MSQs, providing high affinity and selectivity for sulfate while other tetrahedral divalent anions such as selenate, phosphate species and chromate have substantially lower binding affinities. In mixtures of anions mimicking the composition of either nuclear waste or plasma, the [3]-MSQs were still able to bind sulfate ions with high affinity.

## Abstract 2

Macrocyclic squaramide derivatives bind sulfate ions with high selectivity and affinity in aqueous mixtures.

## Graphical abstract

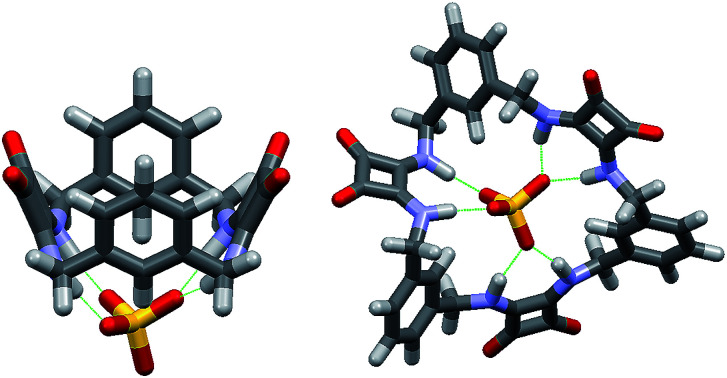

## Introduction

Inorganic sulfate plays numerous roles in environmental and biological systems, so the selective recognition of this anion has applications across a range of fields. For example, in industry the selective extraction of sulfate from nitrate rich mixtures in acidic (pH < 4) conditions would have significant benefits for nuclear waste remediation because sulfate interferes with the vitrification process that is a crucial procedure in nuclear waste treatment.^1–3^ In the context of biology, sulfate is essential for the formation of proteins in joints^4^ and mucin^5,6^ and low levels of sulfate have been found in the plasma of patients with rheumatoid arthritis^4^ and irritable bowel disease,^5,6^ so selective sulfate receptors could find applications in disease diagnosis and monitoring. However, binding of sulfate in aqueous solution is very challenging as a result of the high hydration energy of this anion (Δ*G* = −1090 kJ mol^−1^),^7^ which poses additional problems in terms of receptor selectivity in water since other anions, *e.g.* chloride and nitrate, have lower hydration energies (Δ*G* = −347 kJ mol^−1^ and −306 kJ mol^−1^, respectively).

Nature has overcome this challenge by employing an idealised arrangement of hydrogen bonding interactions in the sulfate binding protein (SBP)^8^ where three of the four sulfate oxygen atoms are bound through two hydrogen bonding interactions, while the fourth has a single hydrogen bond. These combined weak interactions result in a binding affinity for sulfate of 8.3 × 10^6^ M^−1^ across a pH range of 5–9^9^ and it has been hypothesised that this combination of weak interactions leads to fast exchange kinetics, allowing for error correction and therefore selectivity.^10^

Following Nature's lead, numerous receptors have been developed for the recognition and/or extraction of sulfate using hydrogen bonding interactions from ureas, thioureas, pyrroles, indoles, and squaramides,^11–24^ but very few of these show significant sulfate affinity in aqueous solution. To overcome this, charged interactions are often incorporated into sulfate receptors to provide increased binding energies to compensate for the dehydration ‘penalty’. Even so, few receptors are capable of binding to sulfate in aqueous solution^25–29^ and the introduction of charged interactions frequently occurs at the cost of selectivity or functionality (*e.g.* cationic receptors are likely to be toxic^30^ and so unable to be used in biological applications). The development of neutral receptors capable of binding selectively to sulfate in aqueous solution is therefore of particular interest.

**Fig. 1:**
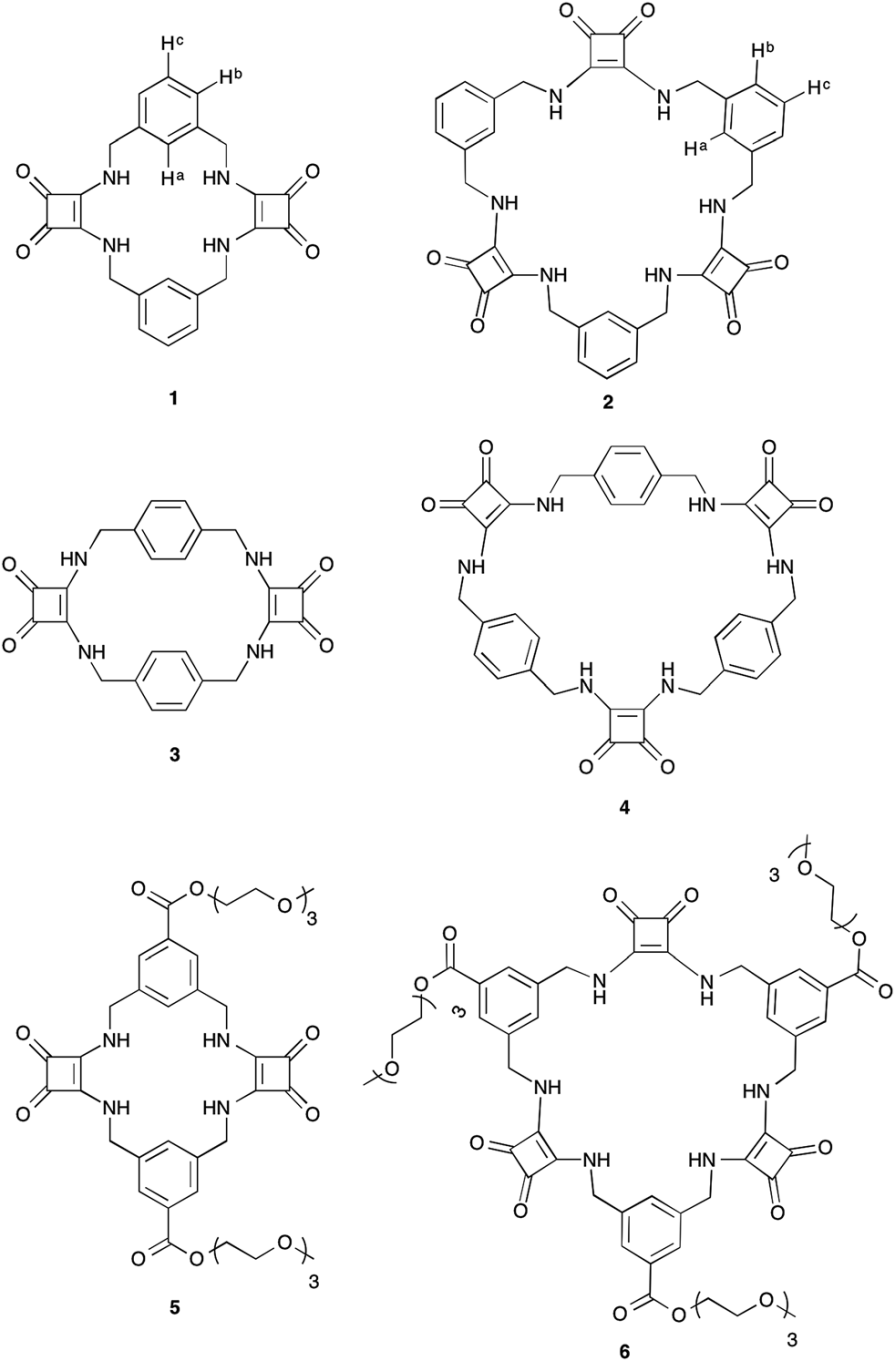
Macrocyclic squaramides (MSQs) 1–6.

It is well established that to achieve strong and selective binding, receptors should be preorganised to match the size and shape of the target ion. Macrocyclic receptors have therefore found great utility in the selective recognition of a range of cations and anions.^31^ We report herein the first examples of a new class of macrocyclic anion receptors 1–4 (Fig. 1) containing alternating aryl spacers and squaramides spaced by methylene units, together with the more soluble triethylene glycol monomethyl ether derivatives 5 and 6 (Fig. 1).

## Results and discussion

### Synthesis and characterisation of MSQs 1–4

While Costa and co-workers have previously demonstrated the self-templated formation of macrocycles containing both squaramides and a hydrogen-bond acceptor unit,^32,33^ attempts to prepare a macrocycle containing two squaramide units linked by alkyl chains (*i.e.* lacking the hydrogen bond acceptor) have been reported to be unsuccessful, giving only traces of the desired compound. We have now developed a simple yet versatile synthetic route to macrocycles comprising alternating squaramide and benzylic groups (macrocyclic squaramides: MSQs), enabling us to quickly assemble the desired frameworks containing either two or three squaramide units and starting from the readily available diethyl squarate and appropriate benzyl diamines. We envisaged that this would allow the development of analogues that could be readily functionalised by substitution of the aromatic rings to tailor the solubility of the macrocycles for a variety of applications.

**Scheme 1:**
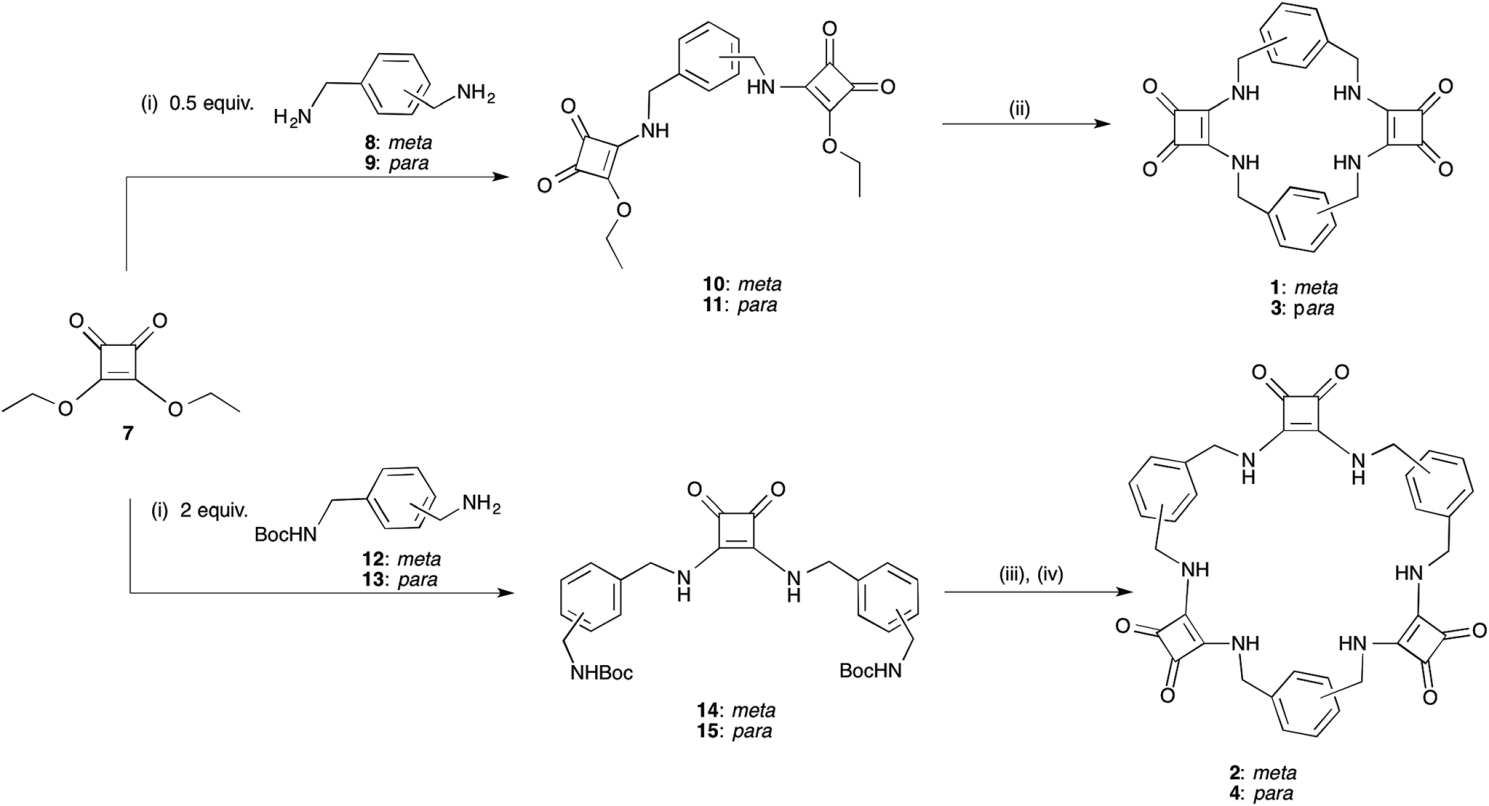
Synthesis of the desired macrocyclic squaramide based receptor frameworks 1–4. Synthesis: (i) Et_3_N, EtOH; (ii) 1 equiv. 8 or 9, high dilution, Et_3_N, EtOH, heat. (iii) TFA/DCM (1 : 1); (iv) 1 equiv. 10 or 11, high dilution, Et_3_N, EtOH, heat.

[2]-MSQs 1 and 3 were synthesised in a two-step process (Scheme 1), exploiting the ability of diethylsquarate 7 to undergo sequential amidation reactions.^34^ Treatment of an excess of diethylsquarate with one equivalent of the appropriately substituted benzyl diamines 8 and 9 gave the corresponding disquarate derivatives 10 and 11. Subsequent reaction of 10 and 11 with the benzyl diamines 8 and 9 in the presence of Et_3_N under high dilution conditions yielded macrocycles 1 and 2 in 87% and 93% yield, respectively. Similarly, the larger [3]-MSQs (2 and 4) were assembled (Scheme 1), by initial reaction of two equivalents of the known mono Boc-protected benzyl diamines 12 and 13^35^ with 7 in the presence of Et_3_N to yield the corresponding dibenzyl squarate derivatives 14 and 15. Following Boc deprotection in the presence of trifluoroacetic acid (TFA), reaction of the subsequently formed diamines with the aforementioned disquarates 10 and 11 in the presence of Et_3_N under high dilution conditions gave 2 and 4 in 79% and 68% yield, respectively.

**Fig. 2:**
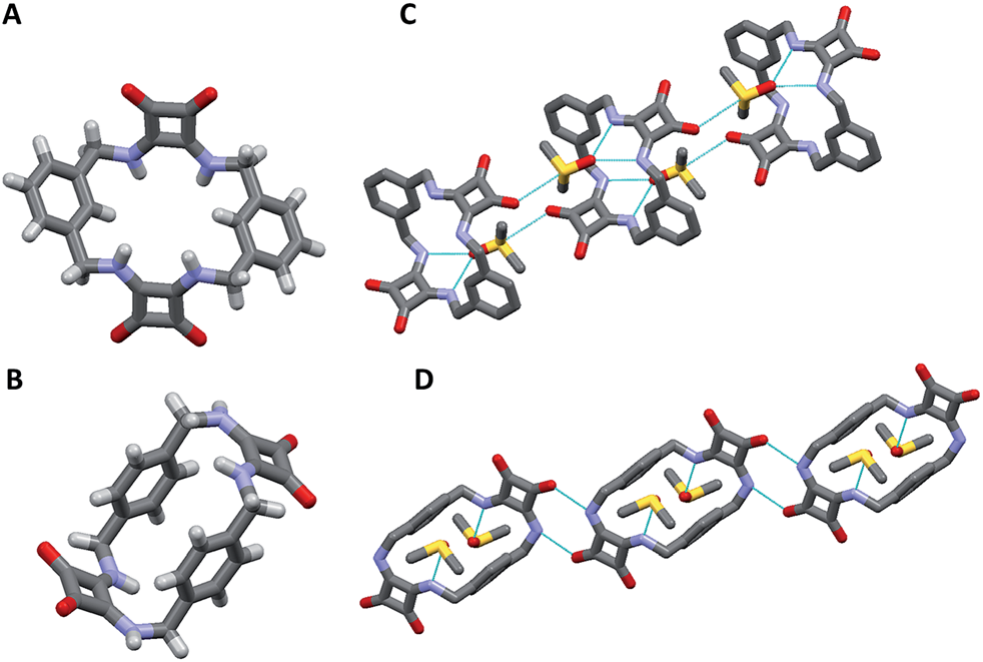
Single crystal X-ray diffraction structures of (A) 1, (B) 3 and the solid state intermolecular hydrogen bond interactions of (C) 1 and (D) 3 in the presence of DMSO; hydrogen atoms not shown.

[2]-MSQs 1 and 3 were both successfully recrystallised from concentrated solutions of DMSO to yield samples suitable for single crystal X-ray diffraction analysis (Fig. 2), which confirmed the structure of the anticipated macrocyclic [2]-MSQs 1 and 3. Macrocycles 1 and 3 were each found to be hydrogen-bonded to a molecule of DMSO in the solid state with the squaramide moiety acting as both a H-bond donor and acceptor to a pair of DMSO molecules as previously observed for acyclic squaramide derivatives.^36,37^ As expected, though both are centred on a crystallographic inversion site with squaramide moieties on opposite sides of the macrocycle cavity plane, the substitution pattern around the aryl rings of 1 and 3 gave macrocycles with substantially different conformations. Macrocycle 1 was found to adopt a ‘chair-like’ conformation that maintains the *cis*/*cis* conformation around each squaramide that is optimal for anion complexation. In contrast, 3 has a *cis*/*trans* arrangement of the squaramide NH protons (Fig. 2B) and complementary hydrogen bond interactions between the squaramide groups of adjacent molecules that link them in a chain like manner in the solid state (Fig. 2D). The length and width of the macrocycle cavity is effectively determined by the separation between the four ‘corner’ carbon atoms that link the phenyl groups and squaramide groups and in 1 these distances are 6.062(2) Å across the squaramide group (distance between C5 and C12; see the ESI†) and 5.075(2) Å across the phenyl group (distance between C5 and C12). In 3 these distances are effectively ‘reversed’ and are respectively 4.732(3) and 5.837(2) Å. Notwithstanding this difference, in both cases the cavity dimensions are promising for anion recognition.

The cavity shape in 1 is essentially rectangular, with internal corner angles of approximately 87.1° and 92.9°, while that of 3 is that of a parallelogram with internal corner angles of 82.6° and 97.4°. In 1 the least squares planes of the squaramide groups are separated by 4.832(6) Å, while in 3 the distance is 6.288(6) Å. The centroid to centroid distance and the approximate relative lateral offset of the squaramide groups is respectively 7.233(2) and 5.38 Å in 1, and 8.590(2) and 5.85 Å in 3. The least squares planes of the phenyl groups in 1 are separated by 3.672(5) Å and in 3 the separation is 4.635(2) Å. The centroid to centroid distance and the approximate relative lateral offset of the phenyl groups is approximately 8.463(2) and 7.62 Å in 1, in contrast to 4.667(2) and 0.55 Å in 3. Additional cavity geometry details are provided in the ESI.† Unfortunately, single crystals suitable for X-ray crystallographic analysis could not be obtained for the larger [3]-MSQs 2 and 4.

^1^H NMR spectroscopy provided further information about the conformations of the constrained macrocycles 1–4 in the solution phase. In DMSO-*d*_6_, the *meta*-substituted compounds 1 and 3 exhibited broad signals in the ^1^H NMR spectra, suggesting that these compounds exist in multiple conformations which interconvert on a similar timescale to the NMR experiment. In the case of the *para*-substituted compounds 2 and 4, complex NMR spectra were obtained with multiple signals observed for each different type of proton, indicating that different conformers of these macrocycles exist in solution and that interconversion between these is slow on the NMR timescale. Similarly, compounds 2 and 4 also gave ^13^C NMR spectra with broadened and multiple signals for the same type of carbon, while the signals in the ^13^C NMR spectra of compounds 1 and 3 were slightly broadened.§§Variable temperature ^1^H NMR experiments were attempted but these did not provide further information on the rate of interconversion of the conformers, partly as a result of the low solubility of these compounds in DMSO.

### Anion binding studies of MSQs 1–4

With MSQs 1–4 in hand, qualitative screening experiments were undertaken using ^1^H NMR spectroscopy to identify the optimal anionic guests for these macrocycles. For receptors 1 and 2, anion screening experiments were performed by addition of 10 equiv. of a range of common anions (F^−^, Cl^−^, Br^−^, NO_3_^−^, HSO_4_^−^, AcO^−^, H_2_PO_4_^−^ and SO_4_^2−^ as their tetrabutylammonium salts) to 1 and 2 in DMSO-*d*_6_. These preliminary results indicated significant changes in the ^1^H NMR spectra of 1 and 2 in the presence of H_2_PO_4_^−^, AcO^−^ and SO_4_^2−^ that culminated in downfield shifts of the squaramide NH proton signals (*e.g.* Δ*δ* = 2.21 for 1 with SO_4_^2−^, Δ*δ* = 1.61 for 2 with SO_4_^2−^) and a significant downfield shift of the aromatic proton lying within the macrocyclic cavity in the case of SO_4_^2−^ addition, (CH) (*e.g.* Δ*δ* = 0.87 for 1, Δ*δ* = 0.44 for 2), which was less pronounced in the presence of other anions. Conversely, less pronounced changes were observed in the presence of Cl^−^, Br^−^, HSO_4_^−^ and NO_3_^−^ suggesting weaker interactions of these anions with 1 and 2. In the presence of F^−^ the signals for the NH protons could not be observed, suggesting likely deprotonation of the squaramides by this basic anion, as has previously been observed for simple squaramide derivatives.^37^ Unfortunately, the complex ^1^H NMR spectra and low solubility of the *para*-substituted derivatives 3 and 4 did not allow the ready screening of their anion binding ability. However, for both 3 and 4, the addition of one equivalent of either AcO^−^ or SO_4_^2−^ resulted in significant changes and simplification of the NMR spectra. Complete dissolution of suspensions of 3 and 4 in DMSO-*d*_6_ was observed upon addition of 1.2 equiv. of sulfate and this together with the simplified NMR spectra provides evidence that these compounds bind strongly to this anion.

Given the low solubility and complex ^1^H NMR spectra of 3 and 4, quantitative anion binding studies were only performed for the *meta*-substituted MSQs 1 and 2. ^1^H NMR titrations of 1 and 2 in DMSO-*d*_6_ containing 0.5% water were performed by addition of the tetrabutylammonium salts of SO_4_^2−^, H_2_PO_4_^−^, AcO^−^ and Cl^−^ (and HSO_4_^−^ for 1) with the resulting data fit to a 1 : 1 binding model using a global fitting method in Hyperquad^38^ (with the exception of binding of 1 to HSO_4_^−^; the data for which could not be fit to a suitable binding model) to provide the apparent stability constants (*K*_a_), which are summarised in Table 1. Both 1 and 2 were found to bind to SO_4_^2−^ with affinities too high to measure accurately (>10^4^ M^−1^) using NMR but significant differences in binding affinities for the other anions were observed, with [3]-MSQ 2 showing excellent selectivity for SO_4_^2−^, whereas 1 also bound strongly to H_2_PO_4_^−^ and AcO^−^ ions.

**Table 1:** Apparent stability constants (*K*_a_) of MSQs 1 and 2^a^

MSQs	SO_4_^2−^/M^−1^	H_2_PO_4_^−^/M^−1^	AcO^−^/M^−1^	Cl^−^/M^−1^
1	>10^4^	>10^4^	7530	28
2	>10^4^	409	776	144

a Titrations were performed in DMSO-*d*_6_ with 0.5% water at 300 K. Estimated errors < 15%.

**Fig. 3:**
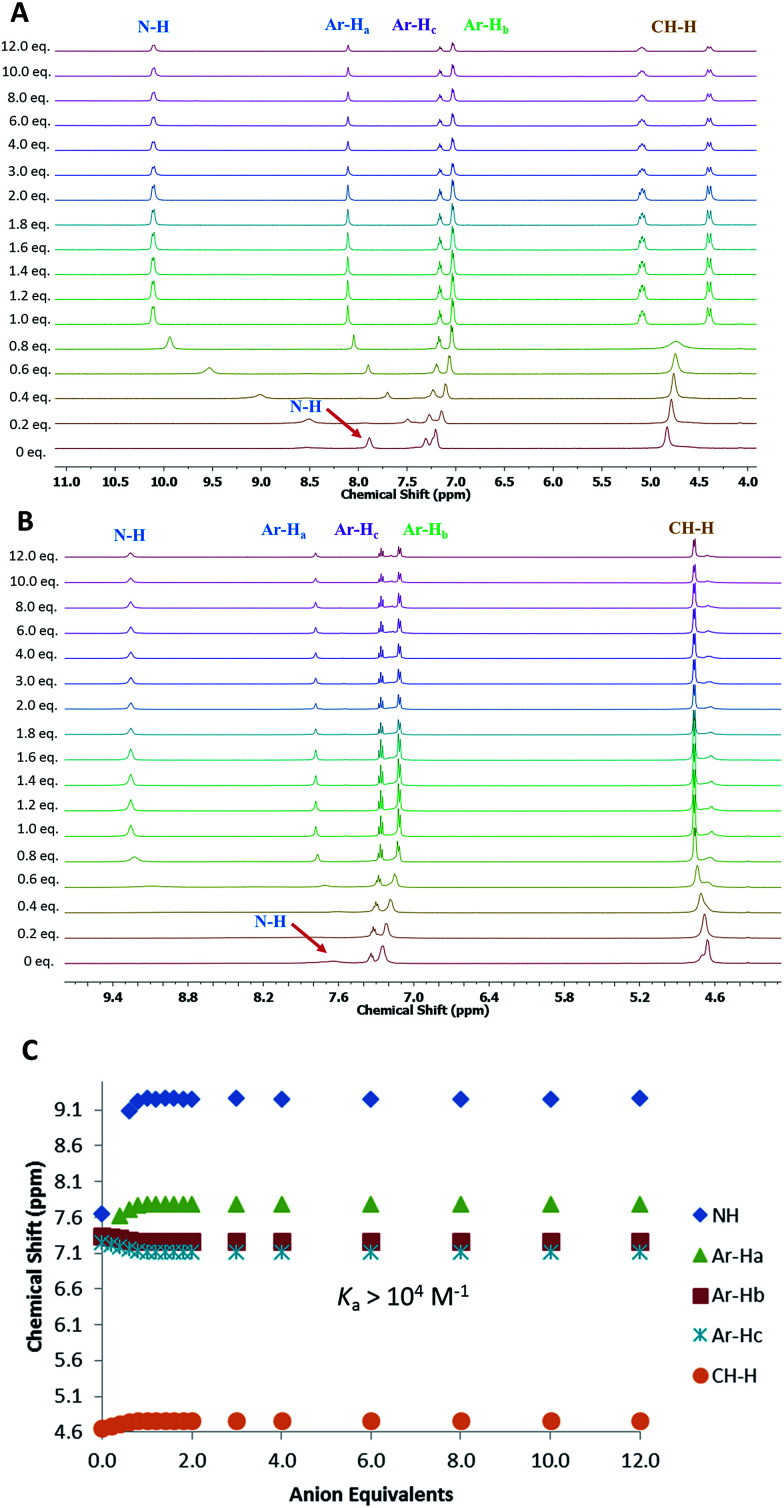
(A): ^1^H NMR titration of compound 1 with (TBA)_2_SO_4_ in DMSO-d_6_ with 0.5% water at 300 K. (B): ^1^H NMR titration of compound 2 with (TBA)_2_SO_4_ in DMSO-d_6_ with 0.5% water at 300 K. (C): Comparison isotherms of squaramide NH, aromatic protons (Ar-H_a_, H_b_, H_c_) for 2 in the presence of increasing concentrations of SO_4_^2−^.

Analysis of the ^1^H NMR spectra of the complexes also provided useful information on the binding modes of the MSQs for the different anions. For [2]-MSQ 1, addition of either Cl^−^ or AcO^−^ resulted in downfield shifts of the signals attributable to the NH protons, but the signals attributable to the aromatic protons and the methylene bridges did not change or shift significantly, although they did become slightly sharper. In contrast, addition of H_2_PO_4_^−^ and SO_4_^2−^ to 1 resulted in significant downfield shifts of both the NH proton signals and the signal attributable to the aromatic CH that sits between the two ring substituents, with the other aromatic proton signals exhibiting slight upfield shifts (Fig. 3). In addition the signal for the benzylic protons (CH_2_) broadened in the case of H_2_PO_4_^−^ addition, while upon addition of SO_4_^2−^ this signal shifted slightly upfield and broadened until one equivalent of sulfate was added, at which point it sharpened and split into two distinct signals indicating the inequivalence of the two protons on each CH_2_ group and suggesting that, upon full complexation to the sulfate ion, the macrocycle is frozen into a single conformer. The multiplicity of these signals indicates an AB quartet, in which one of the signals is further split by coupling to the adjacent NH proton while the other is not, as a result of the different dihedral angles between the two CH_2_ protons and the NH proton. For [3]-MSQ 2, a similar trend was observed, with addition of Cl^−^ and AcO^−^ resulting in significant downfield shifts only of the squaramide NH proton signal, whereas addition of both H_2_PO_4_^−^ and SO_4_^2−^ resulted in significant shifts for both the NH protons (downfield) and the aromatic protons (one signal moves downfield while the others shift upfield). In this case the signal for the CH_2_ protons sharpens upon addition of the anions and no splitting of the signal is observed, even upon addition of excess sulfate ion.

Further information about the interaction of MSQs 1–3 with SO_4_^2−^ and AcO^−^ was ascertained from ESI mass spectrometric analysis of 0.5 mM solutions of 1–3 in MeCN with either (TBA)_2_SO_4_ or TBAAcO. In the case of AcO^−^ addition, mass spectra of 1 and 2 exhibited major peaks at *m*/*z* 1213.006 and 1608.336 that were assigned to the doubly deprotonated [1 − 2H^+^ + 3(TBA^+^)]^+^ and triply deprotonated receptors [2 − 3H^+^ + 4(TBA^+^)]^+^, respectively. Very minor peaks were observed for the [M + OAc^−^ + 2(TBA^+^)]^+^ ion thus suggesting that the complex formed between AcO^−^ and the MSQs is not stable enough to be observed using ESI mass spectrometric analysis. Conversely, in the case of addition of TBA_2_(SO_4_) to 1 and 2, the major peaks observed had an *m*/*z* ratio and isotopic pattern that can be assigned to the 1 : 1 SO_4_^2−^ complexes. Major peaks at *m*/*z* = 1250.954 and 1465.026 corresponded to the [M + SO_4_^2−^ + 3(TBA^+^)]^+^ complexes of 1 and 2, respectively. No peaks were found correlating to either deprotonated receptors or to ions with more than one SO_4_^2−^ anion further suggesting that SO_4_^2−^ binds to 1 and 2 in a 1 : 1 fashion. These observations were further supported by NMR Job plot analysis (see ESI†), which also indicated a 1 : 1 binding mode between SO_4_^2−^ and these receptors. Conversely, the mass spectrum of a mixture of 3 and (TBA)_2_SO_4_ displayed significant peaks at both *m*/*z* 1250.953 and *m*/*z* 1590.196 correlating to the masses of the 1 : 1 [M + SO_4_^2−^ + 3(TBA^+^)]^+^ and 2 : 1 [M + H^+^ + 2SO_4_^2−^ + 4(TBA^+^)]^+^ complexes, respectively suggesting both binding stoichiometries are possible for complexes of SO_4_^2−^ and 3.

**Fig. 4:**
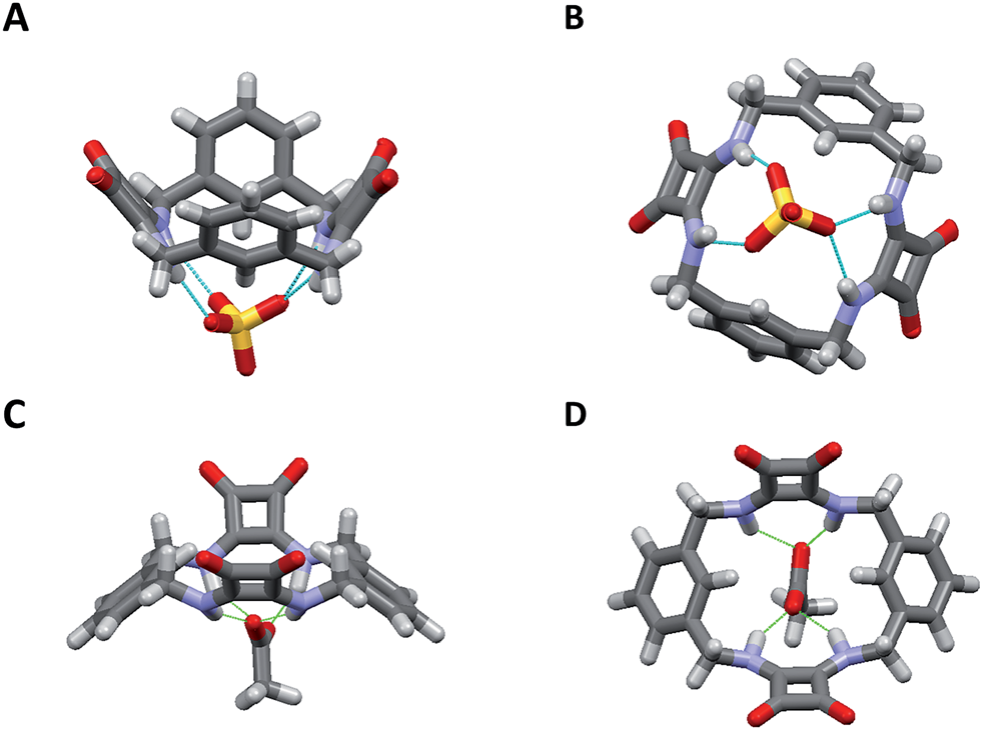
(A) The dominant binding mode of the complex formed between 1 and SO_4_^2−^ viewed from the side and (B) from below clearly showing the interaction of SO_4_^2−^ with the squaramide NH protons inside the macrocyclic cavity. The structure was obtained by single crystal X-ray diffraction. (C) Energy minimised molecular model of the complex formed between 1 and AcO^−^ viewed from the side and (D) from above.

Final confirmation of the binding modes between 1 and AcO^−^ and SO_4_^2−^ were identified by a combination of both X-ray crystallographic analysis and molecular modeling (Fig. 4). Crystals of 1 suitable for single crystal X-ray diffraction analysis were obtained in the presence of 5 equiv. of (TBA)_2_SO_4_ by slow evaporation of an MeCN solution. Analysis revealed that in the presence of SO_4_^2−^ the conformation adopted by 1 is markedly different to the *S*_2_ symmetry chair-like conformation found for the DMSO complex of 1. Instead, 1 forms a 1 : 1 (receptor/anion) complex with SO_4_^2−^ where the receptor adopts a bowl-like conformation with essentially *C*_2v_ symmetry and suggestive of a calixarene in the cone conformation (Fig. 4). The two least squares planes defined by the squaramide carbon atoms are inclined with respect to each other by 73.5(1)° and the two least squares planes of the phenyl groups form an angle of 50.78(8)°. The squaramides are inclined at 57.39(9) and 49.12(8)° with respect to the least squares plane of the cavity, while the phenyl group planes are inclined at 52.68(6) and 76.55(6)° with respect to the cavity plane (see the ESI†). Both squaramide functionalities and two aromatic CH protons (one from each ring) are directed into the macrocyclic cavity. In this conformation, the two protons at each benzylic position are clearly in different environments (pseudo-axial and pseudo-equatorial), corroborating the results observed in the ^1^H NMR titration experiments.

The macrocycle cavity dimensions of the 1·SO_4_^2−^ complex are very similar to those of 1, with ‘corner’ carbon to corner carbon distances across the squaramide groups of 6.070(3) and 6.041(2) Å and corner to corner distances across the phenyl groups of 5.050(4), 4.998(3) Å. The internal angles are approximately 89.1, 90.6, 89.9 and 90.6°. The squaramide centroid to squaramide centroid and phenyl centroid to phenyl centroid distances are respectively 7.152(3) and 7.323(2) Å. The crystals structure reveals two binding modes (see Fig. S34 in the ESI material†), with one having hydrogen bonds between each of the four amide protons and three of the sulfate oxygens, while the other mode involves just two of the sulfate oxygens. The binding mode involving three oxygen atoms is the dominant mode (see the ESI†) and is shown in Fig. 4 (N⋯O distances from 2.745(2) to 2.881(2) Å; details are provided in the ESI†). The anionic macrocycle-sulfate complex is ‘nestled’ between the pendant residues of a pair of tetrabutylammonium cations (see Fig. S35†).

While crystals suitable for X-ray crystallography could not be obtained for the complex of 1 with AcO^−^, molecular modelling was performed using Spartan 14 (Wavefunction Inc.) to provide an indication of the binding mode of 1 with AcO^−^. The structure of 1 was energy minimized using molecular mechanics then an AcO^−^ ion was placed into the centre of macrocyclic cavity of 1 and the resulting complex was optimised by density functional theory (DFT) calculations at the B3LYP/6-31G* level of theory. As shown in Fig. 4, these calculations indicate that 1 adopts an alternative conformation when binding the AcO^−^ ion. The encapsulated anion is again bound by four H-bonding interactions with both available squaramide functionalities, however, in this case the aromatic CH proton is clearly pointing away from the anion binding site, in a conformation reminiscent of a 1,3-alternate calixarene conformation. This observation suggests an alternative mode for AcO^−^ binding and corroborates the results observed in the NMR measurements, accounting for the shifts of the NH protons upon AcO^−^ binding with the concomitant lack of shifts of the aromatic CH protons that were observed for binding of SO_4_^2−^. When the sulfate complex of 1 was modelled in a similar manner (see Fig. S31 in the ESI material†), the resulting structure was very similar to that observed by X-ray crystallography.

**Fig. 5:**
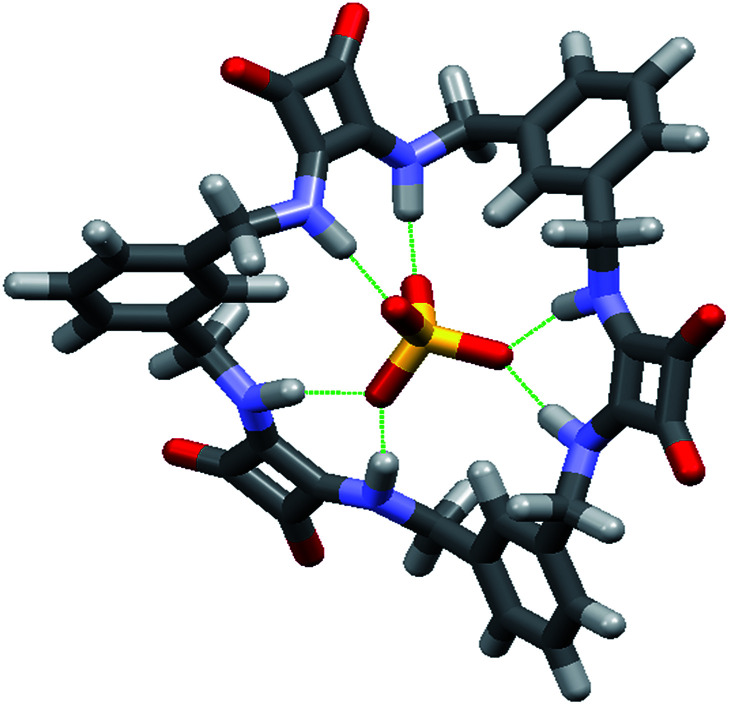
Energy minimised molecular model of the complex formed between 2 and sulfate.

In the absence of X-ray quality crystals, the complex between the [3]-MSQ 2 and sulfate was also modelled to give an indication of the binding mode (Fig. 5). The modelled structure shows that 2 can coordinate to sulfate through six N–H hydrogen bonds, with all four of the sulfate oxygen atoms involved (in contrast to the complex observed for 1) where two of the squaramide units each have two hydrogen bonds to a single oxygen atom, while the third squaramide unit forms one hydrogen bond from each of its NH protons to two of the oxygen atoms. The three aromatic rings all have a single proton projecting towards the sulfate ion, explaining the downfield shift observed for the signal attributable to these protons in the ^1^H NMR experiments, and suggesting that in addition to the six N–H hydrogen bonding interactions, an additional three C–H bonding interactions^39,40^ from the receptor to the anion may be contributing to the high binding affinity observed for this complex.

### Synthesis and characterisation of MSQs 5 and 6

MSQs 1 and 2 exhibited high affinity and, in the case of 2, excellent selectivity for sulfate in DMSO (0.5% H_2_O) but investigation of their binding affinity in solvents other than DMSO was hampered by the low solubility of these compounds. In order to overcome this limitation we designed and synthesised the triethylene glycol monomethyl ether-macrocyclic squaramide based receptors “TEG-MSQs” 5–6 (Fig. 1). We anticipated that the incorporation of the TEG esters would significantly improve the solubility of the MSQs in DMSO as well as providing increased water solubility.

**Scheme 2:**
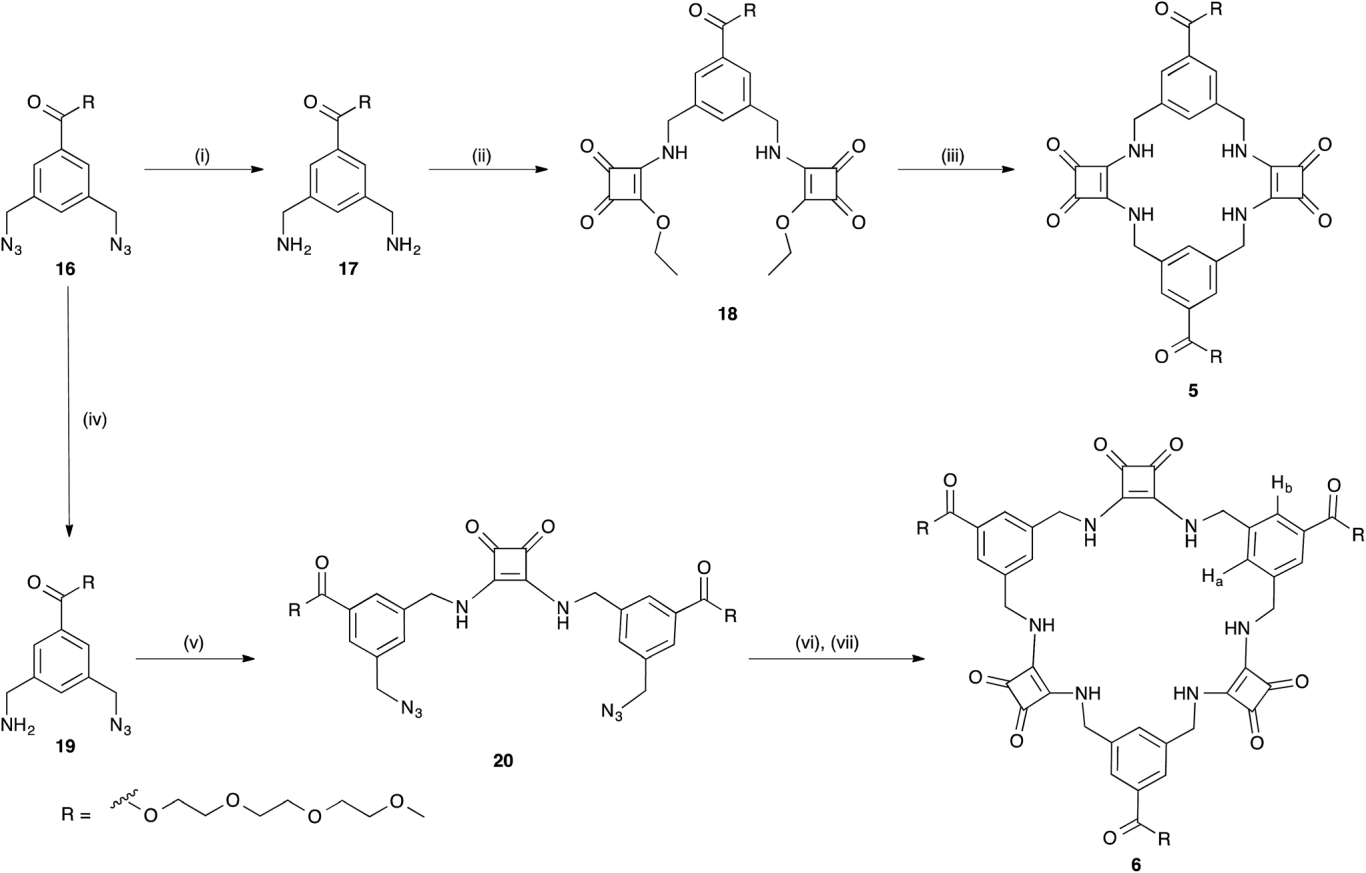
Synthesis of the desired macrocyclic squaramide based receptor frameworks 5 and 6. Synthesis: (i) Ph_3_P (2 equiv.), THF/H_2_O, RT, 8 h, 71%; (ii) 7 (2 equiv.), EtOH, Et_3_N, overnight, RT, 80%; (iii) 17 (1 equiv.) EtOH, Et_3_N, overnight, RT, 32%; (iv) Ph_3_P (1 equiv.), THF/H_2_O, RT, 8 h, 52%; (v) 7 (0.5 equiv.) EtOH, Et_3_N, overnight, RT, 77%; (vi) Ph_3_P, THF/H_2_O, RT, 8 h; (vii) 18 (1 equiv.) EtOH, Et_3_N, overnight, RT, 35%.

**Fig. 6:**
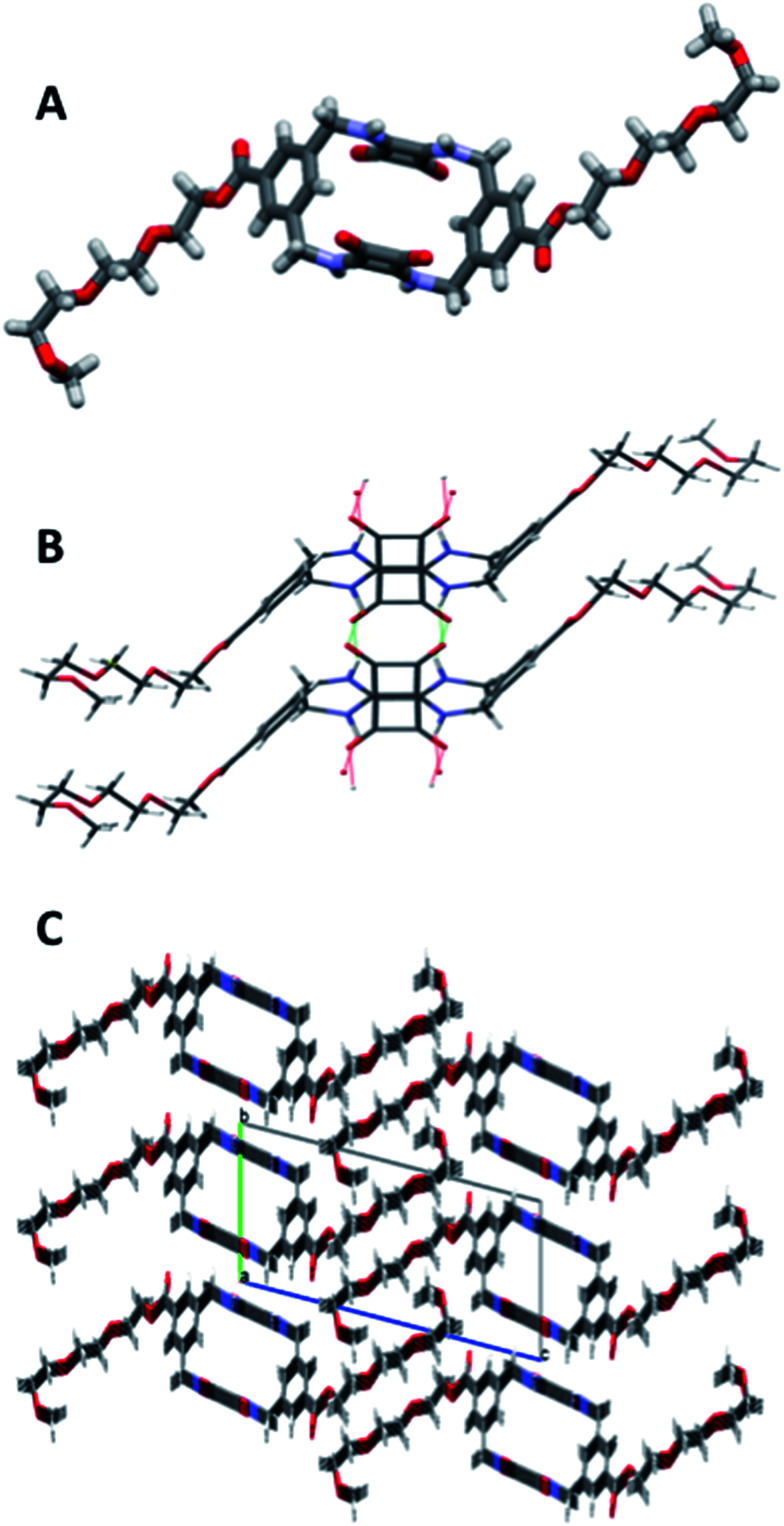
X-ray crystallography determined structure of 5 (A) side view; (B) hydrogen bonds link adjacent molecules to form an approximately rectangular channel parallel to the *a* axis and centred on (0, 1/2, 0); (C) view down the *a* axis of the unit cell showing the pseudo-rectangular channels arising from hydrogen bonds between adjacent squaramides. Disorder at the end of the pendant TEG chains is not shown.

In order to prepare 5 and 6*via* our previously established synthetic route, we required the TEG-functionalised diamine 17. The reduction of the known bis(azidomethyl)benzene derivative 16^41^ (Scheme 2) was performed *via* Staudinger reaction conditions using triphenylphosphine (Ph_3_P) in THF/water to provide the benzyl diamine 17 in 71% yield. Compound 17 was observed to be prone to rapid decomposition at room temperature. However, rapid purification of 17 followed by immediate reaction with diethyl squarate 7 in ethanol afforded compound 18 in 80% yield. Subsequent reaction of compound 18 with a further portion of the benzyl diamine 17 in ethanol and trimethylamine (Et_3_N) afforded the desired macrocycle TEG-[2]-MSQ 5 in 32% yield (Scheme 2). The lower yield for this reaction as compared to that obtained for the analogous synthesis of 1 is likely a result of decomposition of the unstable 17 under the reaction conditions. Recrystallization of compound 5 from a concentrated solution in DMSO/H_2_O (approx. 8 : 2 v/v) afforded crystals suitable for single crystal X-ray diffraction analysis (Fig. 6), providing confirmation of the structure of the anticipated macrocyclic structure 5. The crystal structure reveals that 5 adopts a similar *S*_2_ symmetry ‘chair-like’ conformation to that of 1 in the absence of an anionic guest. In contrast to 1 however, DMSO has not been ‘captured’ and incorporated into the solid state. Instead hydrogen bonds between the squaramide groups of adjacent molecules link them together such that their aligned macrocycles form an approximately rectangular channel (Fig. 6C and S37 and S38 in the ESI material†) in the solid state. The cavity dimensions are very similar to those of 1 and 1 + SO_4_^2−^, with the corner carbon to corner carbon distance across the squaramide group being 6.125(4) Å and that across the phenyl group being 5.031(4) Å. The internal corner angles are approximately 90.1, and 89.9°. The least squares planes of the squaramide and phenyl groups are respectively inclined at angles of 77.9(1) and 53.23(7)° with respect to that of the macrocycle cavity. Related by inversion symmetry, the two planes defined by the squaramide groups are parallel to each other and separated by a distance of 4.891(4) Å; this constitutes the smallest dimension of the channel formed by the alignment of the macrocycles. The squaramides of a macrocycle have an approximate relative lateral offset of approximately 1.70 Å and a centroid to centroid distance of 5.179(3) Å. The phenyl centroid to centroid distance is 8.308(4) Å and they have a relative offset of approximately 6.58 Å. Additional details are provided in the ESI,† which includes a tabulated comparison of aspects of the cavity geometry of 1, 3, 1 + SO_4_^2−^ and 5.

Initial attempts to prepare 6 using a similar synthetic route to that employed to prepare 2 were unsuccessful, presumably as a result of the instability of diamine 17. We therefore employed a slightly modified synthetic route to obtain 23. This began with the reduction of diazide 16 using only one equivalent of triphenylphosphine (Ph_3_P) in THF/water to afford the monoamine 19 in 52% yield. The reaction of compound 19 with diethyl squarate 7 and triethylamine in ethanol afforded bis-azide substituted squaramide 20 in 77% yield. A single pot Staudinger reduction of 20 and subsequent reaction with compound 18, synthesised previously, in the presence of triethylamine and ethanol afforded the desired TEG-[3]-MSQ 6 in 35% over the two steps. Gratifyingly, the incorporation of the TEG esters provided significantly increased solubility for both 5 and 6 as compared to the parent macrocycles 1 and 2. 5 and 6 were found to be soluble in mixtures of up to 33% water in DMSO, enabling anion binding experiments to be performed in this more competitive solvent mixture.

### Binding studies of TEG-MSQs 5 and 6

**Fig. 7:**
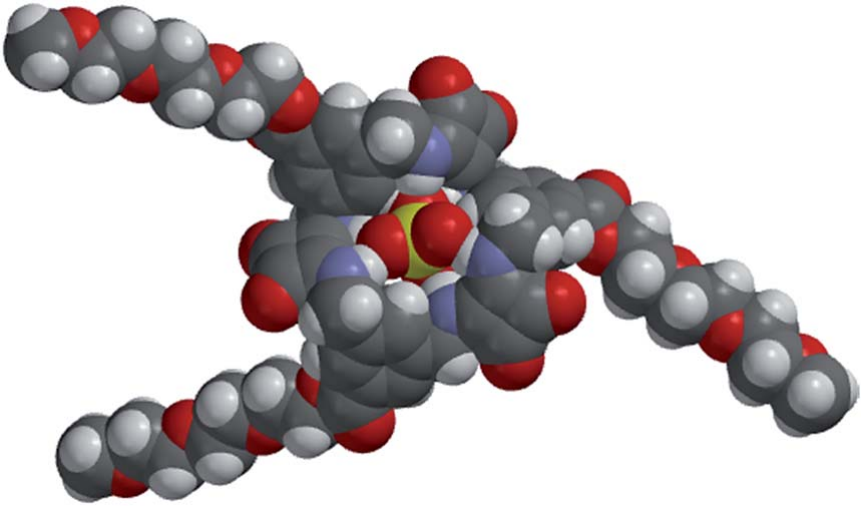
Calculated space filling model of [TEG-MSQ 6·SO_4_]^2−^ complex.

Given that MSQs 1–4 were found to bind strongly to SO_4_^2−^ (and H_2_PO_4_^−^ in the case of 1) we focused on determining the ability of 5 and 6 to distinguish between tetrahedral and divalent anions, since even the natural sulfate-binding protein (SBP) is unable to provide high levels of discrimination between its target sulfate ion (SO_4_^2−^*K*_a_ = 8.33 × 10^6^ M^−1^) and ions such as SeO_4_^2−^ (*K*_a_ = 0.2 × 10^6^ M^−1^) or CrO_4_^2−^ (*K*_a_ = 3.33 × 10^6^ M^−1^) at neutral pH.^9^ Hence, quantitative NMR binding studies of TEG-MSQs 5–6 for a range of anions were conducted in 1 : 2 (v/v) H_2_O/DMSO-*d*_6_ using WATERGATE (see ESI for details†) to suppress the H_2_O signal. Binding affinities calculated by fitting to a 1 : 1 binding model are given in Table 2. Compared with the binding observed for MSQs 1 and 2 to sulfate (*K*_a_ > 10^4^ M^−1^) in DMSO-*d*_6_, in the more competitive solvent mixture [1 : 2 (v/v) H_2_O/DMSO-*d*_6_], TEG-[2]-MSQ 5 showed a dramatic decrease in affinity for sulfate (*K*_a_ = 1819 M^−1^). However, TEG-[3]-MSQ 6 still exhibited affinity for SO_4_^2−^ that was too high to quantify (*K*_a_ > 10^4^ M^−1^) in these very competitive conditions. We speculate that the high binding affinity of 6 for sulfate is not only a result of the [3]-MSQs having an additional squaramide binding site compared to 5, together with additional C–H hydrogen bonding interactions to the anion, but primarily because the cavity size of the [3]-MSQ is well-matched to the size of the sulfate ion resulting in the anion sitting inside the cavity of the TEG-[3]-MSQ 6 in a manner that protects it from solvent molecules, as indicated by the molecular model depicted in Fig. 7. In contrast, the crystal structure of the [MSQ 1·SO_4_]^2−^ complex (Fig. 4A) shows that, in this smaller receptor, the sulfate is perched on top of the macrocyclic cavity and still exposed to solvent molecules.

**Table 2:** Association constants (*K*_a_) of receptors 5 and 6 for anions^a^

Anion	5_1 : 2 v/v H_2_O/DMSO	6_1 : 2 v/v H_2_O/DMSO
SO_4_^2−^/M^−1^	1820	>10^4^
H_2_PO_4_^−^/M^−1^	57	16
SeO_4_^2−^/M^−1^	166	1900
Cr_2_O_7_^2−^/M^−1^	17	—b
CrO_4_^2−^/M^−1^	96	165
HCO_3_^−^/M^−1^	—b	—b
NO_3_^−^/M^−1^	—b	—b
AcO^−^/M^−1^	—b	—b
BF_4_^−^/M^−1^	—b	—b
ClO_4_^−^/M^−1^	—b	—b
*p*-Toluenesulfonate/M^−1^	—b	—b
ReO_4_^−^/M^−1^	—b	—b

a Titrations were performed in 1 : 2 (v/v) H_2_O/DMSO-d_6_ with at 300 K. Estimated errors < 15%.

b Binding too weak to obtain an accurate binding constant.

[3]TEG-MSQ 6 also reveals remarkable selectivity for sulfate amongst other tetrahedral anions (Table 2), with binding affinity for sulfate at least three orders of magnitude stronger than that observed for phosphate species (a mix of H_2_PO_4_^−^ and HPO_4_^2−^ at neutral pH) and perchlorate (ClO_4_^−^). Furthermore, TEG-[3]-MSQ 6 binds to sulfate with significantly higher affinity than it does to selenate or chromate ions, respectively. While selectivity of 6 for sulfate over selenate (*K*_a(sulfate)_/*K*_a(selenate)_ = >5)¶¶Because *K*_a(sulfate)_ for 6 is >10^4^ M^−1^, the minimum value was chosen to calculate the selectivity ratio. is likely to be lower than that of the SBP (*K*_a(sulfate)_/*K*_a(selenate)_ = 41), the ability of 6 to discriminate between sulfate and chromate (*K*_a(sulfate)_/*K*_a(chromate)_ = >60) is significantly higher than that of the SBP (*K*_a(sulfate)_/*K*_a(chromate)_ = 2.5). In order to better assess the ability of both 5 and 6 to discriminate between the doubly charged SO_4_^2−^ and HPO_4_^2−^ ions we also performed titrations with these ions in 2 : 1 DMSO-*d*_6_/aq. Tris buffer (15 mM) at pH 9.1. Under these conditions, for [3]-MSQ 6*K*_a_ values of 2045 M^−1^ and 13 M^−1^ were obtained for sulfate and hydrogenphosphate, respectively giving a selectivity of *K*_a(sulfate)_/*K*_a(hydrogenphosphate)_ = 157.||||In 2 : 1 DMSO-*d*_6_/aq. Tris buffer (15 mM) at pH 9.1, [2]-MSQ gave *K*_a_ = 235 M^−1^ for SO_4_^2−^ and *K*_a_ < 10 M^−1^ for HPO_4_^2−^, respectively.

**Fig. 8:**
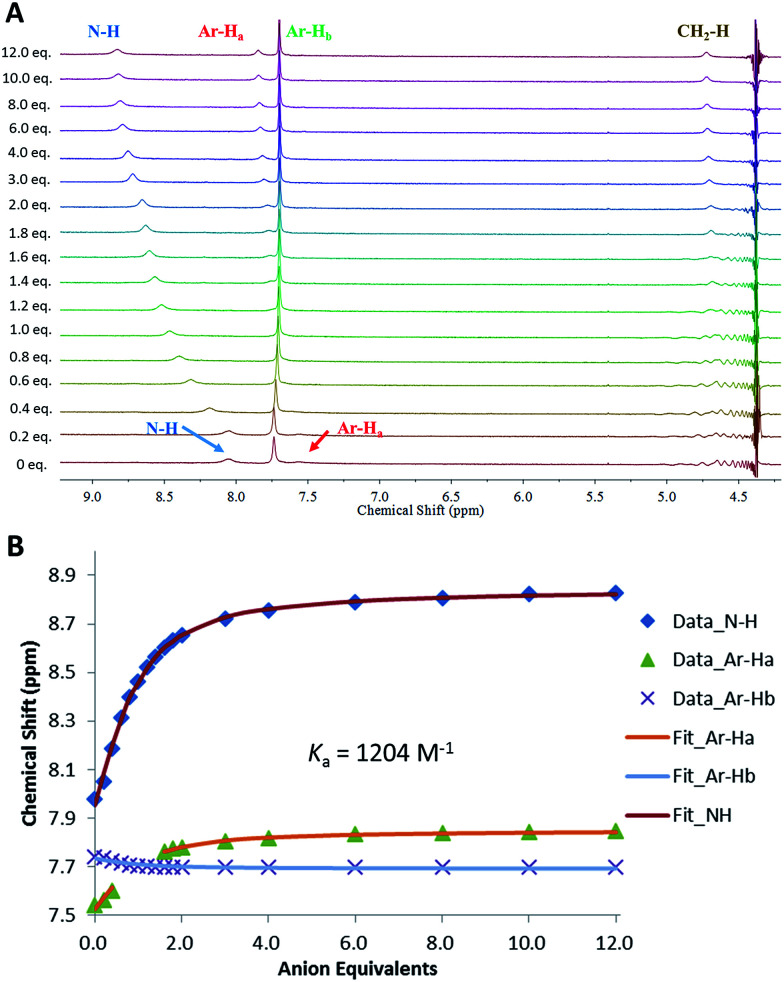
(A): ^1^H NMR titration of compound 6 with (TBA)_2_SO_4_ in 1 : 1 (v/v) H_2_O–DMSO-*d*_6_ mixture (pH 3.2) with 2.5 mM phosphates, 10 mM TBACl, 250 mM TBANO_3_ at 300 K. (B): Comparison isotherms of squaramide NH, aromatic protons (Ar-H_a_, H_b_) in the presence of increasing concentrations of SO_4_^2−^. Estimated error in *K*_a_ < 15%.

Following the observation that 6 shows significantly higher affinity for sulfate than for other anions, we performed binding studies of 6 in aqueous mixtures mimicking the anion compositions in both nuclear waste (Fig. 8) and plasma (see ESI†). Notably, in these more complex mixtures, the solubility of 6 increased, such that experiments in 1 : 1 DMSO-*d*_6_ : H_2_O mixtures were possible. This is attributed to binding of 6 to the anionic species already present in solution. In a 1 : 1 v/v mixture of DMSO-*d*_6_ : aqueous plasma electrolytes (20 mM Tris buffer, pH 7.4, 1.5 mM H_2_PO_4_^−^/HPO_4_^2−^, 106 mM Cl^−^, 28 mM H_2_CO_3_/HCO_3_^−^) TEG-MSQR 6 was still able to bind to sulfate ions (1 equiv. SO_4_^2−^ = 2.5 mM, final SO_4_^2−^ concentration = 30 mM) with a distinct shift of the aromatic proton observed in the NMR titration experiment (the squaramide protons were too broad to observe under these conditions), giving an apparent *K*_a_ = 438 M^−1^ (±15%) when fit to a 1 : 1 binding model. Furthermore, a titration of 6 with sulfate (1 equiv. SO_4_^2−^ = 2.5 mM, final SO_4_^2−^ concentration = 30 mM) performed in mimicked nuclear waste solution (1 : 1 DMSO-*d*_6_ : H_2_O, pH = 3.2, 2.5 mM phosphates, 10 mM TBACl, 250 mM TBANO_3_) gave an apparent *K*_a_ for SO_4_^2−^ = 1204 M^−1^ (±15%), with distinct downfield shifts of the squaramide NH protons as well as shifts for the aromatic protons. These results directly showcase the ability of MSQs to bind sulfate in aqueous media and in the presence of a large range of interferants, and demonstrate the potential applications of MSQs in sulfate separation from nuclear wastes and in plasma sulfate assays.

## Experimental

### General procedures for macrocycle synthesis

#### Procedure 1

A solution of the appropriate diamine (1 eq.) in EtOH (5 mL) was added to a stirred solution of 7 (3 eq.) in EtOH (5 mL) before the addition of Et_3_N (5 eq.). The reaction mixture was stirred at room temperature overnight. The resulting precipitate was collected by filtration, and washed with EtOH then Et_2_O to yield the product as a beige/yellow solid.

#### Procedure 2

The appropriate Boc-protected amine was dissolved in a solution of TFA/DCM (1 : 1) and the mixture was stirred at room temperature for 1 hour then concentrated under reduced pressure. The resulting amine was used without further purification.

#### Procedure 3

A solution of the appropriate diamine or di(squarate ester) (1 eq.) in EtOH (150 mL) was heated to 90 °C. Et_3_N (5 eq.) was added then the appropriate diamine or disquarate in EtOH (30 mL) was added dropwise at 1.7 mL h^−1^*via* syringe pump. The mixture was stirred for 72 h. The resulting precipitate was isolated by filtration and washed with EtOH then Et_2_O to yield the product as a beige/yellow solid.

## Conclusions

In summary, we have successfully synthesized a new family of squaramide based macrocycles *via* a facile synthetic process, and shown that these can be readily functionalised to modulate their solubility. Evaluation of the anion-binding affinity and selectivity of [2]- and [3]-MSQs revealed remarkable anion binding in a range of conditions, from 0.5% v/v H_2_O/DMSO-*d*_6_ to 50% v/v H_2_O/DMSO-*d*_6_ where the *meta*-substituted derivatives 1, 2, 5, 6 were found to be extremely potent and selective ligands for SO_4_^2−^ in highly competitive conditions, with 6 showing a better ability to discriminate between sulfate and chromate ions than the sulfate binding protein. Experiments under simulated competitive conditions reinforce the opportunity for applications of these sulfate-selective receptors in both nuclear waste remediation and plasma sulfate detection. We are currently investigating these and related systems in more detail, and will report on these studies in due course.

## Supplementary Material

SC-007-C6SC01011C-s001

SC-007-C6SC01011C-s002
