# Supplementary material for: Macrocyclic squaramides: anion receptors with high sulfate binding affinity and selectivity in aqueous media
Source: Chem Sci. 2016 Apr 1;7(7):4563–72. doi: 10.1039/c6sc01011c (PMC6014085; doi:10.1039/c6sc01011c)
Supplement: SC-007-C6SC01011C-s001 [file SC-007-C6SC01011C-s001.pdf]

Electronic Supplementary Material (ESI) for Chemical Science.
This journal is © The Royal Society of Chemistry 2016

Supporting Information

Macrocyclic Squaramides: Anion Receptors with High Sulfate Binding Affinity and Selectivity in Aqueous Media

Lei Qin [a,‡], Anna Hartley [a], Peter Turner[a], Robert B. P. Elmes[a,b,‡] and Katrina A. Jolliffe [a]*

[a] School of Chemistry, The University of Sydney, Sydney, NSW 2006, Australia.

E-mail: kate.jolliffe@sydney.edu.au; Fax: +61 2 9351 3329; Tel: +61 2 9351 2297

[b] Current address: Department of Chemistry, Maynooth University,

National University of Ireland Maynooth, Co. Kildare, Ireland.

E-mail: robert.elmes@nuim.ie; Fax: +353 1708 3815; Tel: +353 1708 4615

Contents

1. **Experimental details and references** **S3**
2. **^{1}H and ^{13}C NMR spectra of new compounds** **S15**
3. **High resolution Mass Spectra of anion complexes with receptors** **S36**
4. **Anion binding studies** **S41**
    - **4.1** General procedures **S41**
    - **4.2** Binding studies of compound **1** **S41**
    - **4.3** Binding studies of compound **3** **S47**
    - **4.4** Binding studies of compound **2** **S49**
    - **4.5** Binding studies of compound **4** **S54**
    - **4.6** Binding studies of compound **5** **S55**
    - **4.7** Binding studies of compound **6** **S62**
5. **Modelled structures** **S75**
6. **X-ray crystallography summary and references** **S76**

General remarks

^{1}H NMR spectra were recorded using a Bruker Avance III 500 at a frequency of 500.13 MHz, and are reported as parts per million (ppm) with DMSO-d_6 (δ_H 2.50 ppm) as an internal reference. The data are reported as chemical shift (δ), multiplicity (br = broad, s = singlet, d = doublet, dd = doublet of doublets, dt = doublet of triplets, t = triplet, q = quartet, quint = quintet, m = multiplet, br s = broad singlet), coupling constant (*J* Hz) and relative integral. ^{13}C NMR spectra were recorded using a Bruker Avance III 500 at a frequency of 125.76 MHz and are reported as parts per million (ppm) with DMSO-d_6 (δ_H 39.52 ppm) as an internal reference. High resolution ESI spectra were recorded on a Bruker BioApex Fourier Transform Ion Cyclotron Resonance mass spectrometer (FTICR) with an Analytica ESI source, operating at 4.7 T or a Bruker Daltonics Apex Ultra FTICR with an Apollo Dual source, operating at 7 T. Analytical TLC was performed using precoated silica gel plates (Merck Kieselgel 60 F254). Tetrabutylammonium salts were used as supplied and were stored in a vacuum desiccator over silica drying beads and phosphorous pentoxide. 3,4-Diethoxycyclobut-3-ene-1,2-dione (diethyl squarate, **7**) was synthesised as previously described[1], and all other reagents were commercially available and used as supplied.

The following compounds were synthesised according to literature procedures: *tert*-butyl 3-(aminomethyl)benzylcarbamate (**8**)[2], *tert*-butyl ((6-(aminomethyl)pyridin-2-yl)methyl)carbamate (**9**)[2], methyl 3,5-bis(azidomethyl)benzoate (**16**)[3].

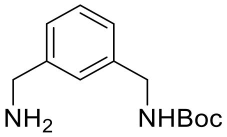

8

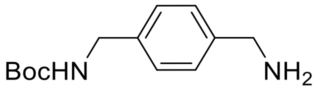

9

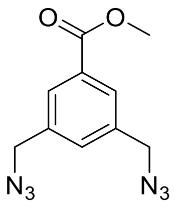

16

General procedure for receptor synthesis:

Procedure 1:

A solution of the appropriate diamine (1 eq.) in EtOH (5 mL) was added to a stirred solution of **7** (3 eq.) in EtOH (5 mL) before the addition of Et_3N (5 eq.). The reaction mixture was stirred at room temperature overnight. The resulting precipitate was collected by filtration, and washed with EtOH then Et_2O to yield the product as a beige/yellow solid.

Procedure 2:

The appropriate Boc-protected amine was dissolved in a solution of TFA / DCM (1:1) and the mixture was stirred at room temperature for 1 hour then concentrated under reduced pressure. The resulting amine was used without further purification.

Procedure 3:

A solution of the appropriate diamine or di(squarate ester) (1 eq.) in EtOH (150 ml) was heated to 90°C. Et_3N (5 eq.) was added then the appropriate diamine or disquarate in EtOH (30 ml) was added dropwise at 1.7 ml/hr *via* syringe pump. The mixture was stirred for 72 hrs. The resulting precipitate was isolated by filtration and washed with EtOH then Et_2O to yield the product as a beige/yellow solid.

Synthesis

Compound 10

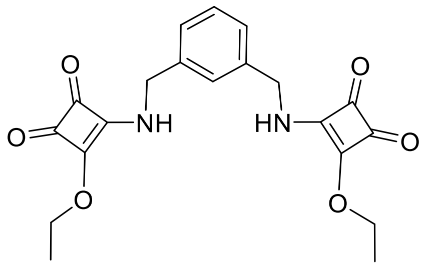

1,3-Phenylenedimethanamine **8** (0.20 g, 1.47 mmol) was reacted with diethyl squarate **7** (0.50 g, 2.94 mmol) according to Procedure 1 to yield **10** as a beige solid. (0.260 g, 46%). **M.p.** 200 - 205 °C (decomp.); **^{1}H NMR** (500 MHz, DMSO-d_6): 1.36 (m, 6 H), 4.46 (m, 8 H), 7.23 (d, J = 7.2 Hz, 3 H), 7.36 (t, J= 7.6 Hz, 1 H), 9.06 (s, 1 H), 9.27 (s, 1 H); **^{13}C NMR** (125 MHz, DMSO-d_6): 16.19, 47.30, 47.79, 69.54, 126.90, 127.23, 129.55, 139.19, 139.49, 172.60, 173.28, 177.34, 177.90, 182.99, 189.70, 190.00 (Note: Due to slow interconversion of conformers in DMSO, a number of signals in the ^{13}C spectra are doubled); **HRMS** (ESI) calcd. for $C_{20}H_{20}N_2O_6Na$ $[M + Na]^+$ 407.1214, found 407.1213; **ν_{max} (film)/cm^{-1}:** 3167 (broad), 1797, 1696, 1573, 1500, 1417, 1354.

Compound 11

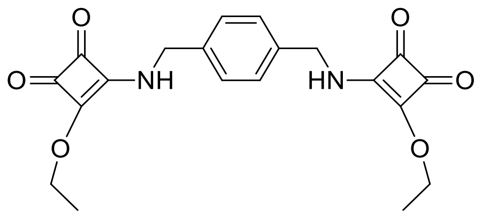

1,4-Phenylenedimethanamine **9** (0.1 g, 0.735 mmmol) was reacted with diethyl squarate **7** (0.25 g, 1.47 mmol) according to Procedure 1 to yield **11** as a beige solid. (0.271 g, 96%). **M.p.** 190 - 195 °C (decomp.); **^{1}H NMR** (500 MHz DMSO-*d$_6$*): 1.36 (m, 6 H), 4.69 (m, 8 H), 7.32 (s, 4 H), 9.12 (s, 1 H); **^{13}C NMR** (125 MHz, DMSO-*d$_6$*): 15.59, 46.51, 46.99, 68.91, 127.72, 137.39, 137.72, 172.00, 172.64, 176.71, 177.35, 182.11, 182.37, 189.07, 189.43 (Note: Due to slow interconversion of conformers in DMSO, a number of signals in the ^{13}C spectra are doubled); **HRMS (ESI)** calcd. for $C_{20}H_{20}N_2O_6Na$ $[M + Na]^+$ 407.1214, found 407.1212; **ν_{max} (film)/cm^{-1}:** 3203 (broad), 2931, 1797, 1698, 1594, 1493, 1437, 1386, 1341, 1253, 1090.

Compound 14

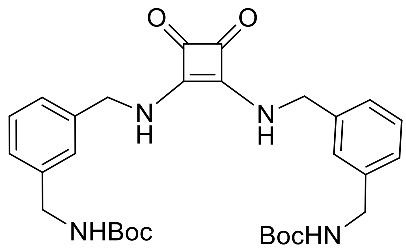

Tert-butyl 3-(aminomethyl)benzylcarbamate **12** (0.226 g, 0.958 mmol) was reacted with diethyl squarate **7** (0.082 g, 0.479 mmol) according to Procedure 1 to yield **14** as a beige solid. (0.232 g, 88%). **M.p.** 210 - 216 °C (decomp.); **^{1}H NMR** (500 MHz, $CDCl_3$): 1.38 (s, 18 H), 4.11 (d, *J* = 6.1 Hz, 4 H), 4.70 (s, 4 H), 7.17 (t, *J* = 7.7 Hz, 6 H), 7.30 (t, *J* = 7.8 Hz, 2 H), 7.37 (t, *J* = 5.8 Hz, 2 H), 7.67 (s, 2 H); **^{13}C NMR** (125 MHz, $CDCl_3$): 28.21, 43.27, 46.82, 77.79, 125.86, 125.95, 128.55, 138.77, 140.70, 155.79, 167.37, 182.56; 1 signal obscured or overlapping; **HRMS (ESI)** calcd. for $C_{30}H_{38}N_4O_6Na$ $[M + Na]^+$ 573.2684, found 573.2684; **ν_{max} (film)/cm^{-1}:** 3318, 2980 (broad), 1799, 1682, 1644, 1576, 1543, 1435, 1363, 1278, 1174.

Compound 15

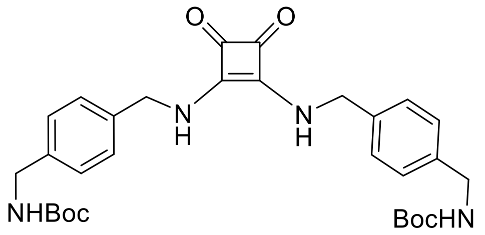

Tert-butyl 4-(aminomethyl)benzylcarbamate **13** (0.233 g, 0.992 mmol) was reacted with diethyl squarate **7** (0.084 g, 0.496 mmol) according to Procedure 1 to yield **15** as a beige solid (0.273 g, 100%). **M.p.** 240 - 244 °C (decomp.); **^{1}H NMR** (500 MHz, $CDCl_3$): 1.38 (s, 18 H), 4.09 (d, J = 6.1 Hz, 4 H), 4.67 (s,4 H), 7.21 (t, J = 2.9 Hz, 8 H), 7.35 (t, J = 5.7 Hz, 2 H), 7.66 (s, 2 H); **^{13}C NMR** (125 MHz, $CDCl_3$): 28.25, 43.12, 46.59, 77.80, 127.22, 127.46, 137.26, 139.44, 155.79, 167.52, 182.62; **HRMS (ESI)** calcd. for $C_{30}H_{38}N_4O_6Na$ $[M + Na]^+$ 573.2684, found 573.2683; **ν_{max} (film)/cm^{-1}:** 3341 (broad), 3154, 2966, 1689, 1642, 1565, 1542, 1430, 1345, 1249.

Macrocycle 1

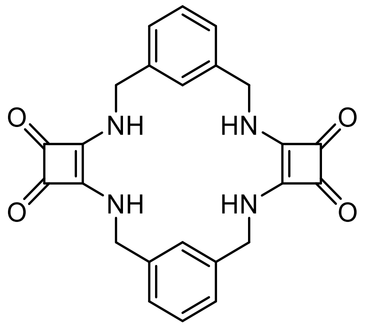

Di(squarate ester) **10** (0.139 g, 0.362 mmol) was reacted with diamine **8** (0.049 mmol, 0.362 mmol) according to Procedure 3 to yield receptor **1** as a beige solid. (0.135 g, 87%). **M.p.** 296 - 300 °C (decomp.); **^{1}H NMR** (500 MHz, DMSO-d_6): 4.75 (s, 3 H), 4.81 (d, 5 H), 7.22 (m, 8 H), 7.87 (s, 3 H), 8.51 (s, 1 H); **^{13}C NMR** (125 MHz, DMSO-d_6): 46.25, 122.69, 125.94, 128.34, 140.14, 167.44, 182.39; **HRMS (ESI)** calcd. for $C_{24}H_{20}N_4O_4Na$ $[M + Na]^+$ 451.1377, found 451.1380; **ν_{max} (film)/cm^{-1}:** 3275 (broad), 3166, 1801, 1665, 1572, 1480, 1418, 1346, 691.

Macrocycle 3

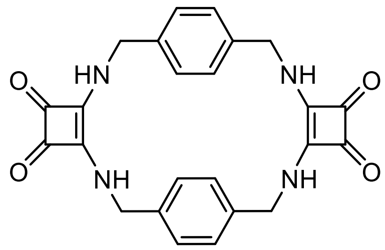

Di(squarate ester) **11** (0.099 g, 0.259 mmol) was reacted with diamine **9** (0.035 g, 0.259 mmol) according to Procedure 3 to yield receptor **3** as a beige solid. (0.103 g, 93%). **M.p.** 276 – 280 °C (decomp.); **^{1}H NMR** (500 MHz, DMSO-d_6): 4.29 – 4.65 (m, 8 H), 6.81 – 7.29 (m, 8 H), 7.80 (s, 1 H), 8.14 (s, 1 H), 8.56 (s, 2 H); **^{13}C NMR** (125 MHz, DMSO-d_6): 46.40, 47.42, 127.26, 128.27, 129.49, 139.21, 167.18, 170.01, 183.98; **HRMS** (ESI) calcd. for $C_{24}H_{20}N_4O_4Na$ $[M + Na]^+$ 451.1377, found 451.1378; **ν_{max} (film)/cm^{-1}:** 3170 (broad), 1649, 1556, 1493, 1018.

Macrocycle 2

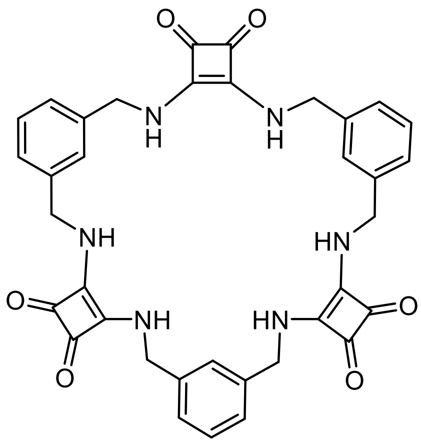

Compound **14** (0.072 g, 0.13 mmol) was deprotected according to Procedure 2 and then the resulting amine was immediately reacted with di(squarate ester) **10** (0.050 g, 0.13 mmol) according to Procedure 3 to yield receptor **2** as a beige solid. (0.066 g, 79%). **M.p.** 257 – 260 °C (decomp.); **^{1}H NMR** (500 MHz, DMSO-d_6): 4.66 (m, 12 H), 7.22 (br s, 9 H), 7.33 (m, 3 H), 7.62 (br s, 6 H); **^{13}C NMR** (125 MHz, DMSO-d_6): 47.37, 127.19, 127.81, 129.72, 139.92, 167.88, 183.16; **HRMS (ESI)** calcd. for $C_{36}H_{30}N_6O_6Na$ $[M + Na]^+$ 665.2119, found 665.2122; **ν_{max} (film)/cm^{-1}:** 3202 (broad), 1657, 1572, 1458, 1342.

Macrocycle 4

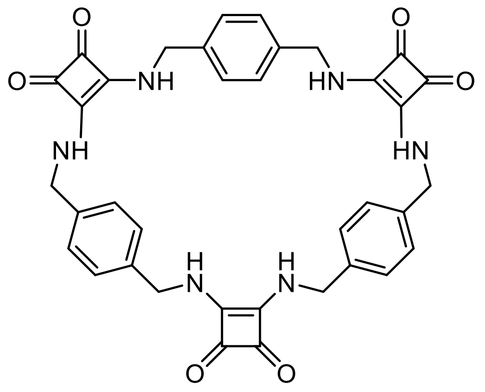

Compound **12** (0.035 g, 0.0641 mmol) was deprotected according to Procedure 2 and the resulting diamine was reacted with di(squarate ester) **11** (0.025g, 0.0641 mmol) according to Procedure 3 to yield receptor **4** as a beige solid. (0.028 g, 68%). **M.p.** 250 – 256 °C (decomp.); **^{1}H NMR** (500 MHz, DMSO-d_6): 4.67 (m, 12 H), 7.00 – 8.00 (m, 12 H), 8.6 – 9.7 (br s, 1 H) NH signals are too broad to get accurate integration; **^{13}C NMR** (125 MHz, DMSO-d_6): 13.44, 19.15, 23.02, 37.76, 46.20, 57.49, 127.58, 129.52, 138.22, 168.00, 182.37, 193.13, 28 signals obscured or overlapping (Note: Due to low solubility, this ^{13}C NMR was run in the presence of 10 equiv. TBA_2SO_4; the TBA's signals were observed at 13.44, 19.15, 23.02, 57.49 ppm); **LRMS** (ESI) calcd. for $C_{36}H_{30}N_6O_6$ $[M]^+$ 642, found 642; **ν_{max} (film)/cm^{-1}:** 3184 (broad), 1642, 1568, 1430, 1346.

2-(2-(2-Methoxyethoxy)ethoxy)ethyl 3,5-bis(azidomethyl)benzoate (**16**)

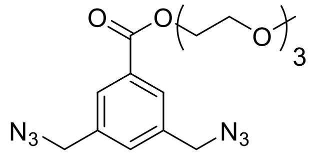

A solution of NaOH (163 mg, 4.1 mmol) in water (5 ml) was added to a solution of methyl 3,5-bis(azidomethyl)benzoate **16** (1.1 g, 4.3 mmol) in THF (40 ml). The resulting mixture was stirred overnight at room temperature and then neutralized by additional of HCl (aq.) (1 M). The solvent was removed under reduced pressure to give a white solid, which was dissolved in anhydrous toluene (20 ml) and then added to a solution of 2-(2-(2-methoxyethoxy)ethoxy)ethan-1-ol (1.1 g, 6.45 mmol) and 4-methylbenzene-sulfonoperoxoic acid (0.2 g, 1.1 mmol) in anhydrous toluene (30 mL) at room temperature. The resulting mixture was stirred at reflux for 24 hours. The solvent was removed under reduced pressure to give a yellow oil. Subjection of this material to flash chromatography (1:4 v/v ethyl acetate/hexane elution) and concentration of the appropriate

fractions (R_f 0.4) gave **16** (0.7 g, 43%) as a beige solid. **M.p.** 30 - 36 °C; **^{1}H NMR** (500 MHz, $CDCl_3$): 3.36 (s, 3 H), 3.65 (t, J = 4.5 Hz, 2 H), 3.68 (t, J = 2.5 Hz, 2 H), 3.72 (dd, J = 3.5, 2 Hz, 2 H), 3.85 (t, J = 4.5 Hz, 2 H), 4.44 (s, 4 H), 4.50 (t, J = 5.0 Hz, 2 H), 7.50 (s, 1 H), 7.98 (s, 2 H); **^{13}C NMR** (125 MHz, $CDCl_3$): 54.2, 59.1, 64.6, 69.2, 70.7, 70.7, 70.8, 72.0, 129.2, 131.5, 132.0, 136.9, 165.8; **HRMS** (ESI) calcd. for $C_{16}H_{22}N_6O_5Na$ $[M + Na]^+$ 401.1544, found 401.1542; **ν_{max} (film)/cm^{-1}:** 2879, 2094, 1718, 1608, 1450, 1349, 1301, 1213.

2-(2-(2-Methoxyethoxy)ethoxy)ethyl 3,5-bis(aminomethyl)benzoate (**17**)

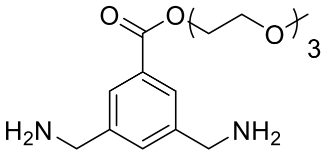

Ph_3P (1.4 g, 5.3 mmol) was added to a solution of compound **16** (0.5 g, 1.3 mmol) in THF (15 ml) and the resulting solution was stirred at room temperature for 2 hours, then 3 ml water was added and the mixture was stirred at room temperature for a further 6 hours. The solvent was then removed under reduced pressure to give a yellow oil. Subjection of this material to flash chromatography (2:18:80 v/v/v triethylamine/methanol/ dichloromethane elution) and concentration of the appropriate fractions (R_f 0.2) gave **17** (0.3 g, 71%) as a beige solid. **M.p.** 57 - 62 °C; **^{1}H NMR** (500 MHz, $CDCl_3$): 3.35 (s, 3 H), 3.52 (br s, 4 H), 3.53 (m, 2 H), 3.60 – 3.70 (m, 6H), 3.82 (t, J = 6.0 Hz, 2 H), 3.91 (s, 4 H), 4.46 (t, J = 6.0 Hz, 2 H), 7,52 (s, 1 H), 7.87 (s, 2H); **^{13}C NMR** (125 MHz, D_2O): 44.0, 58.1, 64.6, 68.7, 69.5, 67.6, 69.9, 71.1, 128.1, 130.4, 132.6, 140.5, 168.2; **HRMS** (ESI) calcd. for $C_{16}H_{26}N_2O_5H$ $[M + H]^+$ 327.1914, found 327.1915; **ν_{max} (film)/cm^{-1}:** 3363 (broad), 2923, 1717, 1302, 1214.

Compound 18

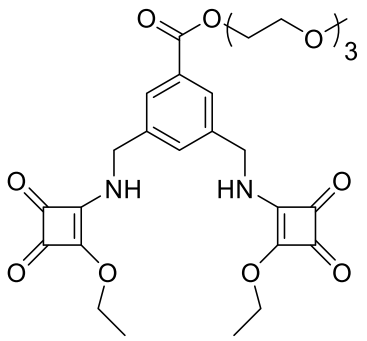

A solution of compound **17** (0.27 g, 0.84 mmol) in EtOH (15 ml) was added to a solution of diethyl squarate **7** (0.32 mg, 2.22 mmol) and Et_3N (0.34 g, 3.36mmol) in EtOH (10 mL) at room temperature and the resulting mixture was stirred at room temperature for 24 hours. The solvent was removed under reduced pressure to give a yellow oil. Subjection of this material to flash chromatography (5:95 v/v methanol/ dichloromethane elution) and concentration of the appropriate fractions (R_f 0.3) gave **18** (0.38 mg, 80%) as a beige solid. **M.p.** 70 - 74 °C (decomp.); **^{1}H NMR** (500 MHz, $CDCl_3$): 1.41 (s, 6 H), 2.33 (s, 1 H), 3.33 (d, J = 15.0Hz, 3 H), 3.51 (t, J = 4.5 Hz,1 H), 3.63(m, 6 H), 3.81 (s, 2 H), 4.45 (s, 2 H), 4.65 (s, 4 H), 4.72 (d, J = 5.0 Hz, 3 H), 5.28 (d, J = 1.0 Hz, 1 H), 7.72 (s, 1 H), 7.88 (s, 2 H), 7.96 (s, 1 H); **^{13}C NMR** (125 MHz, $CDCl_3$): 15.9, 48.0, 59.1, 64.6, 69.3, 70.2, 70.6, 70.7, 70.8, 72.0, 128.6, 131.1, 131.4, 132.0, 138.3, 138.7, 165.9, 172.8, 177.8, 183.4, 189.1, 189.3; **HRMS** (ESI) calcd. for $C_{28}H_{34}N_2O_{11}Na$ $[M + Na]^+$ 597.2055, found 597.2053; **ν_{max} (film)/cm^{-1}:** 3251 (broad), 2923, 1803, 1705, 1592, 1446, 1412, 1382, 1334, 1298, 1212.

Macrocycle 5

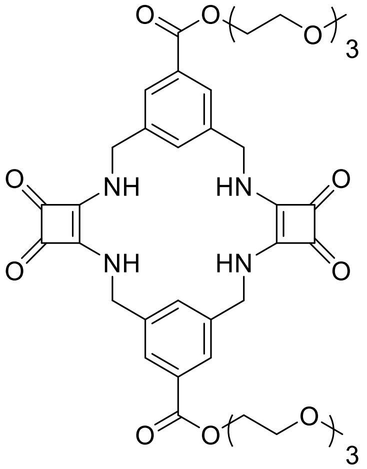

A solution of compound **18** (118 g, 0.21 mmol) in EtOH (30 ml) was added to a solution of diamine **17** (67 mg, 0.21 mmol) and Et_3N (21.2 mg, 0.21mmol) in EtOH (30 mL) at room temperature and the resulting mixture was stirred at room temperature for 2 days. The solvent was removed under reduced pressure to give a yellow oil. Subjection of this material to flash chromatography (7:93 v/v methanol/dichloromethane) and concentration of the appropriate fractions (R_f 0.2) gave the receptor **5** (54 mg, 32%) as a beige solid. **M.p.** 197 - 201 °C (decomp.); **^{1}H NMR** (500 MHz, DMSO-d_6): 3,21 (s, 6 H), 3.41 (d, J = 5.0 Hz, 4 H), 3.52 (dd, J = 9.0, 4.0 Hz, 8 H), 3.52 (d, J = 3.3 Hz, 4 H), 3.75 (s, 4 H), 4.41 (s, 4 H), 4.90 (s, 8 H), 7.46 (brs, 1 H), 7.63 (s, 1 H), 7.80 (s, 4 H), 7.97 (s, 2 H), 8.59 (br s, 2 H); **^{13}C NMR** (125 MHz, DMSO-d_6): 46.6, 58.6, 64.8, 68.9, 70.2, 70.4, 70.5, 71.9, 127.2, 128.1, 130.4, 141.7, 166.0, 168.0, 183.1; **HRMS** (ESI) calcd. for $C_{40}H_{48}N_4O_{14}Na$ $[M + Na]^+$ 831.3059, found 831.3058; **ν_{max} (film)/cm^{-1}:** 3162(broad), 2923, 1799, 1715, 1652, 1566, 1427, 1348, 1299, 1209, 1102, 1029.

2-(2-(2-Methoxyethoxy)ethoxy)ethyl 3-(aminomethyl)-5-(azidomethyl)benzoate (**19**)

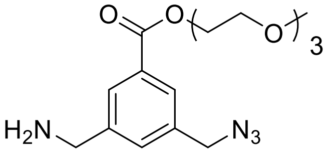

Ph_3P (0.34 g, 1.3 mmol) was added to a solution of diazide **16** (0.5 g, 1.3 mmol) in THF (15 ml) and the mixtrue stirred at room temperature for 2 hours, then 3 ml of water was added and the mixture stirred at room temperature for a further 4 hours. The solvent was removed under reduced pressure to give a yellow oil. Subjection of this material to flash chromatography (1/9/90 v/v/v triethylamine/methanol/ dichloromethane elution) and concentration of the appropriate fractions (R_f 0.3) gave **19** (0.24 g, 52%) as a beige solid. **M.p.** 50 -56 °C; **^{1}H NMR** (500 MHz, $CDCl_3$): 1.75 (br s, 2H), 3.62 (s, 3 H), 3.53 (m, 2 H), 3.63 – 3.73 (m, 6 H), 3.84 (t, J = 6.0 Hz, 2 H), 3.95 (s, 2 H), 4.40 (s, 2 H), 4.49 (t, J = 6.0 Hz, 2H), 7.51 (s, 1 H), 7.88 (s, 1 H), 7.98 (s, 1 H); **^{13}C NMR** (125 MHz, $CDCl_3$): 45.9, 54.3, 59.1, 64.3, 69.2, 70.6, 70.7, 71.9, 77.4, 127.9, 128.3, 130.92, 131.4, 136.1, 144.2, 166.2; **HRMS** (ESI) calcd. for $C_{16}H_{24}N_4O_5Na$ $[M + Na]^+$ 375.1639, found 376.1642; **ν_{max} (film)/cm^{-1}:** 3342 (broad), 2916, 2019, 1717, 1302, 1215, 1109.

Compound 20

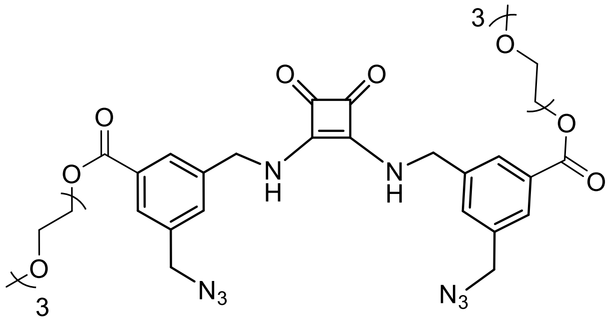

A solution of amine **19** (0.24 g, 0.68 mmol) in EtOH (10 ml) was added to a solution of diethyl squarate **7** (0.28 mg, 1.7 mmol) and Et_3N (0.17 g, 1.7 mmol) in EtOH (5 mL) at room temperature and the resulting mixture was stirred at room temperature for 24 hours. The solvent was removed under reduced pressure to give a yellow oil. Subjection of this material to flash chromatography (5:95 v/v acetate acid/methanol/chloroform elution) and concentration of the appropriate fractions (R_f 0.4) gave the *bis(2-(2-(2-methoxyethoxy)ethoxy)ethyl)5,5'-(((3,4-dioxocyclobut-1-ene-1,2-diyl)bis-(azanediyl))bis(methylene))- bis(3-(azidomethyl)benzoate)* **20** (0.41 g, 77%) as a beige solid. **M.p.** 121 -125 °C; **^{1}H NMR** (500 MHz, $CDCl_3$): 3.30 (s, 6 H), 3.48 – 3.50 (m, 4 H), 3.59 – 3.68 (m, 12 H), 3.80 (t, *J* = 4.5 Hz, 4 H), 3.36 (s, 4 H), 4.42 (t, *J* = 5 Hz, 4 H), 4.89 (d, *J* = 3 Hz, 4 H), 7.50 (s, 2 H), 7.59 (br s, 2 H), 7.88 (s, 2 H), 7.97 (s, 2 H); **^{13}C NMR** (125 MHz, $CDCl_3$): 47.6, 54.2, 59.0, 64.5, 69.3, 70.5, 70.67, 71.9, 128.6, 128.8, 131.3, 132.3, 136.9, 139.5, 165.9, 167.7, 183.0, 1 signal obscured or overlapping; **HRMS** (ESI) calcd. for $C_{36}H_{46}N_8O_{12}Na$ $[M + Na]^+$ 805.3127, found 805.3132; **ν_{max} (film)/cm^{-1}:** 3251 (broad), 2878, 2099, 1719, 1587, 1535, 1245.

Macrocycle 6

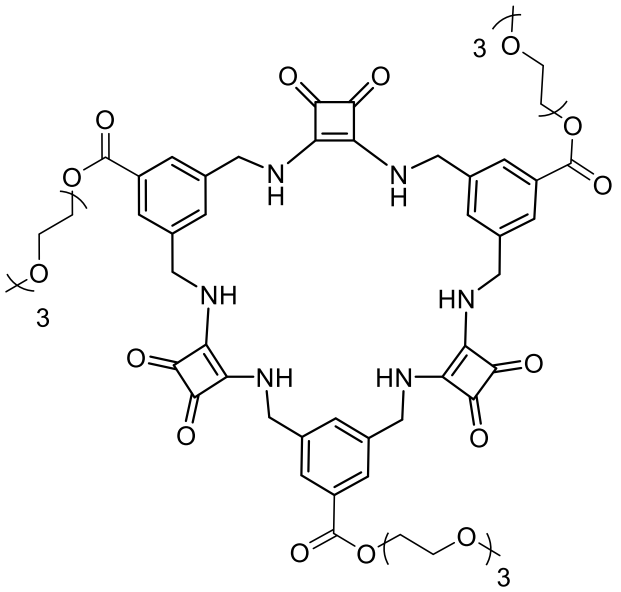

Ph_3P (0.1 g, 1.3 mmol) was added to a solution of compound **20** (50 mg, 0.65 mmol) in THF (5 ml) and the mixture was stirred at room temperature for 2 hours then 3 ml water was added and the mixrture was stirred at room temperature for a further 6 hours. The solvent was removed under reduced pressure to give a beige solid, which was dissolved in EtOH (20 ml), then a solution of compound **18** (33 mg, 0.65 mmol) and Et_3N (66 mg, 0.65 mmol) in EtOH (20 mL) was added and the resulting mixture was stirred at room temperature for 36 hours. The solvent was removed under reduced pressure to give a yellow oil. The resulting mixture was purified by preparative RP-HPLC to give the receptor **6** (27 mg, 35%) as a beige solid. **M.p.** 182 -186 °C (decomp.); **^{1}H NMR** (500 MHz, DMSO-d_6): 3.21 (s, 9 H), 3.40 (dd, J = 3.8, 5.8 Hz, 6 H), 3.52 (m, 12 H), 3.58 (dd, J = 3.2, 5.8 Hz, 6 H), 3.74 (m, 6 H), 4.40 (m, 6 H), 4.76 (d, J = 5.7, 12 H), 7.58 (s, 3 H), 7.87 – 8.00 (br s, 6 H); **^{13}C NMR** (125 MHz, DMSO-d_6): 46.9, 58.6, 64.7, 68.8, 70.1, 70.2, 70.4, 71.8, 128.1, 130.91, 132.0, 140.6, 154.8, 167.9, 183.2; **HRMS** (ESI) calcd. for $C_{60}H_{72}N_6O_{21}Na$ $[M + Na]^+$ 1235.4643, found 1235.4659; **ν_{max} (film)/cm^{-1}:** 3164 (broad), 1717, 1568, 1349, 1218.

Tetrabutylammonium selenate (S1)

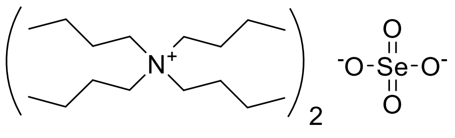

A solution of selenic acid (100 mg, 0.69 mmol) in water (5 ml) was added to a solution of tetrabutylammonium hydroxide (358 mg, 1.38 mmol) in water (5 ml) at room temperature over half an hour, and the resulting mixture was stirred at room temperature for one hour to cool down to room temperature. Then the pH was adjusted to 7.00 by addition of an aqueous solution of TBAOH.. The solvent was removed by lyophilisation to form Tetrabutylammonium selenate as white powder (quantitative yield). **M.p.** 160 -165 $^{\circ}$C (decomp.); **^{1}H NMR** (500 MHz, DMSO-d_6): 3.18 (t, J = 8.4 Hz, 16 H), 1.43 (quint, J = 8.4, 16 H), 1.22 (m, 16 H), 0.748 (t, J = 7.33 Hz, 24 H); **^{13}C NMR** (125 MHz, DMSO-d_6): 58.7, 24.0, 19.57, 13.6; **^{77}Se NMR** ($CDCl_3$, 95 MHz): 1057.9.

Synthesis References:

(1) Rostami, A.; Colin, A.; Li, X. Y.; Chudzinski, M. G.; Lough, A. J.; Taylor, M. S. *J. Org. Chem.* **2010**, *75*, 3983.
(2) Smith, J.; Liras, J. L.; Schneider, S. E.; Anslyn, E. V. *J. Org. Chem.* **1996**, *61*, 8811.
(3) Kuchelmeister, H. Y.; Schmuck, C. *Eur. J. Org. Chem.* **2009**, *2009*, 4480.

Figure S1: ^{1}H NMR (DMSO-d_6, 500 MHz) and ^{13}C NMR (DMSO-d_6, 125 MHz) spectra of **10**.

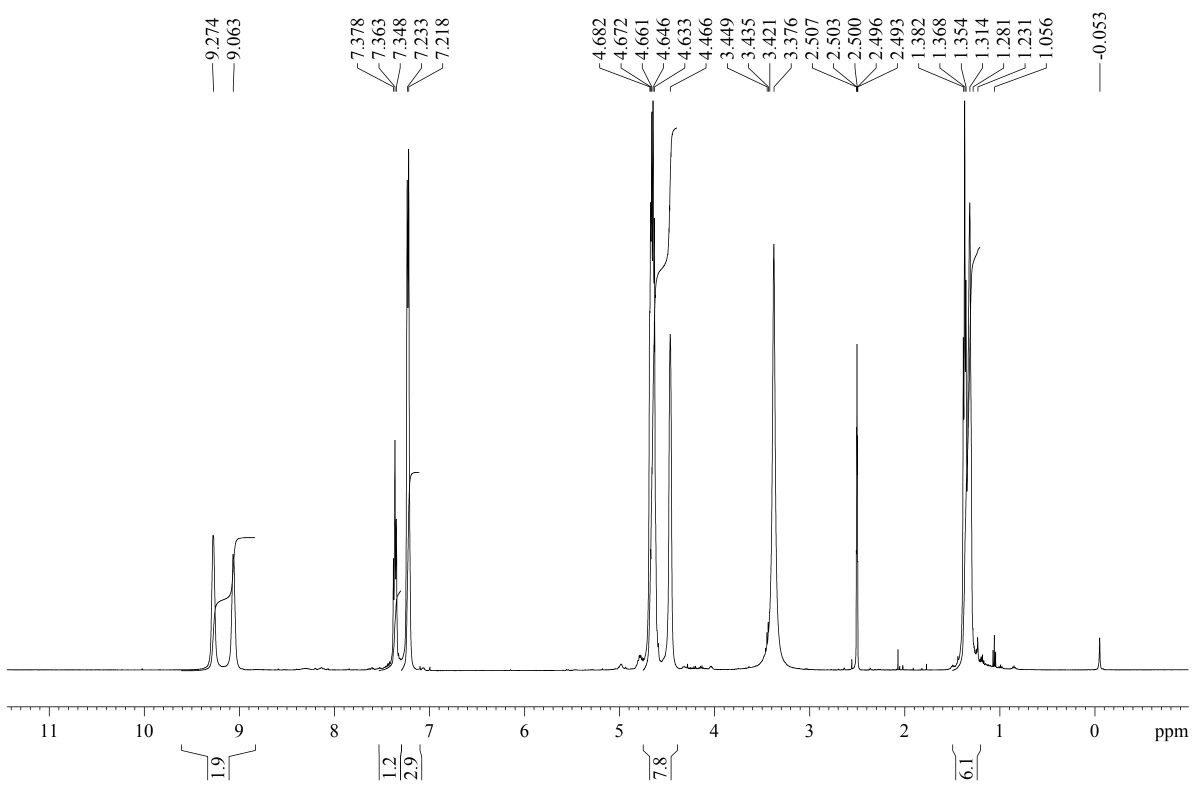

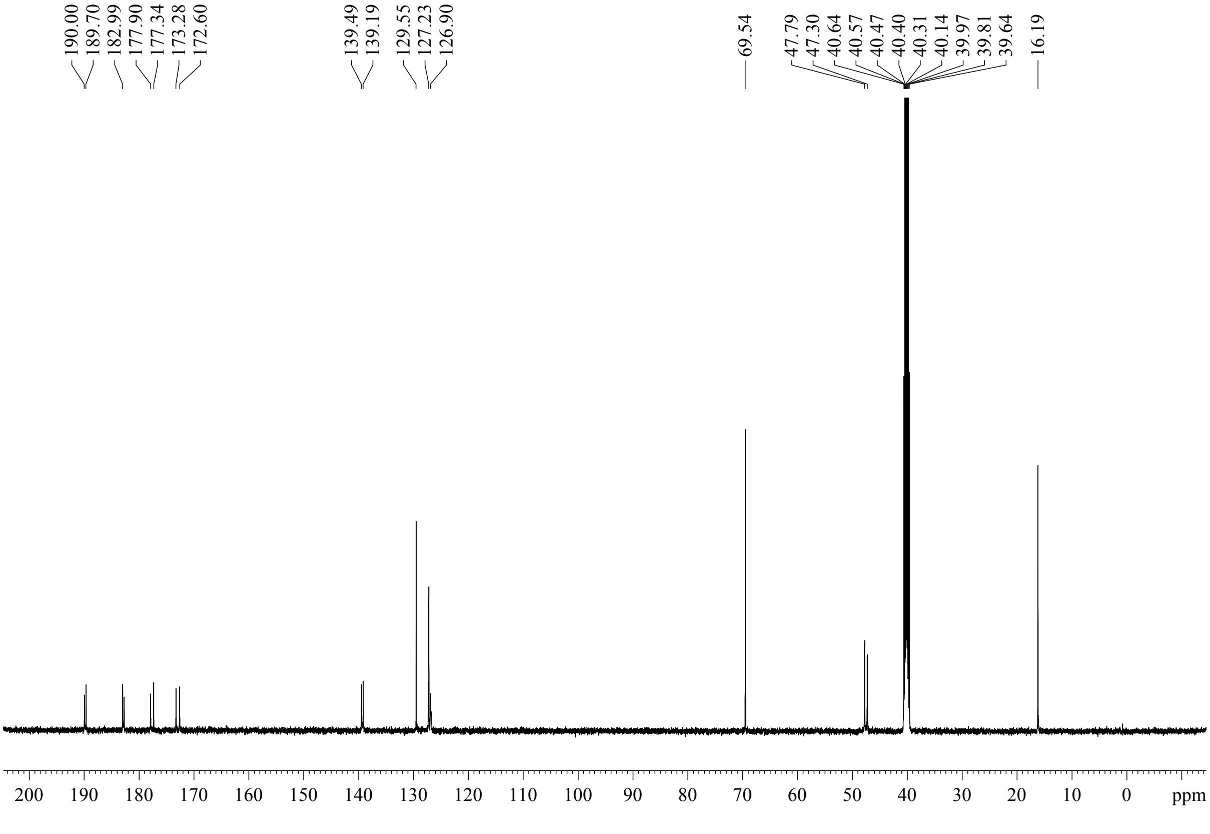

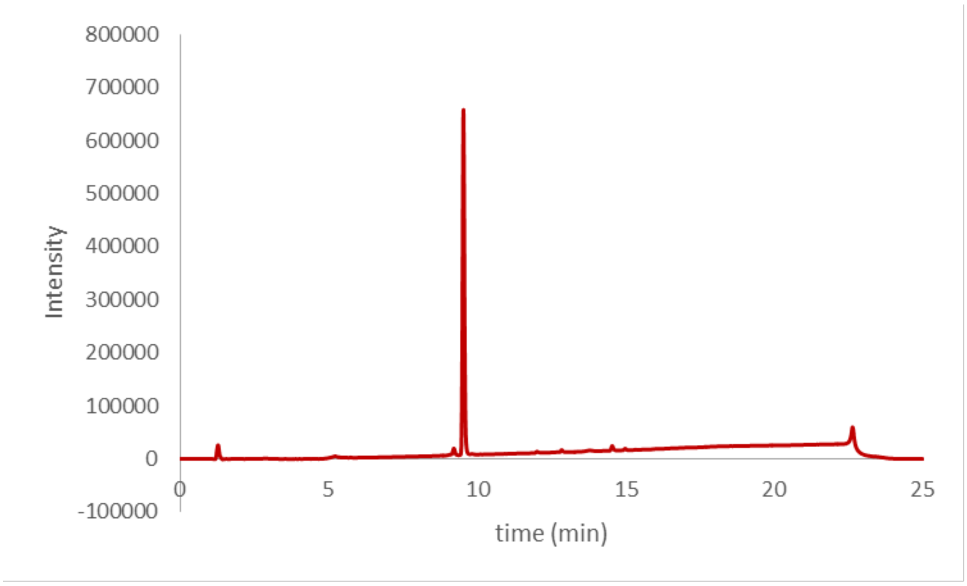

LC trace for compound **10**, method: water/acetonitrile 0 to 100, 15 min; 100% acetonitrile, 15 min.

Figure S2: ^{1}H NMR (DMSO-d_6, 500 MHz) and ^{13}C NMR (DMSO-d_6, 125 MHz) spectra of **11**.

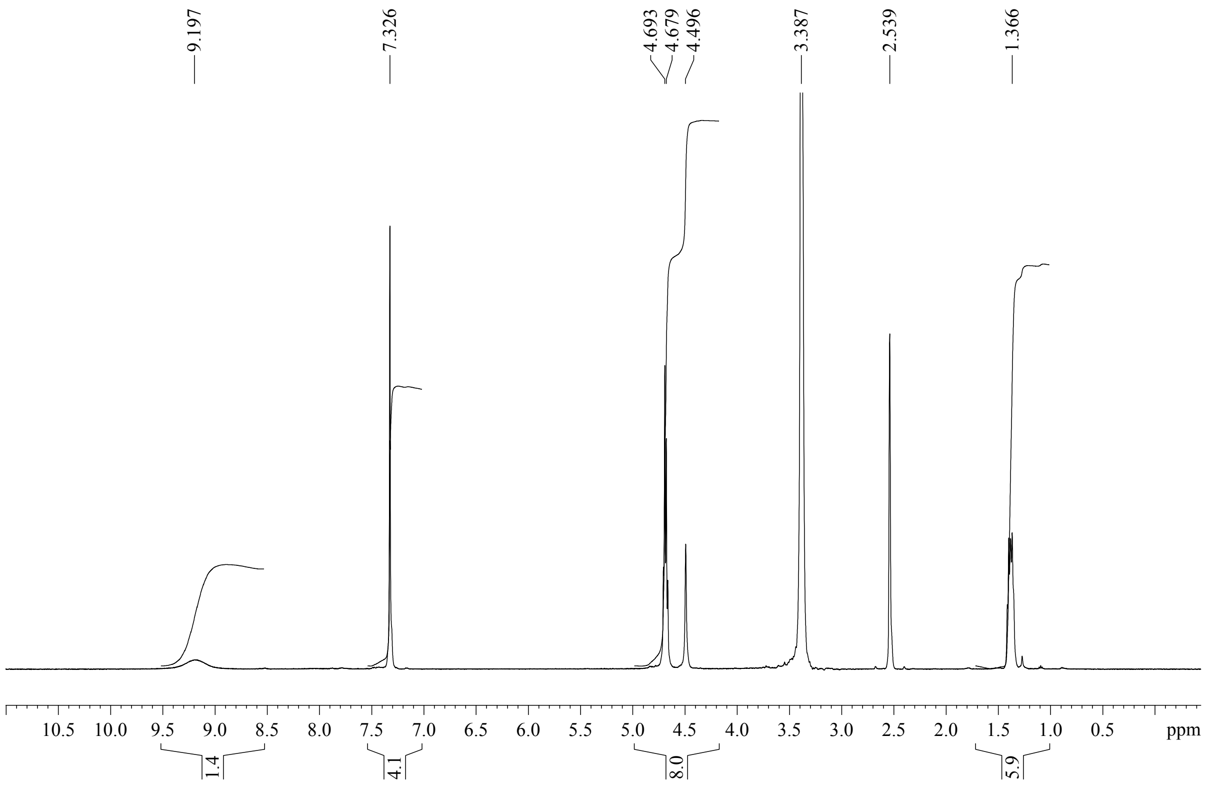

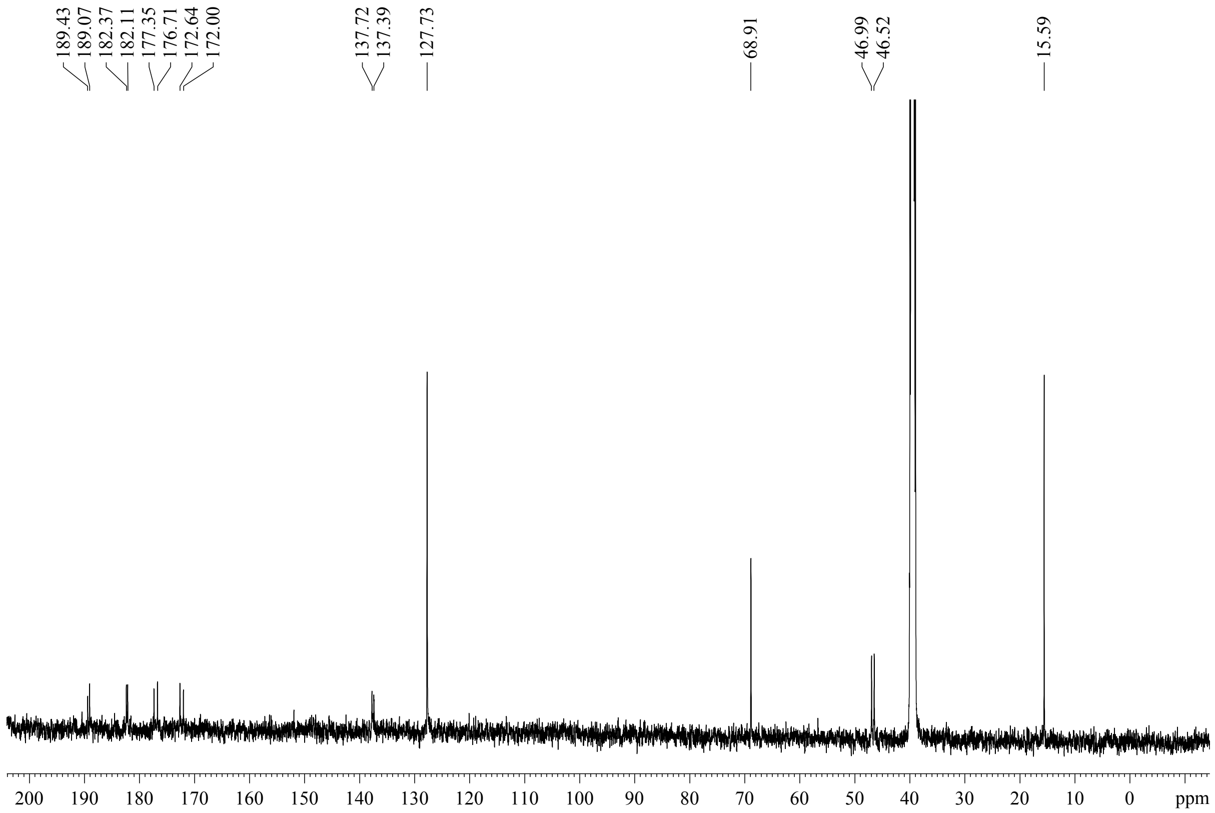

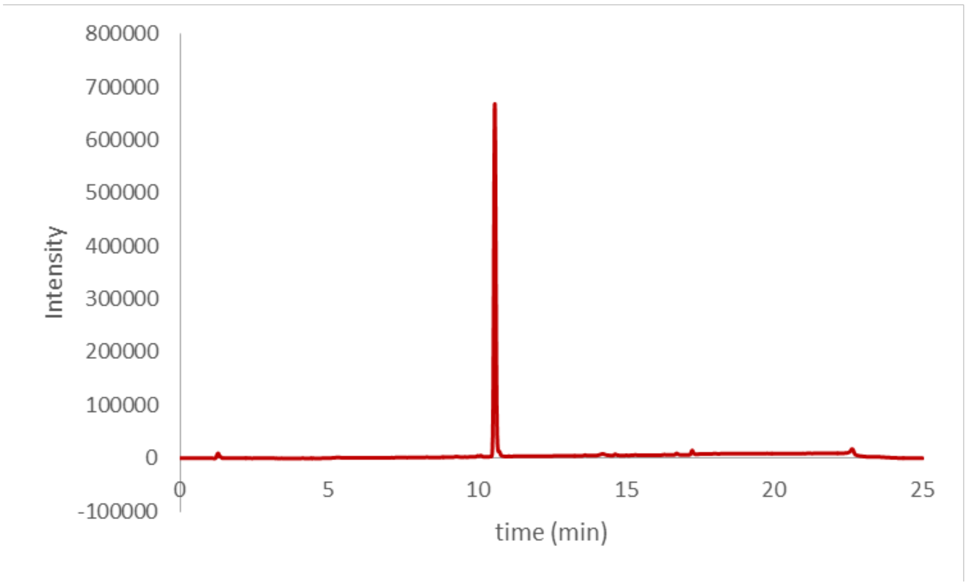

LC trace for compound **11**, method: water/acetonitrile 0 to 100, 15 min; 100% acetonitrile, 15 min.

Figure S3: ^{1}H NMR (DMSO-d_6, 500 MHz) and ^{13}C NMR (DMSO-d_6, 125 MHz) spectra of **14**.

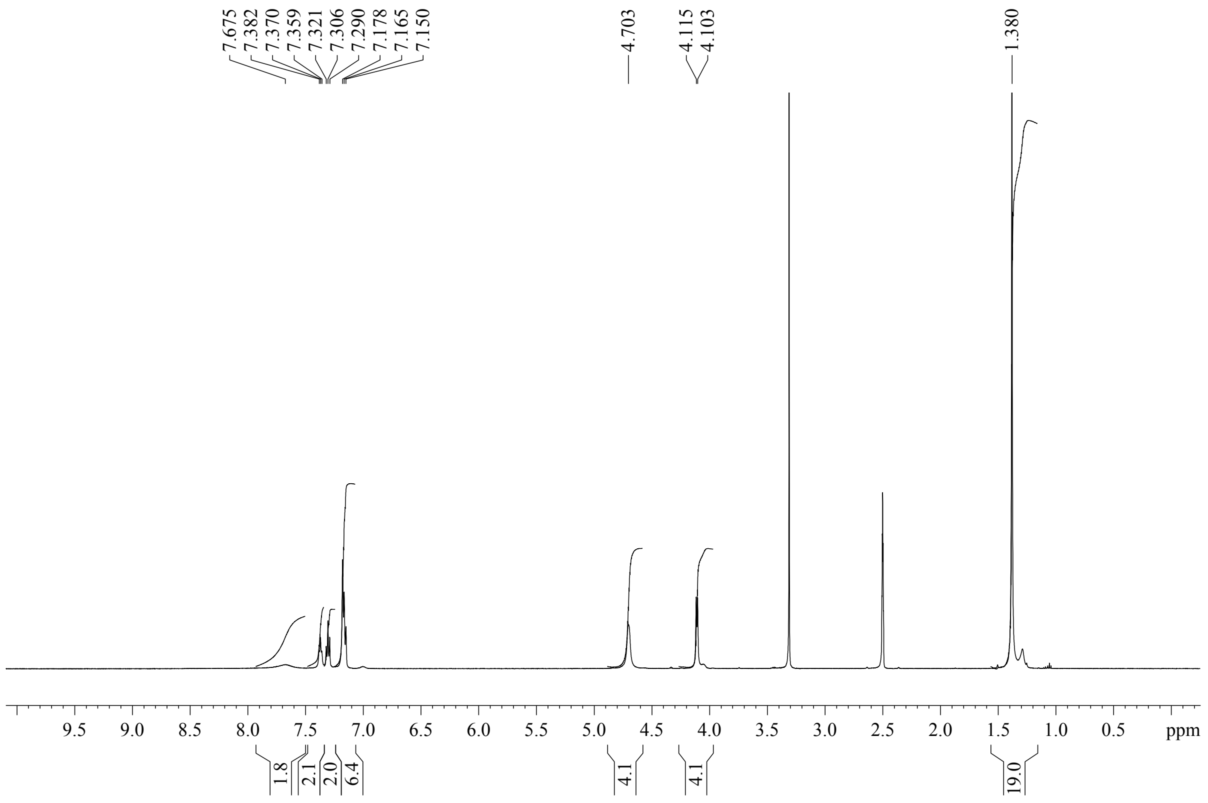

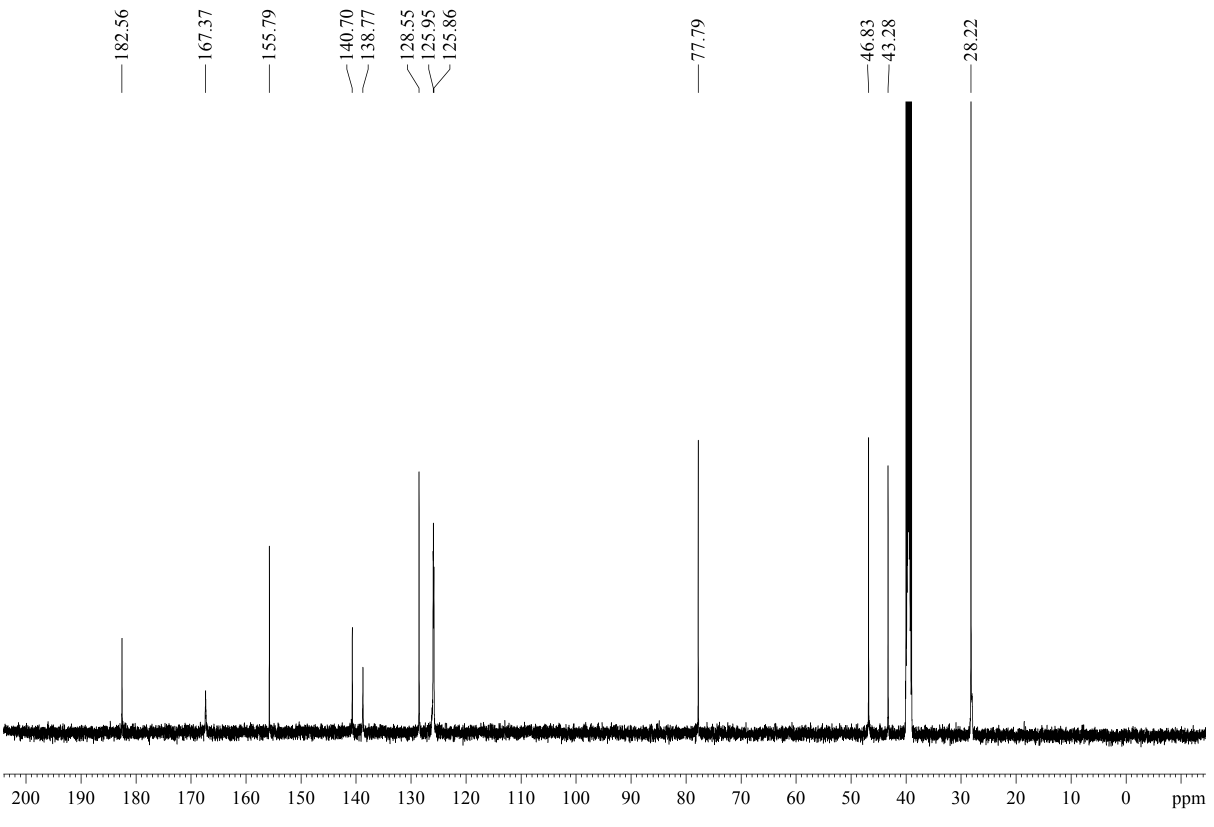

Figure S4: ^{1}H NMR (DMSO-d_6, 500 MHz) and ^{13}C NMR (DMSO-d_6, 125 MHz) spectra of **15**.

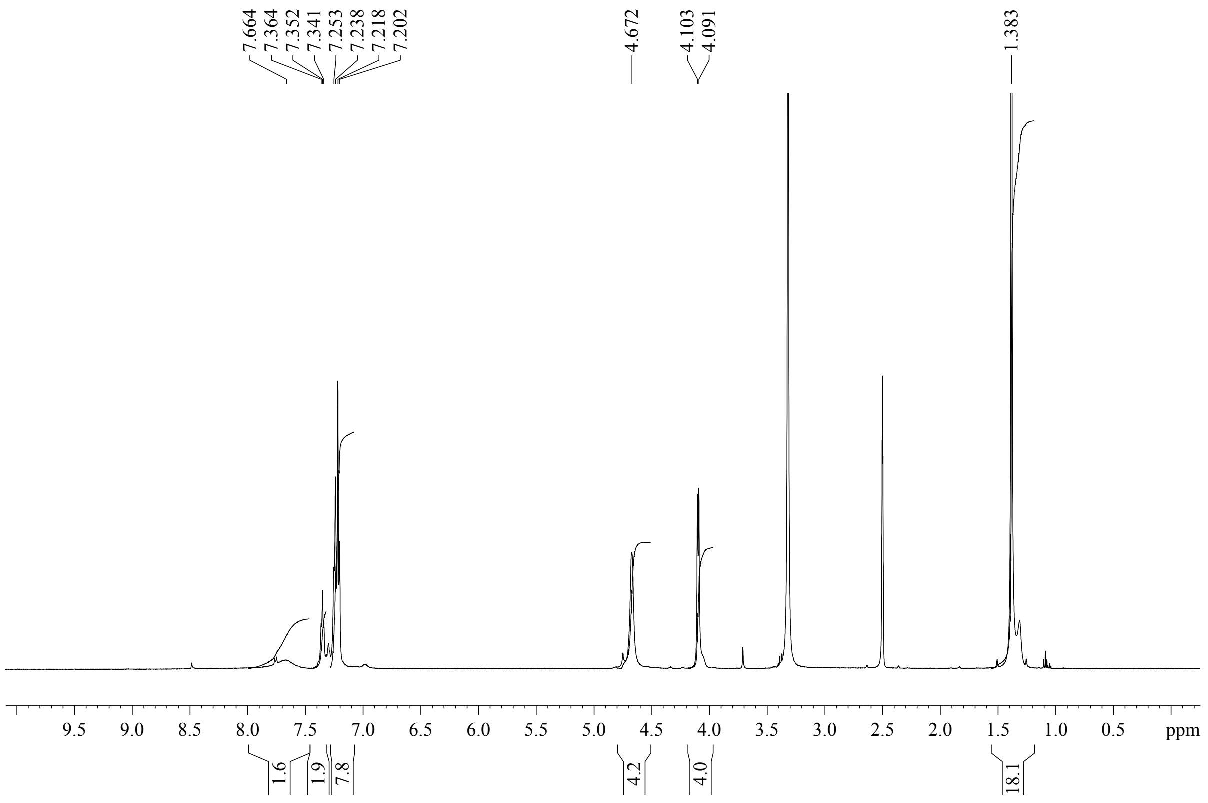

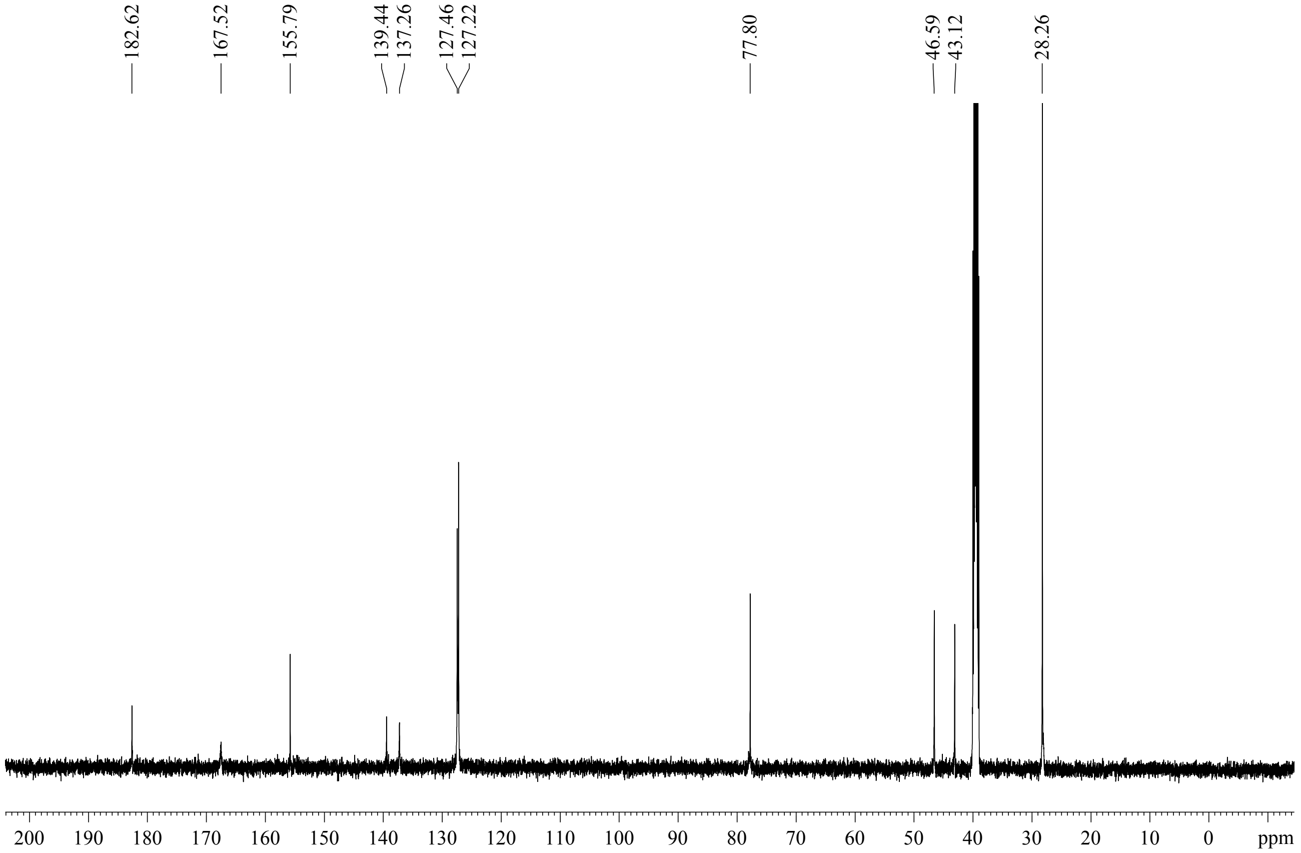

Figure S5: ^{1}H NMR (DMSO-d_6, 500 MHz) and ^{13}C NMR (DMSO-d_6, 125 MHz) spectra of **1**.

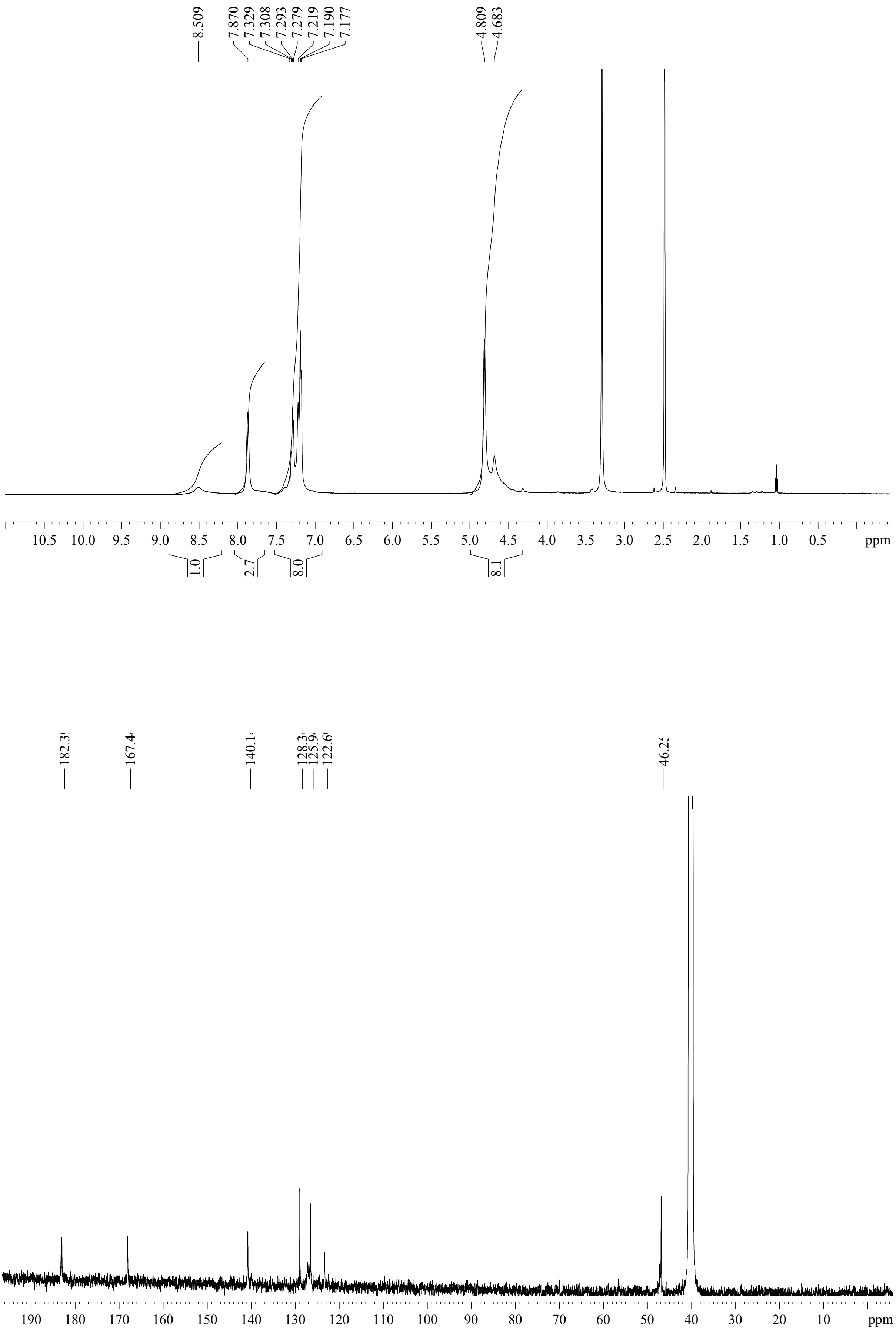

Figure S6: ^{1}H NMR (DMSO-d_6, 500 MHz) and ^{13}C NMR (DMSO-d_6, 125 MHz) spectra of **3**.

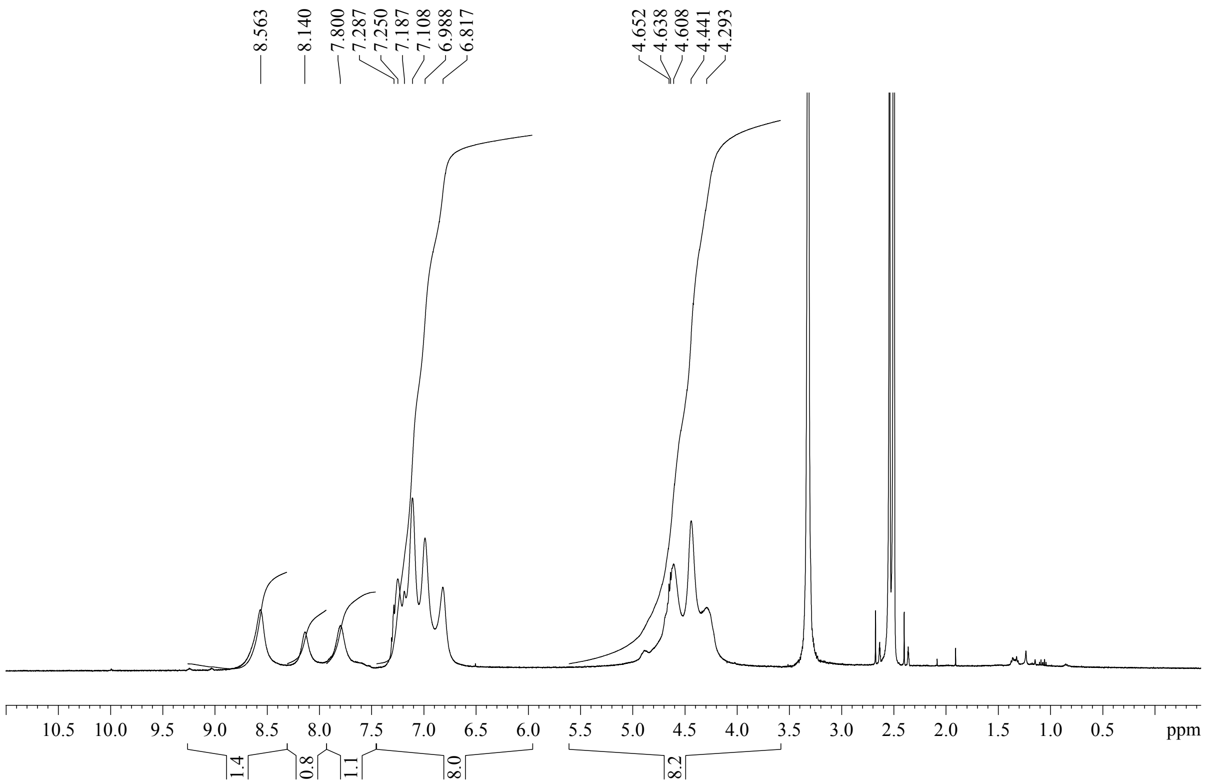

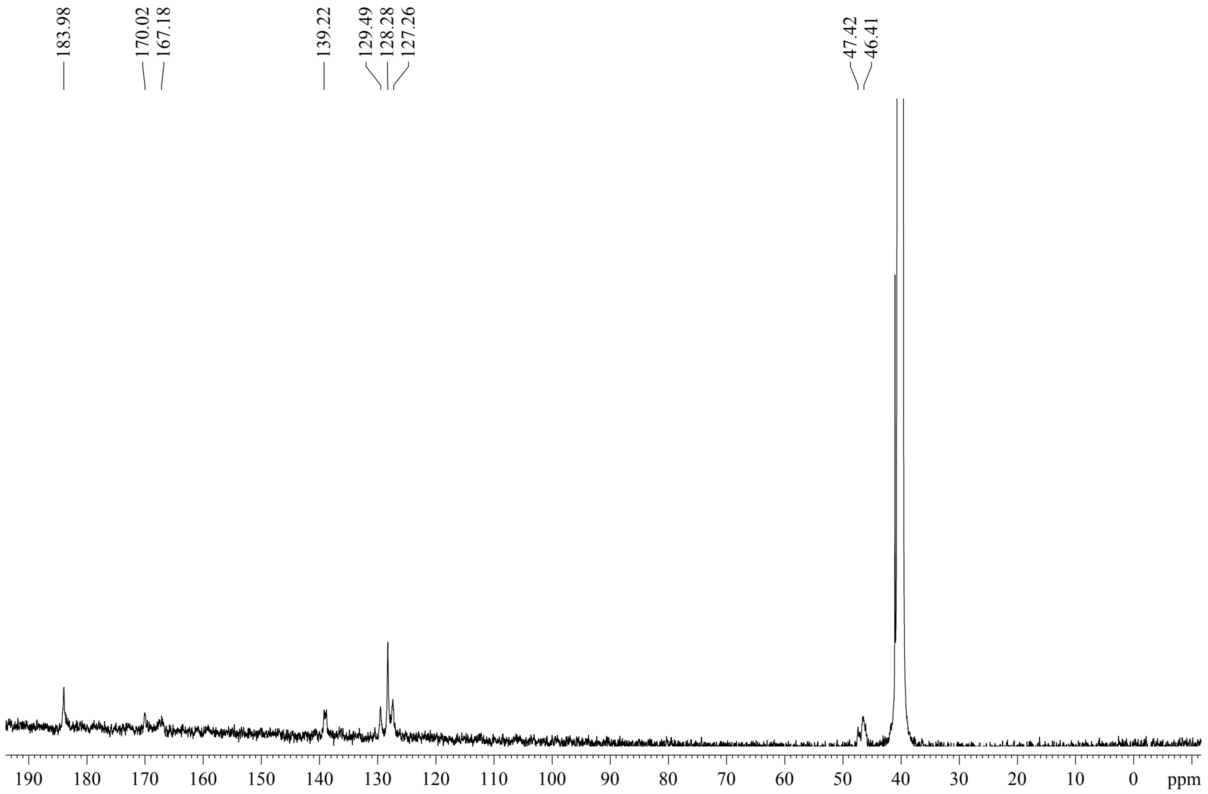

Figure S7: ^{1}H NMR (DMSO-d_6, 500 MHz) and ^{13}C NMR (DMSO-d_6, 125 MHz) spectra of **2**.

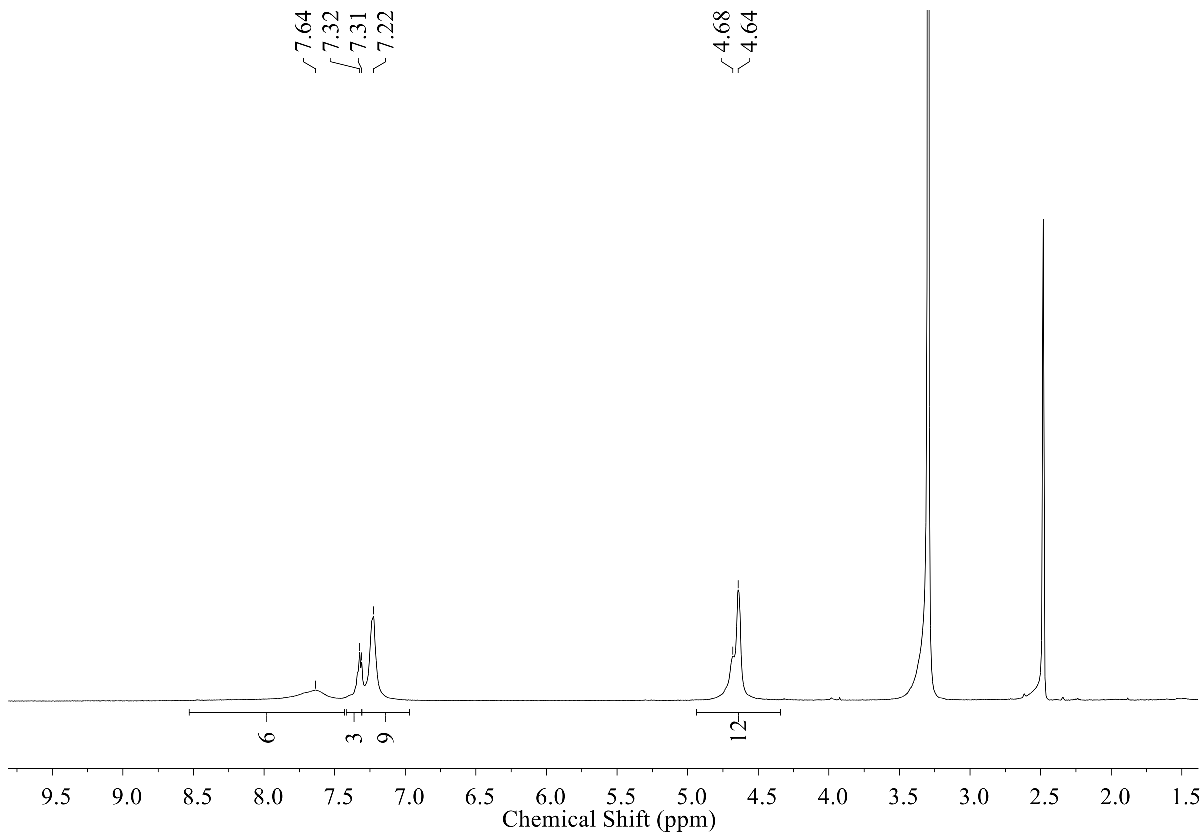

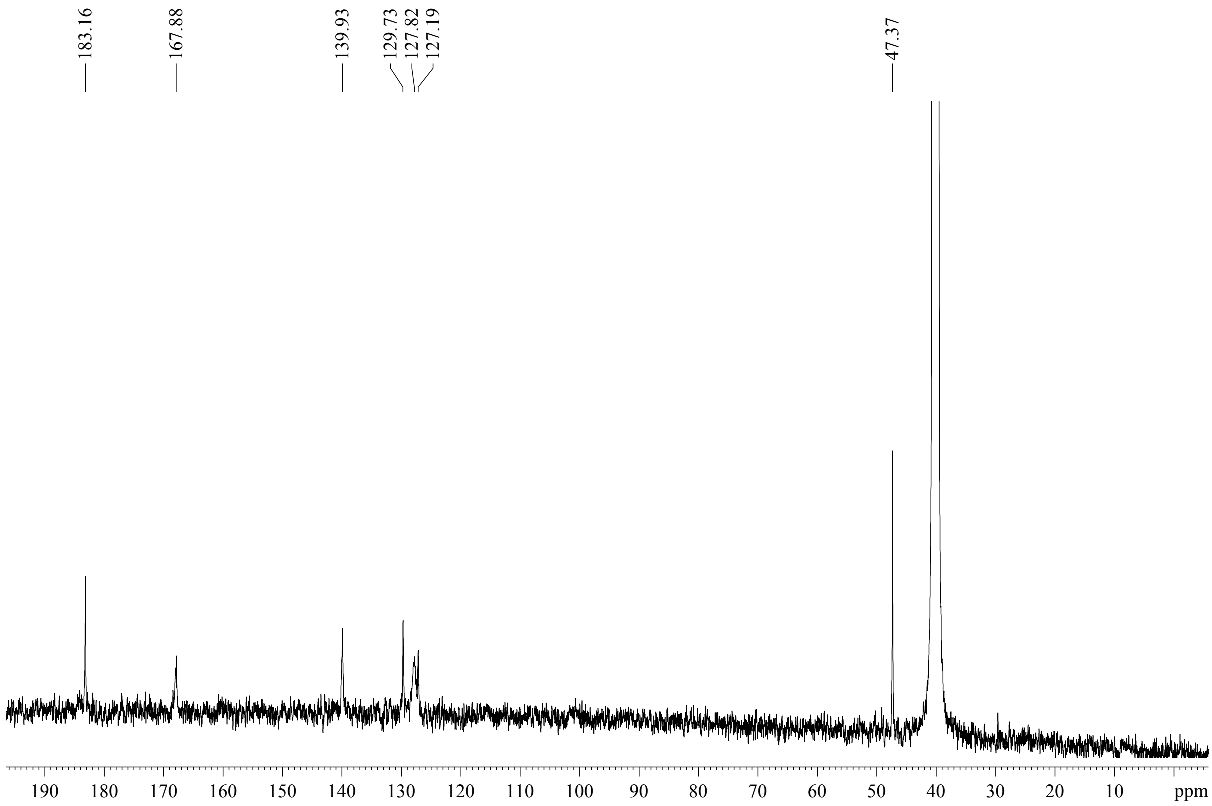

Figure S8: ^{1}H NMR (DMSO-d_6, 500 MHz) and ^{13}C NMR (DMSO-d_6, 125 MHz) spectra of **4**.

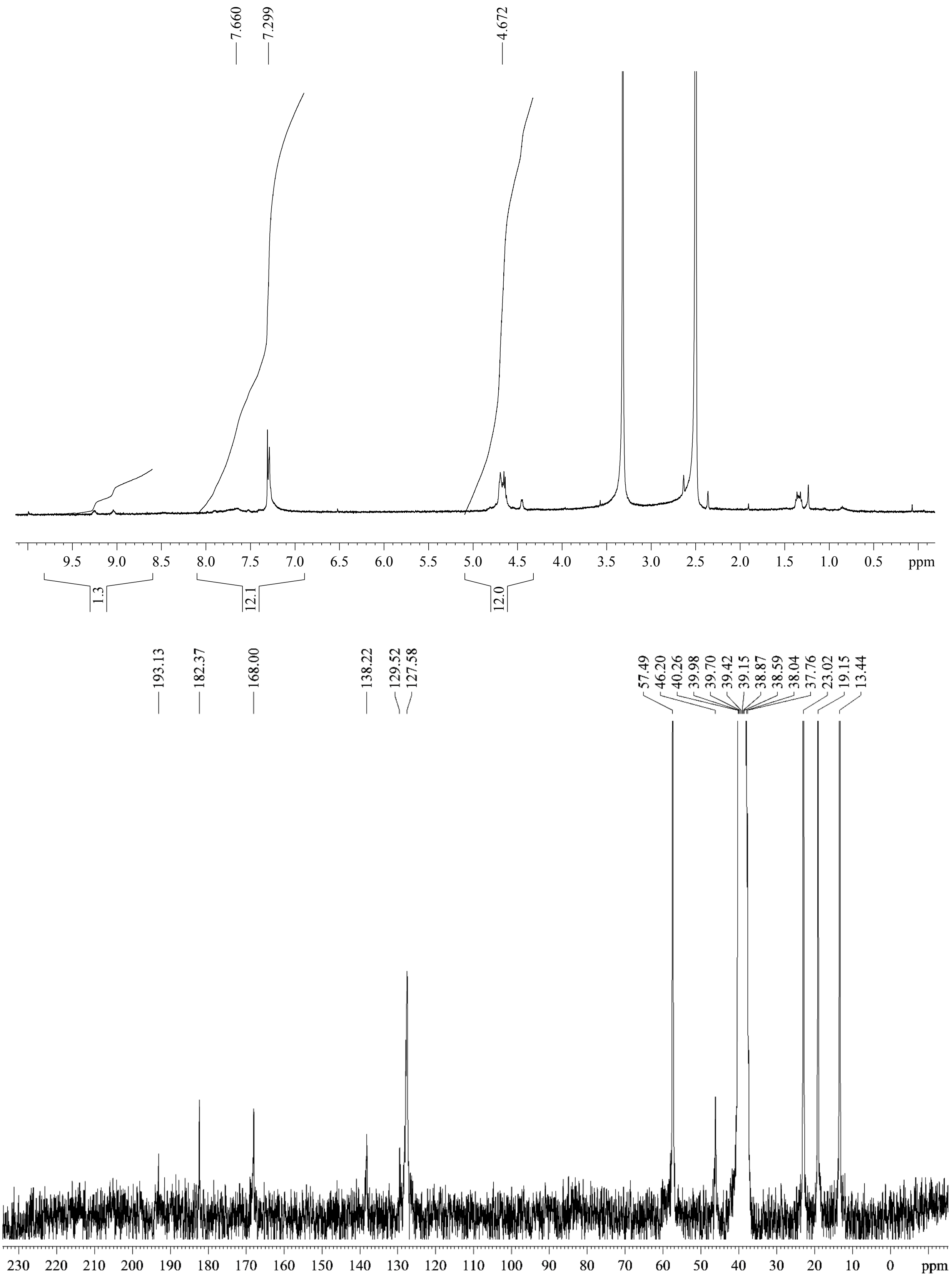

Note: This receptor's carbon NMR was run in the presecne of TBA_2SO_4 due to low solubility problem. TBA^+ peaks are at 13.44, 19.15, 23.02, 57.49.

Figure S9: ^{1}H NMR (DMSO-d_6, 500 MHz) and ^{13}C NMR (DMSO-d_6, 125 MHz) spectra of **16.**

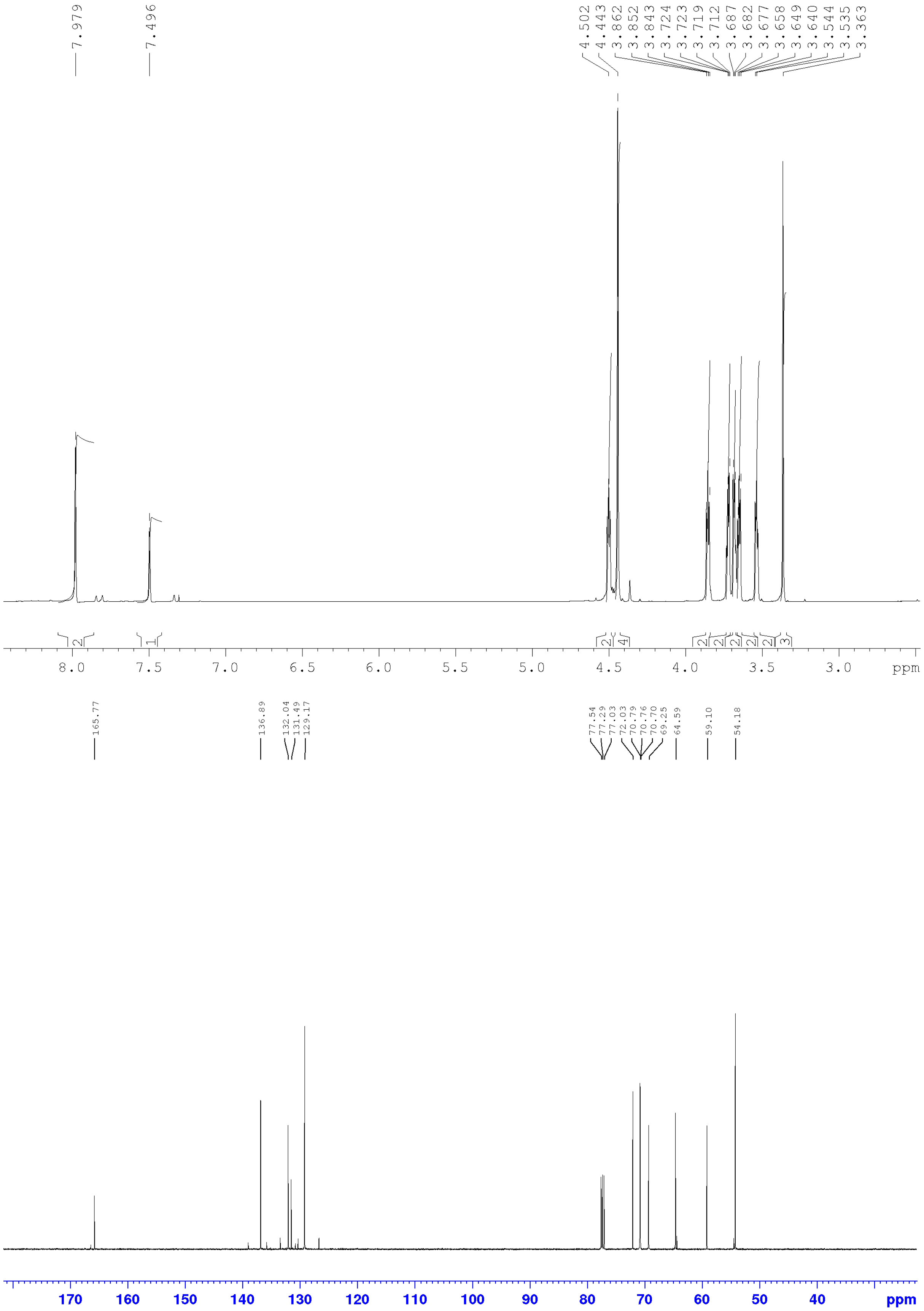

Figure S10: ^{1}H NMR ($CDCl_3$, 500 MHz) and ^{13}C NMR (D_2O, 125 MHz) spectra of **17.**

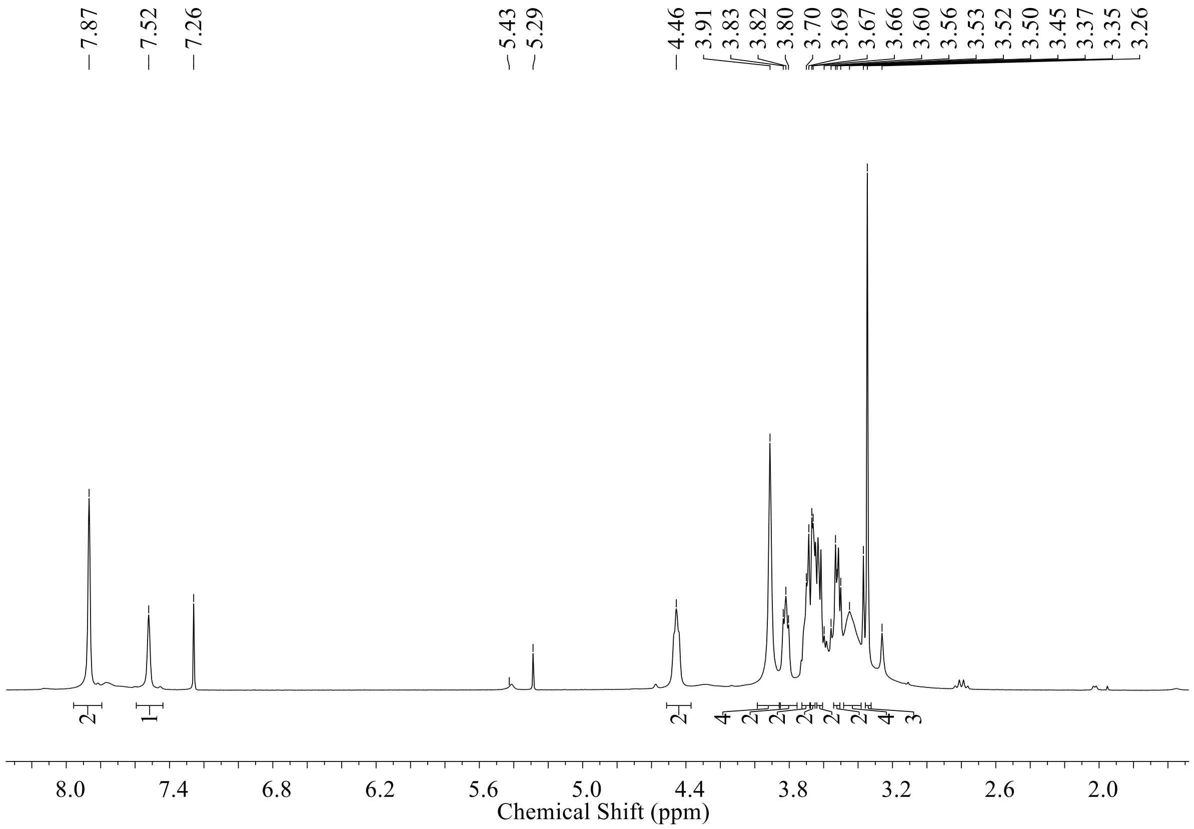

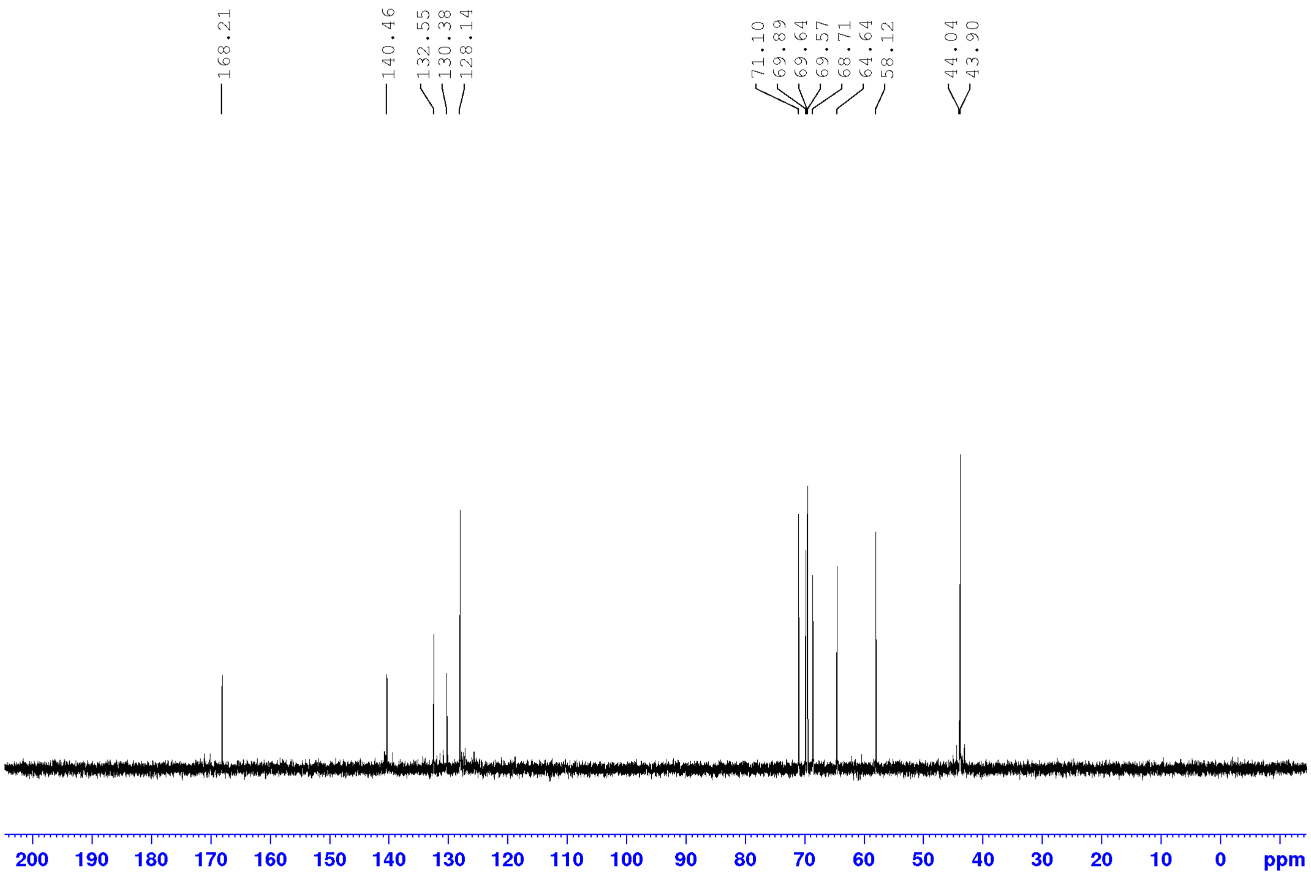

Figure S11: ^{1}H NMR (DMSO-d_6, 500 MHz) and ^{13}C NMR (DMSO-d_6, 125 MHz) spectra of **18.**

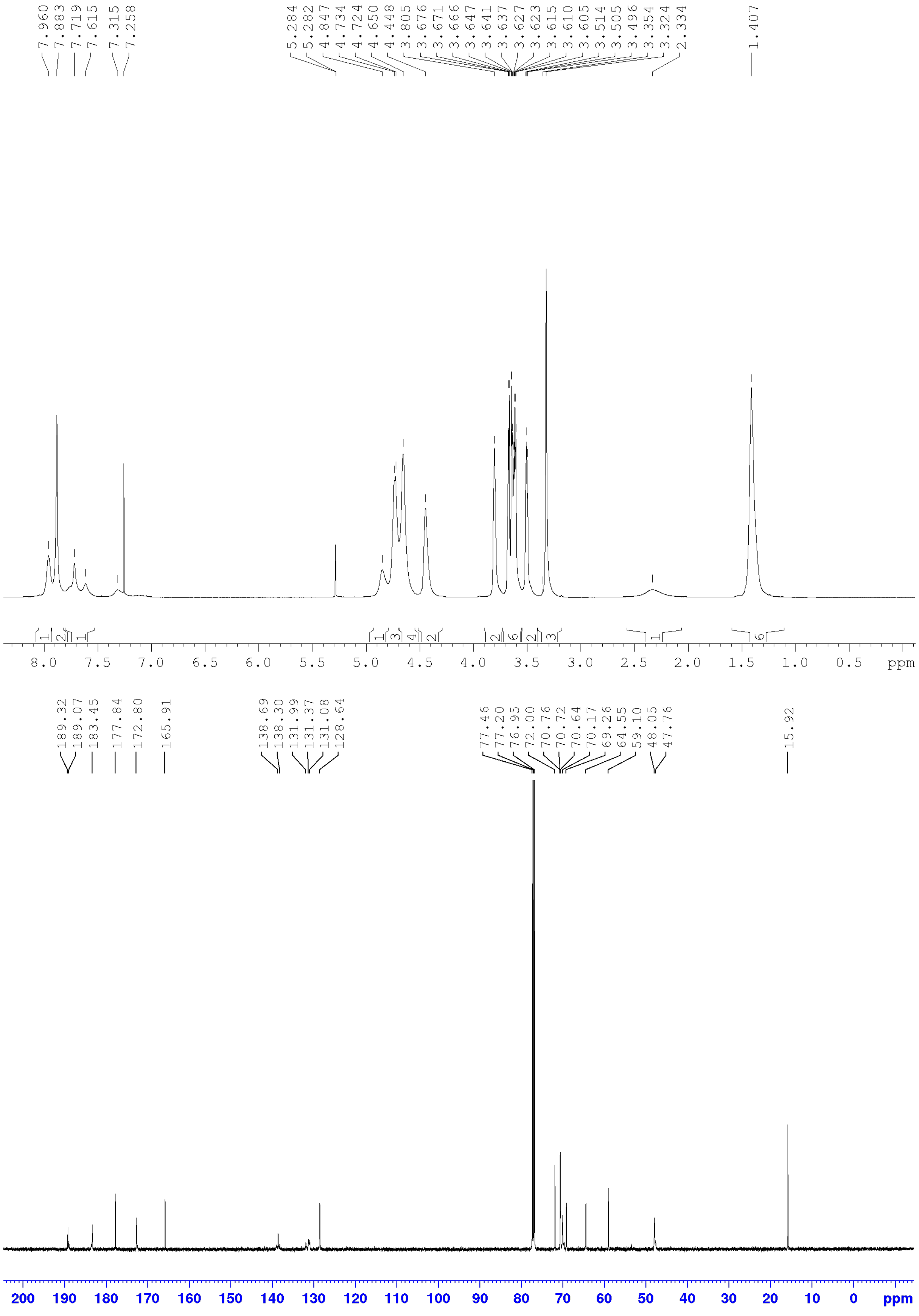

Figure S12: ^{1}H NMR (DMSO-d_6, 500 MHz) and ^{13}C NMR (DMSO-d_6, 125 MHz) spectra of **5.**

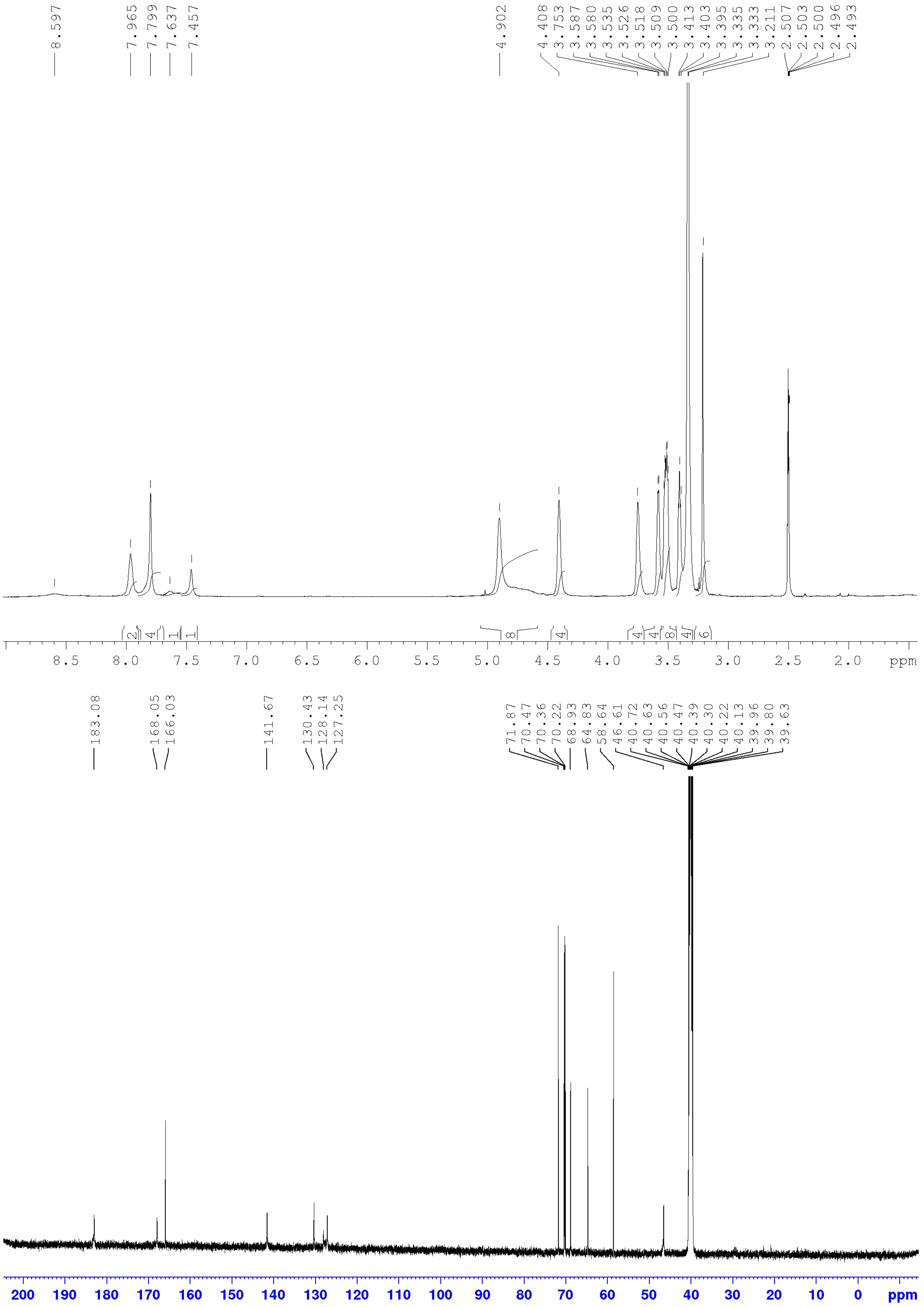

Figure S13: ^{1}H NMR ($CDCl_3$, 500 MHz) and ^{13}C NMR ($CDCl_3$, 125 MHz) spectra of **19.**

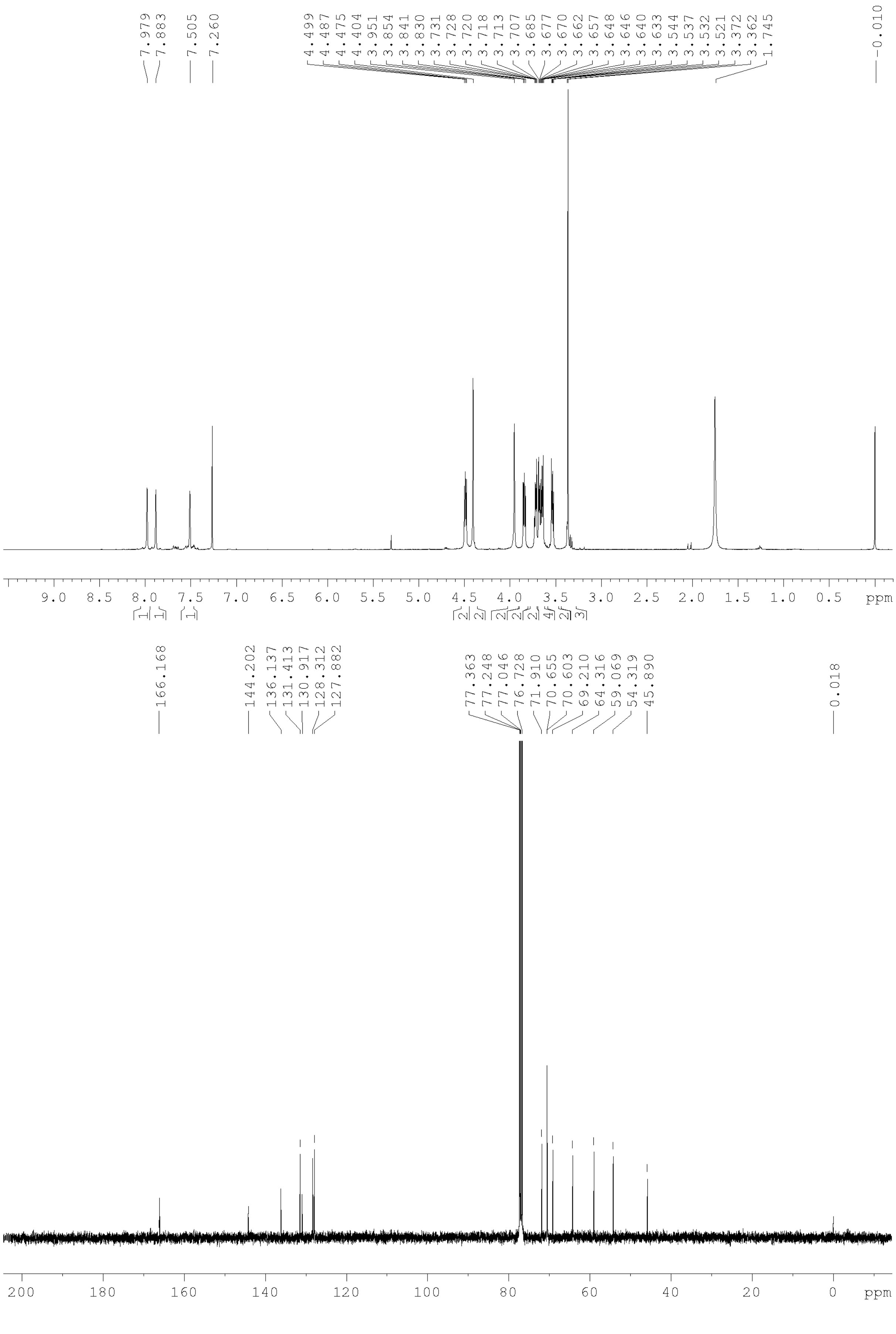

Figure S14: ^{1}H NMR ($CDCl_3$, 500 MHz) and ^{13}C NMR ($CDCl_3$, 125 MHz) spectra of **20.**

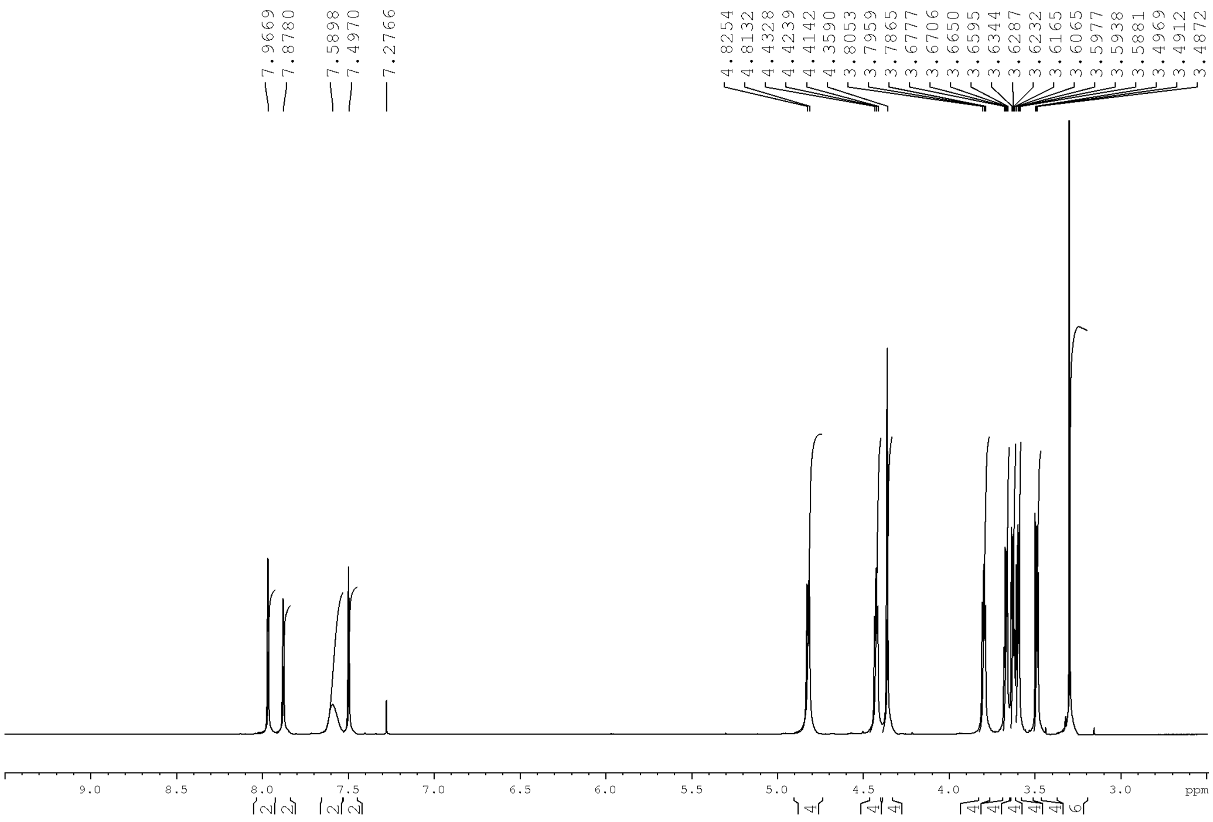

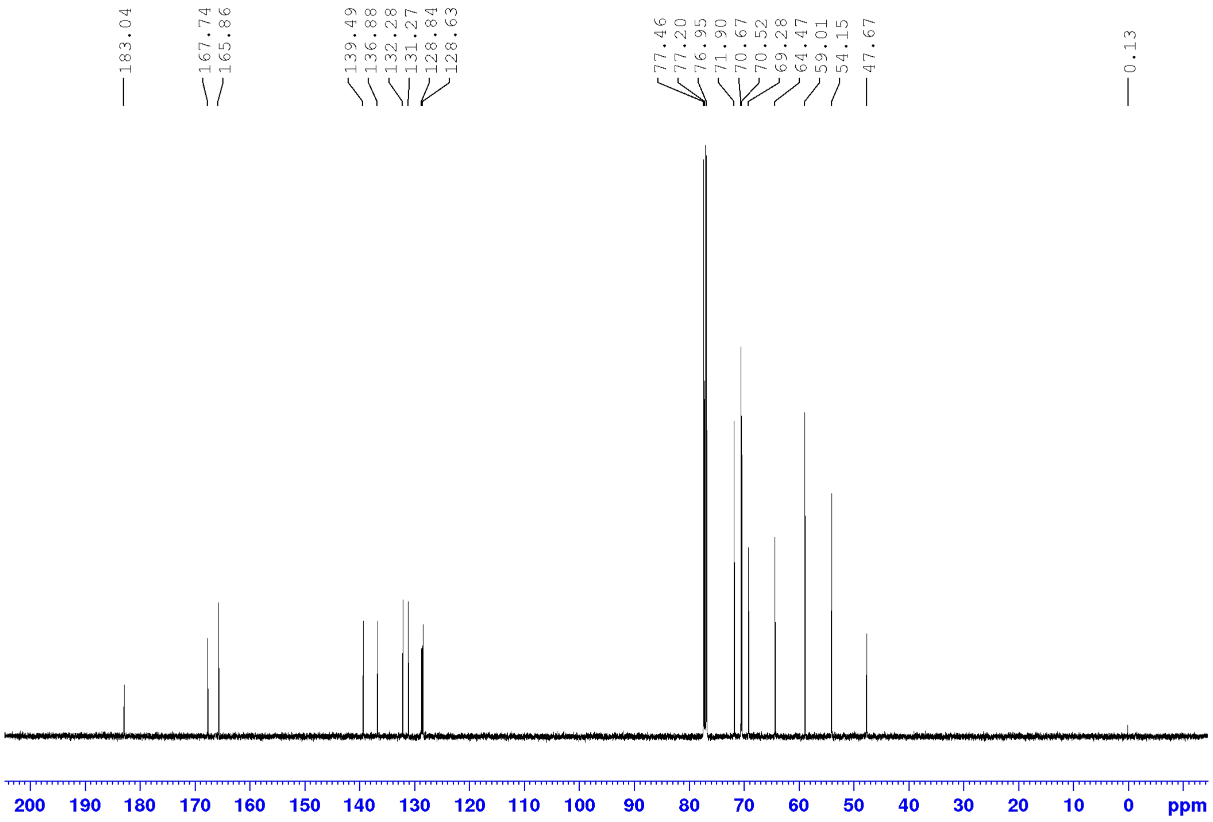

Figure S15: ^{1}H NMR (DMSO-d_6, 500 MHz) and ^{13}C NMR (DMSO-d_6, 125 MHz) spectra of **23.**

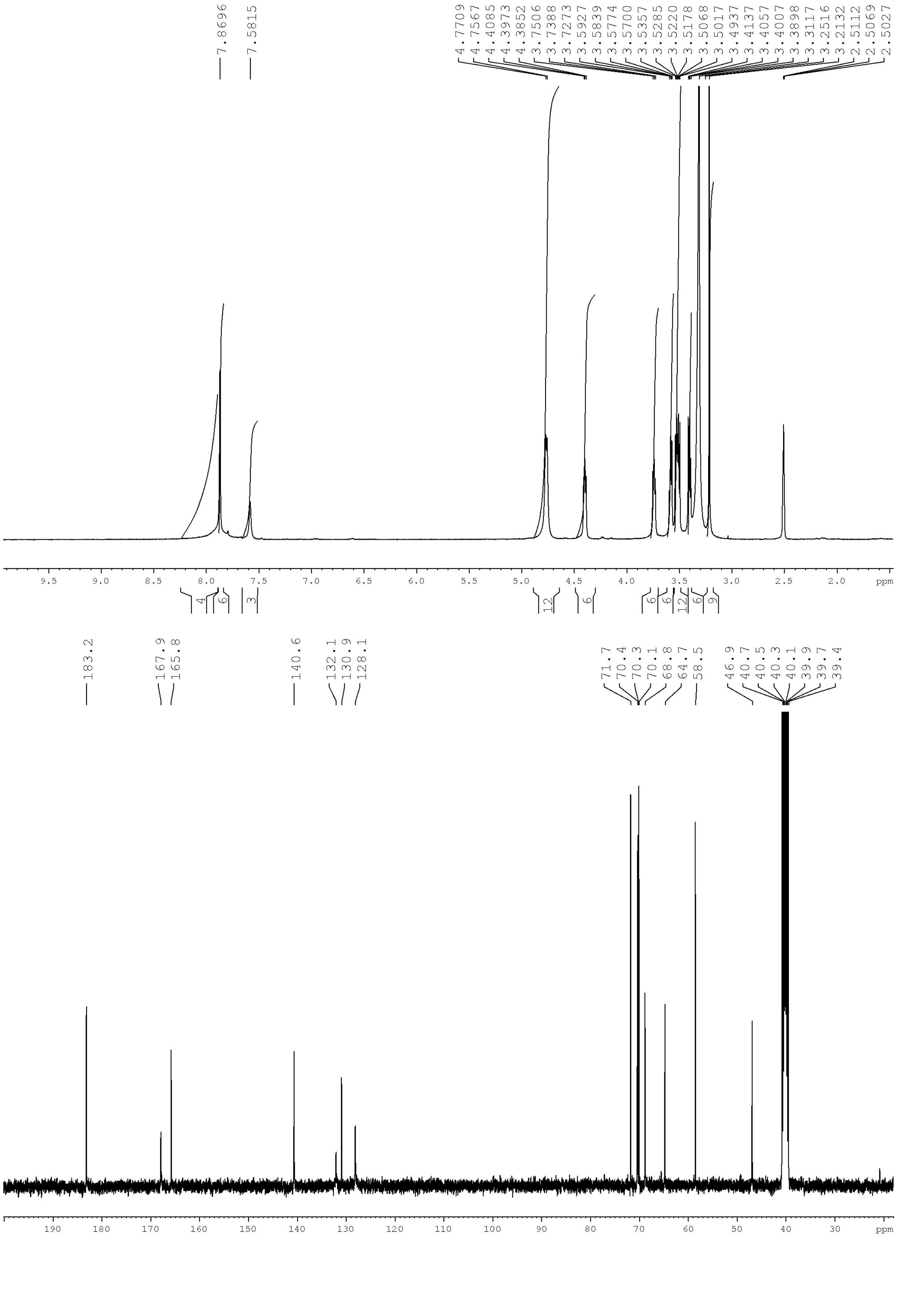

Figure S16: ^{1}H NMR ($CDCl_3$, 500 MHz), ^{13}C NMR ($CDCl_3$, 125 MHz) and ^{77}Se NMR ($CDCl_3$, 95 MHz) spectra of $(TBA)_2SeO_4$ (S1).

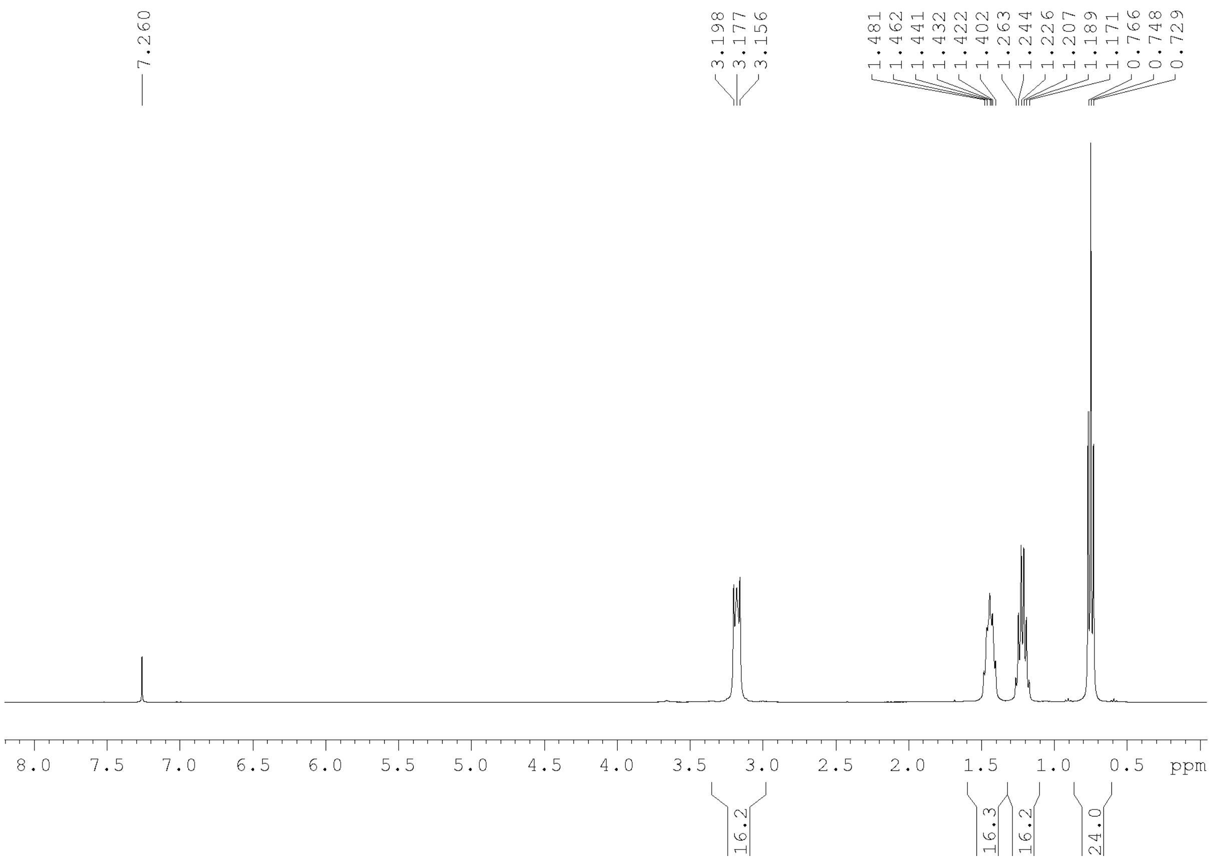

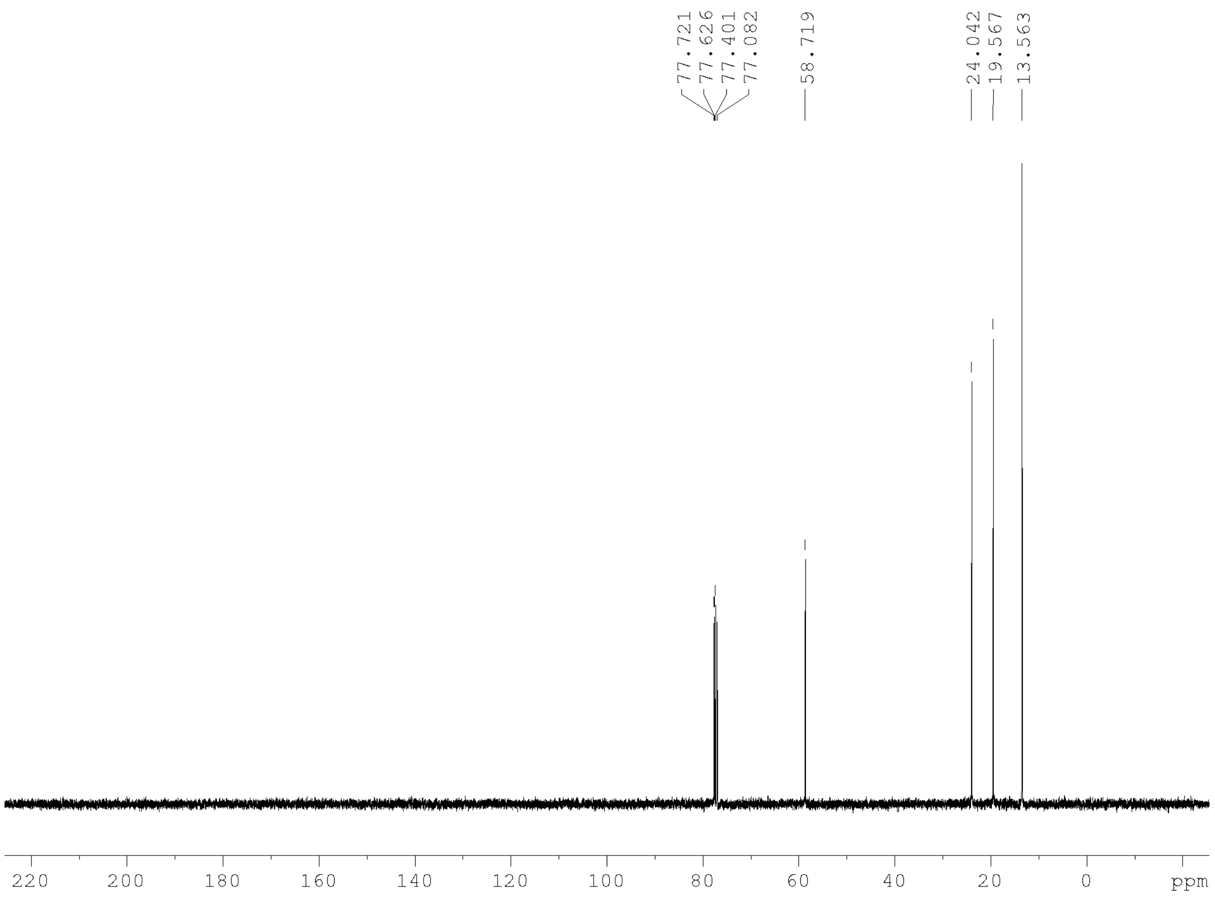

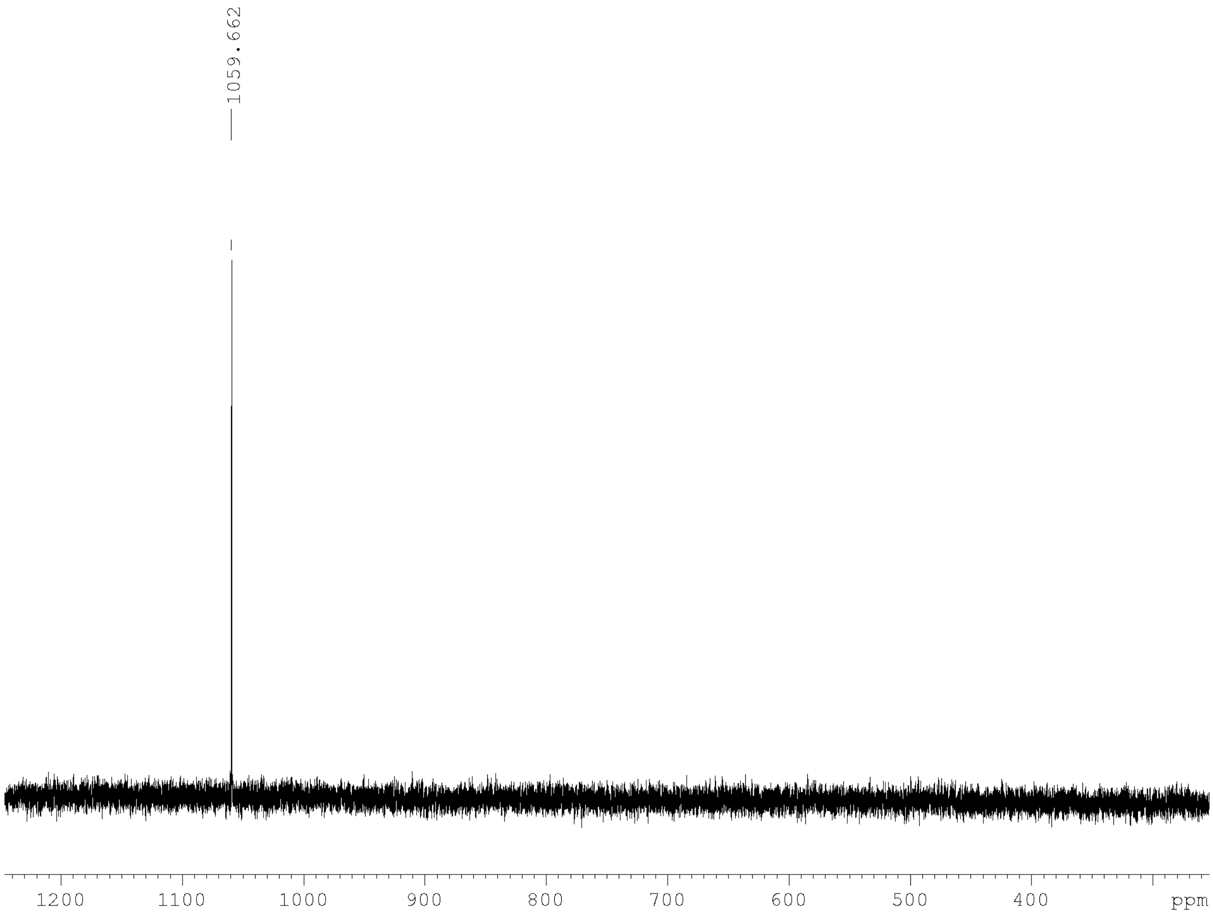
1059.662
1200
1100
1000
900
800
700
600
500
400
ppm

Figure S17: ^{1}H NMR ($CDCl_3$, 500 MHz), ^{13}C NMR ($CDCl_3$, 125 MHz) and ^{77}Se NMR ($CDCl_3$, 95 MHz) spectra of $(TBA)_2SeO_4$ (S1) with 14 eq. of diphenyl selenide as an internal standard.

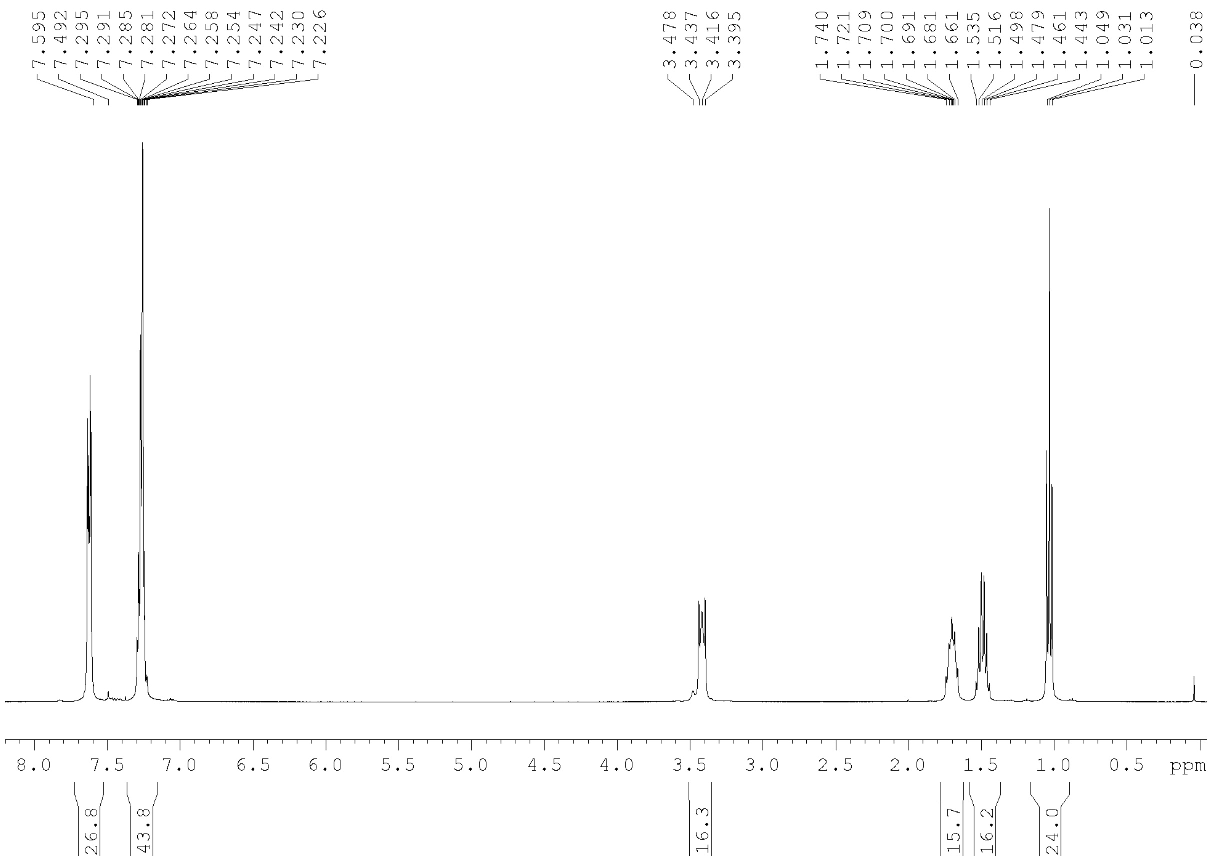

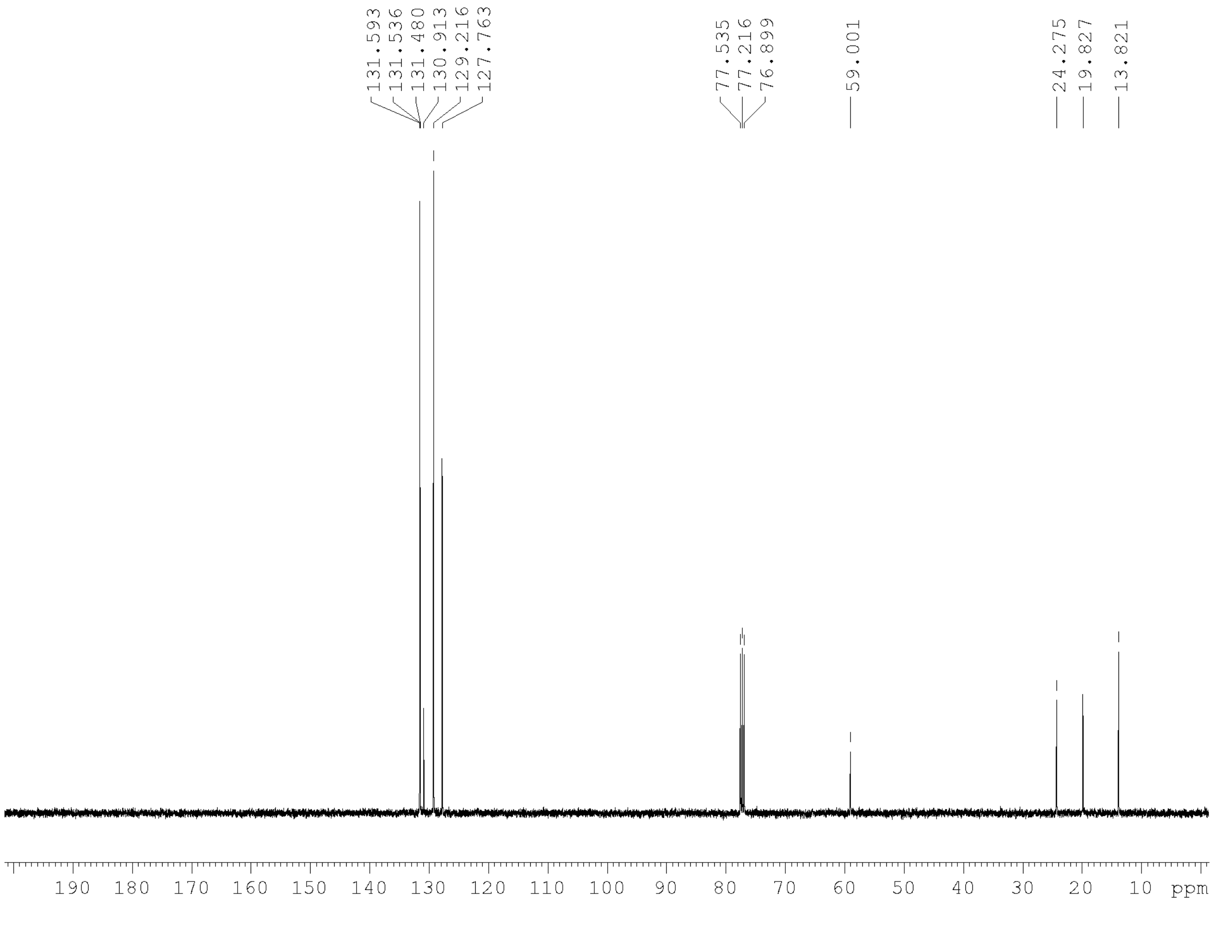

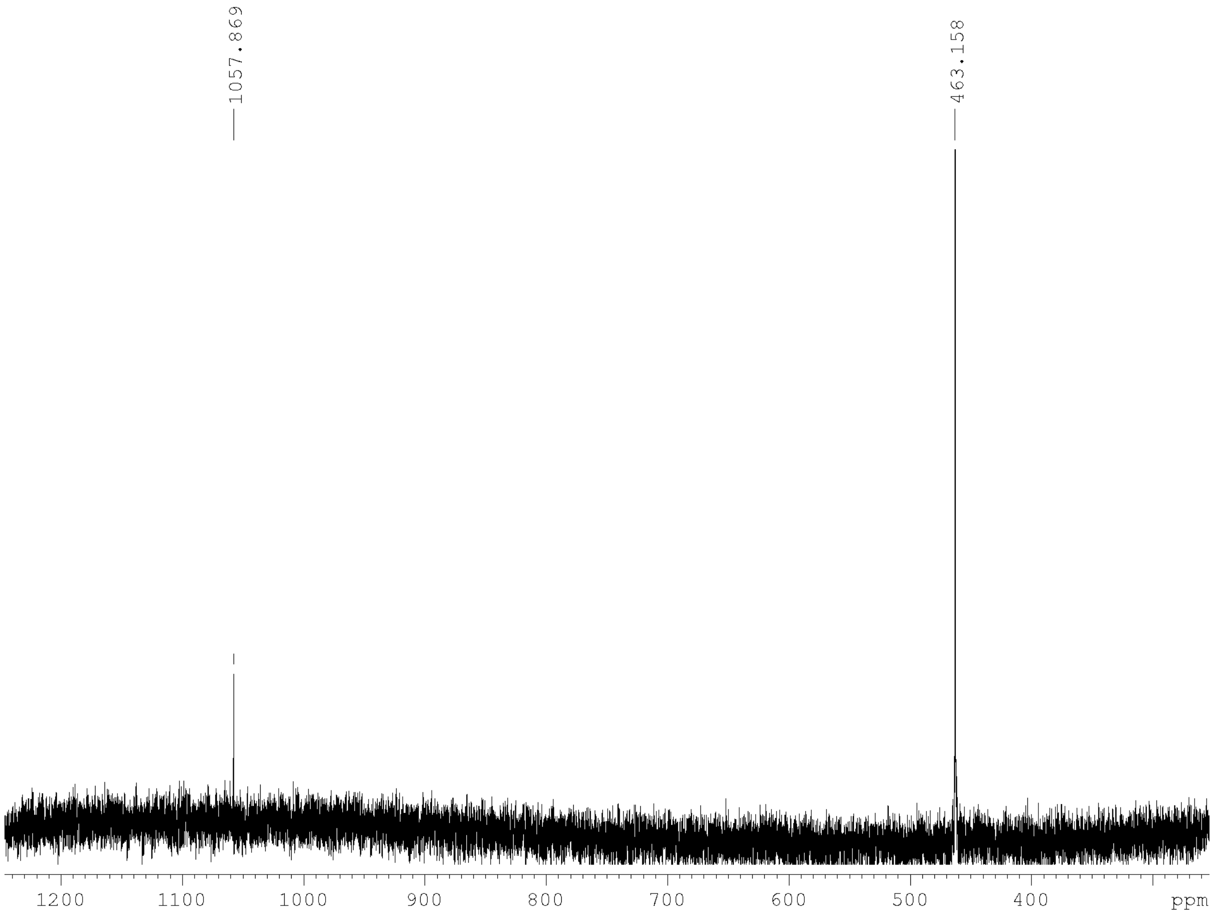
1057.869
463.158
1200
1100
1000
900
800
700
600
500
400
ppm

Figure S18: The observed (top) and calculated (bottom) HRMS isotopic distribution pattern for [**1** + SO_4^{2-} + 3 TBA^+]$^+$ in positive ESI.

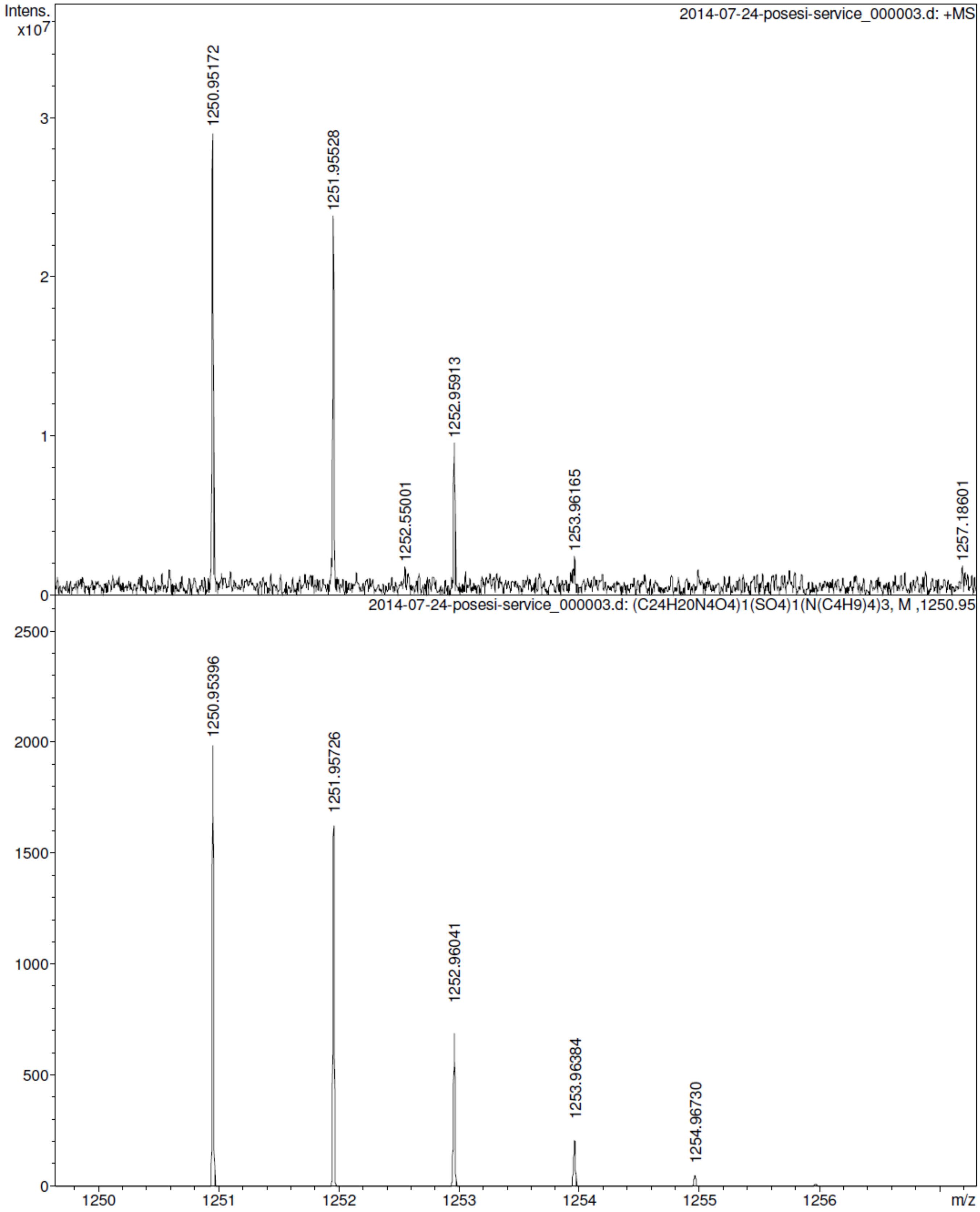

Figure S19: The observed (top) and calculated (bottom) HRMS isotopic distribution pattern for [**3** + SO_4^{2-} + 3 TBA^+]$^+$ in positive ESI.

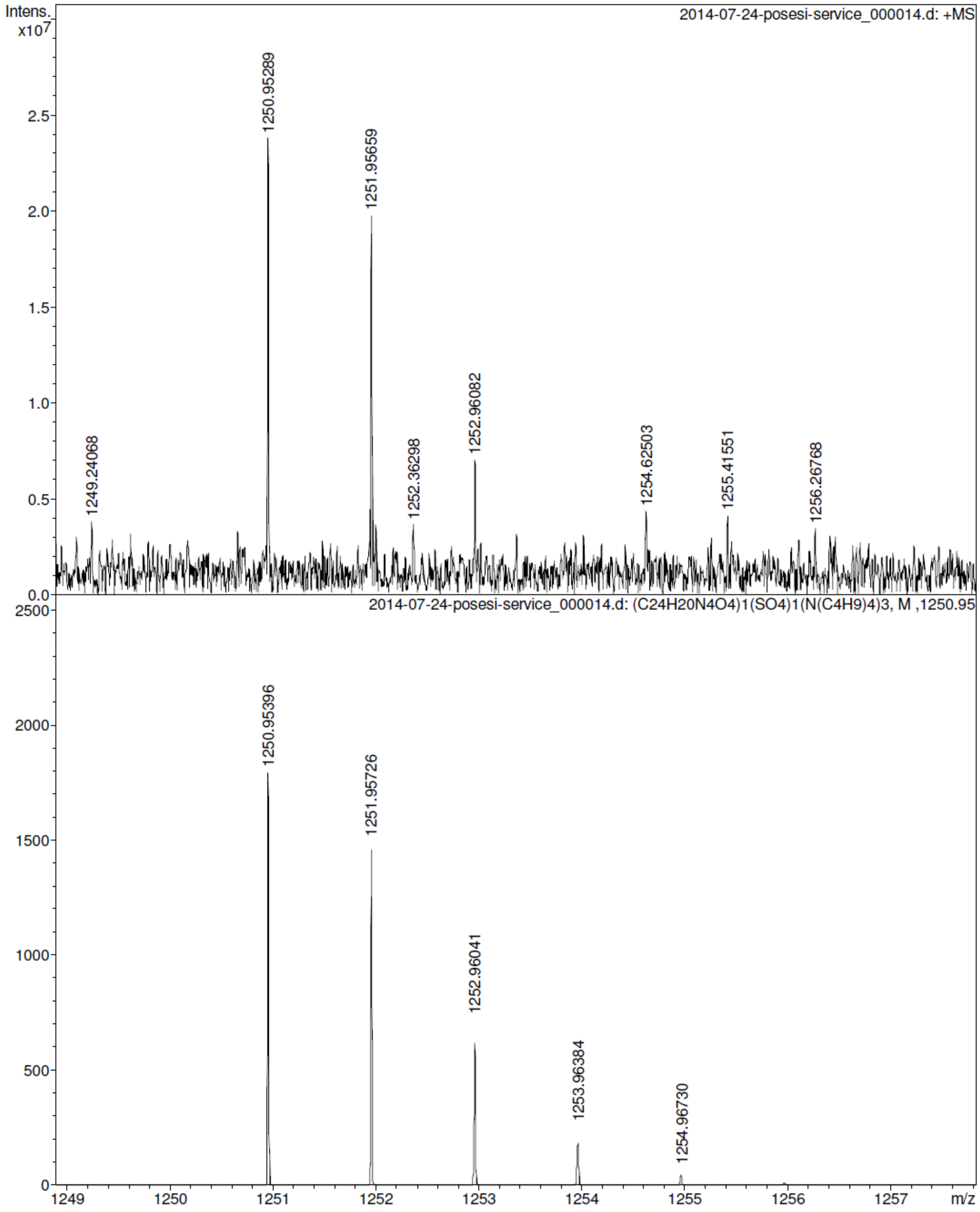

Figure S20: The observed (top) and calculated (bottom) HRMS isotopic distribution pattern for [**2** + SO_4^{2-} + 3 TBA^+]$^+$ in positive ESI.

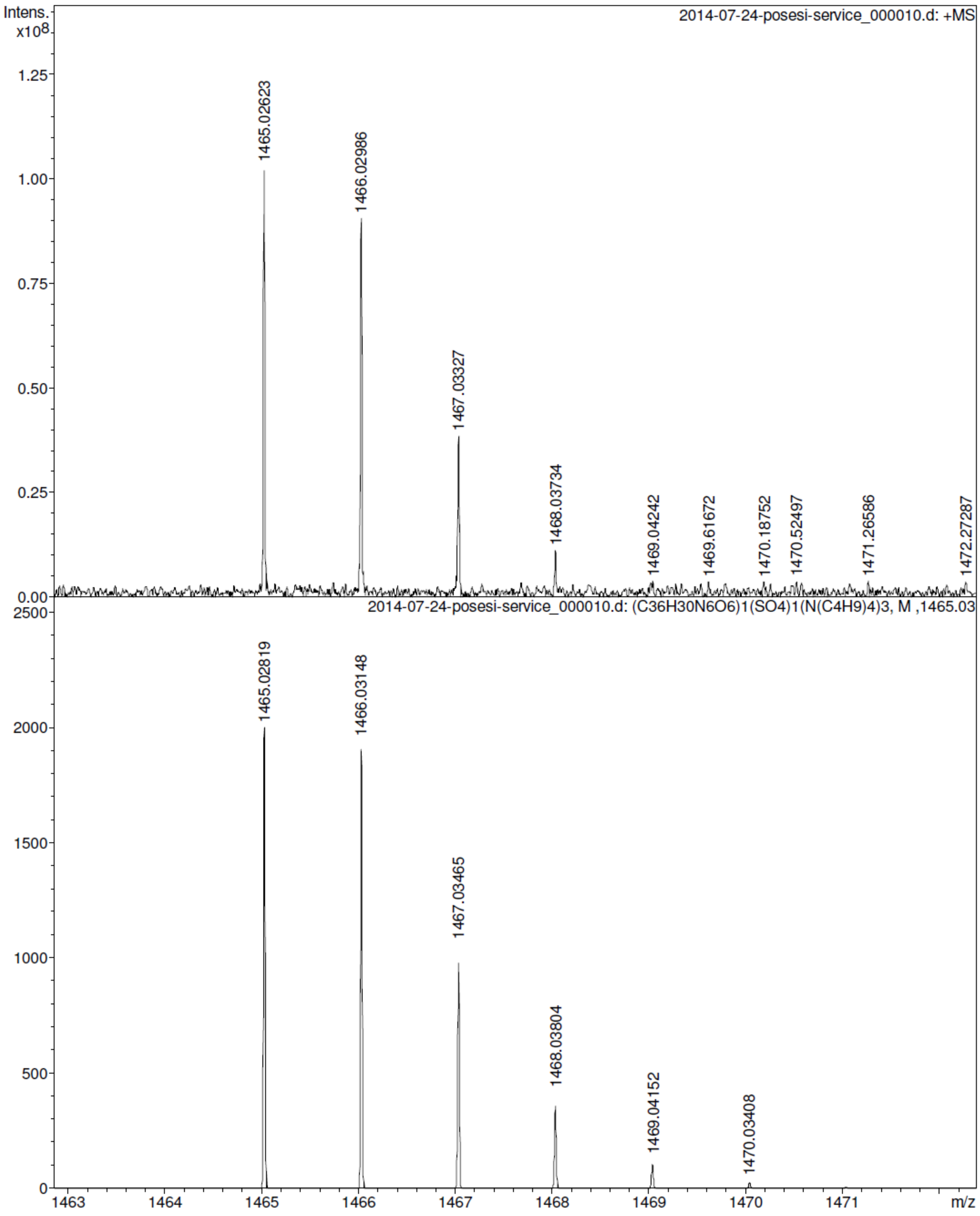

Figure S21: The observed (top) and calculated (bottom) HRMS isotopic distribution pattern for [**2** – 3H + 4 TBA$^+$]$^+$ in positive ESI.

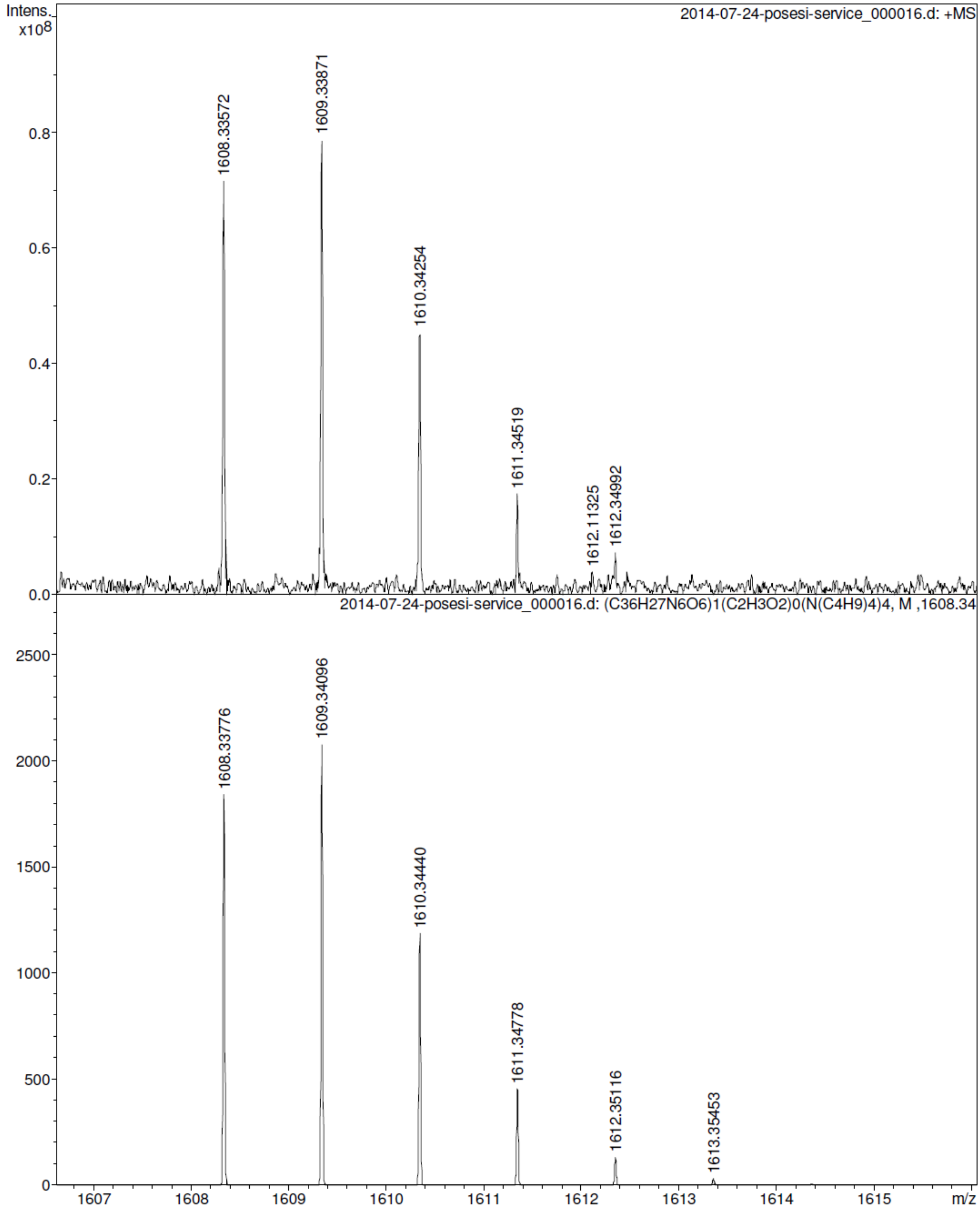

^{1}H NMR Binding Studies of Novel Receptors

Procedure: A 2.5 mM stock solution of receptor was accurately prepared in the stated deuterated solvents (v/v) using a volumetric flask. Solutions of anions to be titrated were prepared in separate 5 ml vials and 2000μL deuterated solvents (v/v) were added using pipettes (Eppendorf). The concentration of anion solutions were made 70 times that of the host (i.e. 160 – 180 mM). In each case, 550 μL of host solution in an NMR tube was titrated with aliquots of anion stock solution and after each addition, and the ^{1}H NMR spectrum was recorded on a Bruker Avance III 500 spectrometer after thorough mixing in 300K. Typically this was performed in the following order: 10 × 1.5 μL, 2×7.5 μL, 4× 14 μL (total 86 μL). Titrations were performed in triplicate to give K_a values. Typically a total of at least 12 equiv. of anion was added. Non-linear curve fitting of the experimentally obtained titration isotherms (equivalents of anion versus chemical shift of NH, aromatic CH and methylene CH protons) using the program HypNMR (Hyperquad) enabled the calculation of association constants (K_a/M^{-1}) using a 1:1 global fits model.

Figure S22: ^{1}H NMR Titrations of receptor 1: (a): Anion screening. (b): ^{1}H NMR titration of **1** with $(TBA)_2SO_4$ (0 -12 eq.) in DMSO-d_6 at 300 K. (c): ^{1}H NMR titration of **1** with TBAAcO (0 -12 eq.) in DMSO-d_6 at 300 K. (d): ^{1}H NMR titration of **1** with $TBAH_2PO_4$ (0 -12 eq.) in DMSO-d_6 at 300 K. (e): ^{1}H NMR titration of **1** with TBACl (0 -12 eq.) in DMSO-d_6 at 300 K. (f): ^{1}H NMR titration of **1** with $TBAHSO_4$ (0 -12 eq.) in DMSO-d_6 at 300 K.

(a): Anion screening.

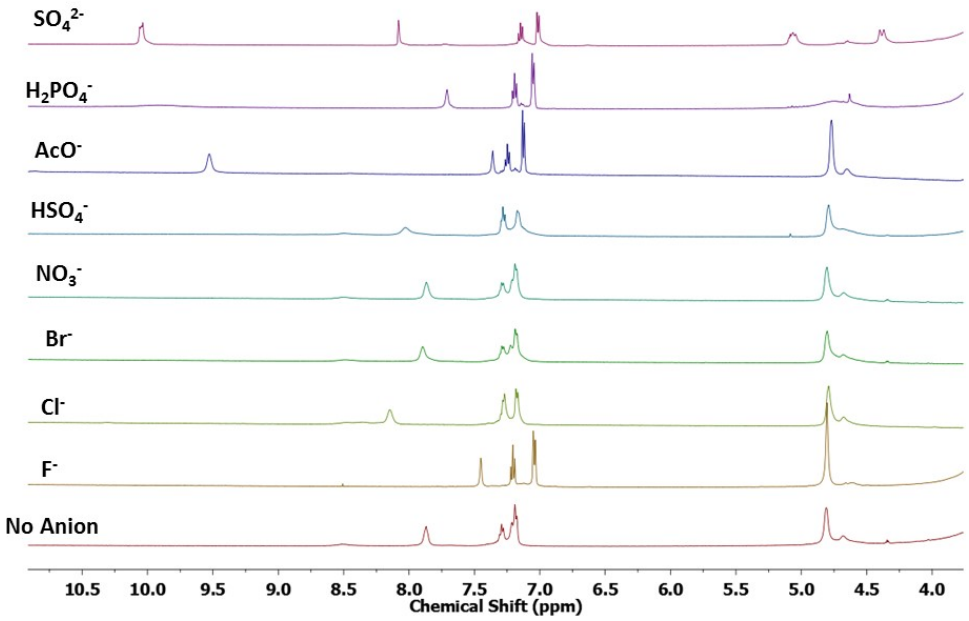

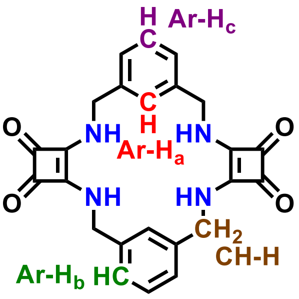

The structure of receptor **1**.

(b): ^{1}H NMR titration of **1** with TBA_2SO_4 (0 -12 eq.) in DMSO-d_6 at 300 K.

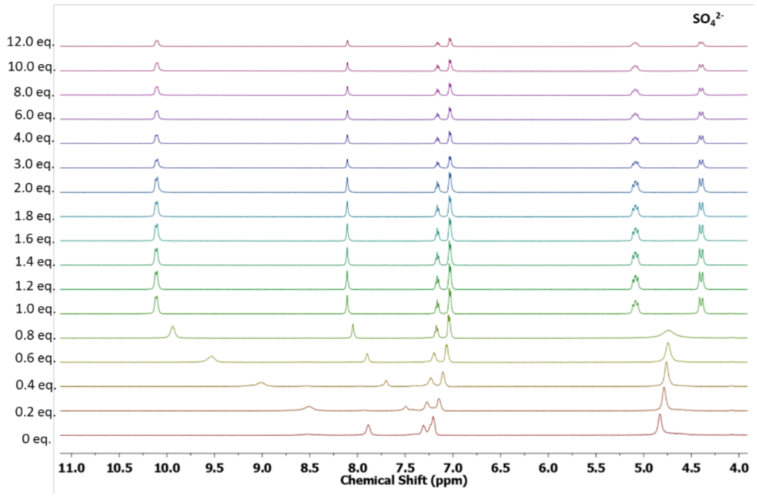

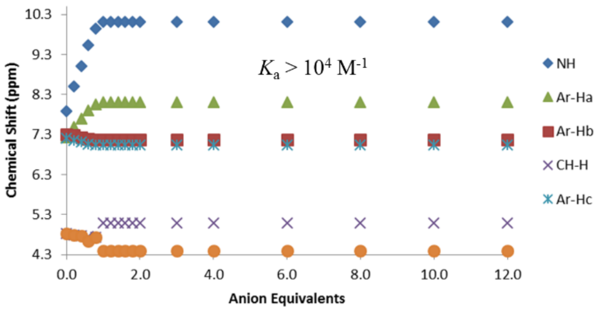

NMR stackplot (left) and changes observed in squaramide NH, aromatic protons, and methylene proton (right).

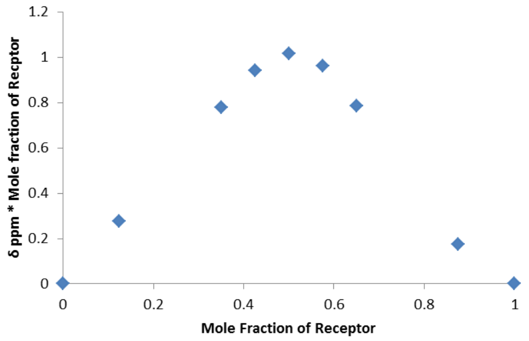

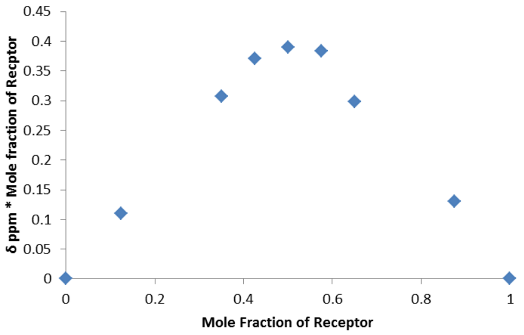

Job's Plot evaluated by squaramide NH proton(left) and aromatic CH proton (right).

(c): ^{1}H NMR titration of **1** with TBAAcO (0 -12 eq.) in DMSO-d_6 at 300 K.

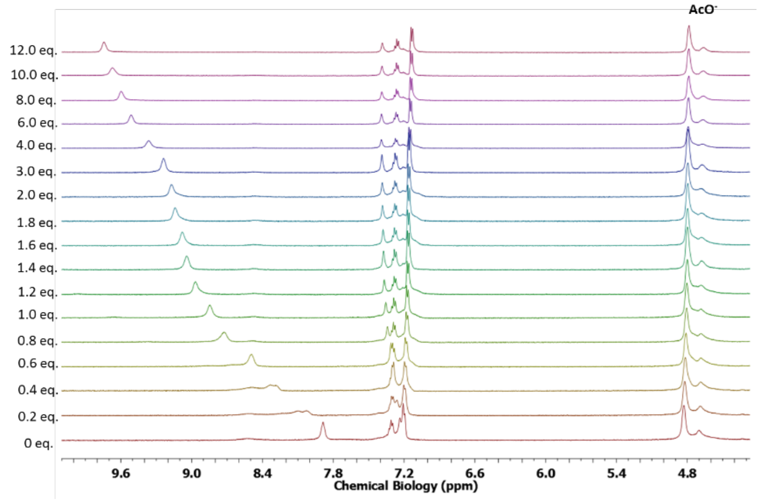

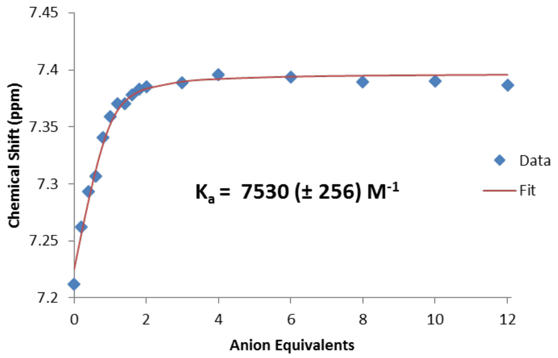

NMR stackplot (left) and fitplot by squaramide NH (right).

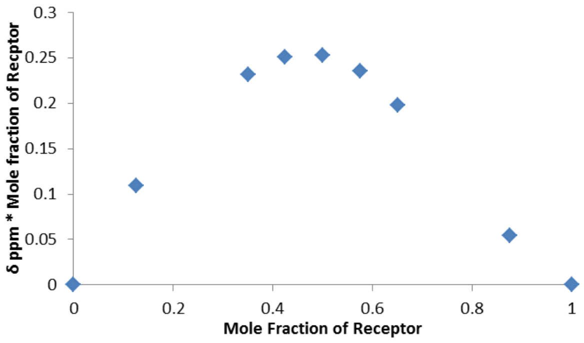

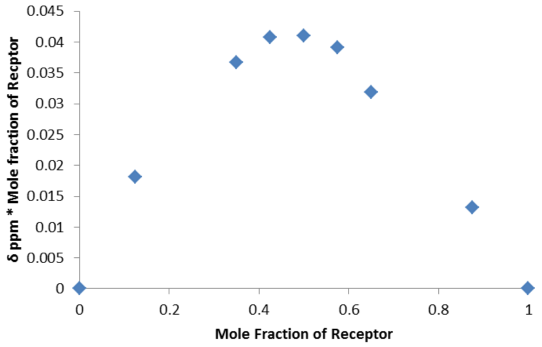

Job's Plot evaluated by squaramide NH proton (left) and aromatic CH proton (right).

Note: An accurate binding constant could not be obtained using the shift of the NH proton signal; a reliable constant was obtained by using the shift of the aromatic proton.

(d): ^{1}H NMR titration of **1** with $TBAH_2PO_4$ (0 -12 eq.) in DMSO-d_6 at 300 K.

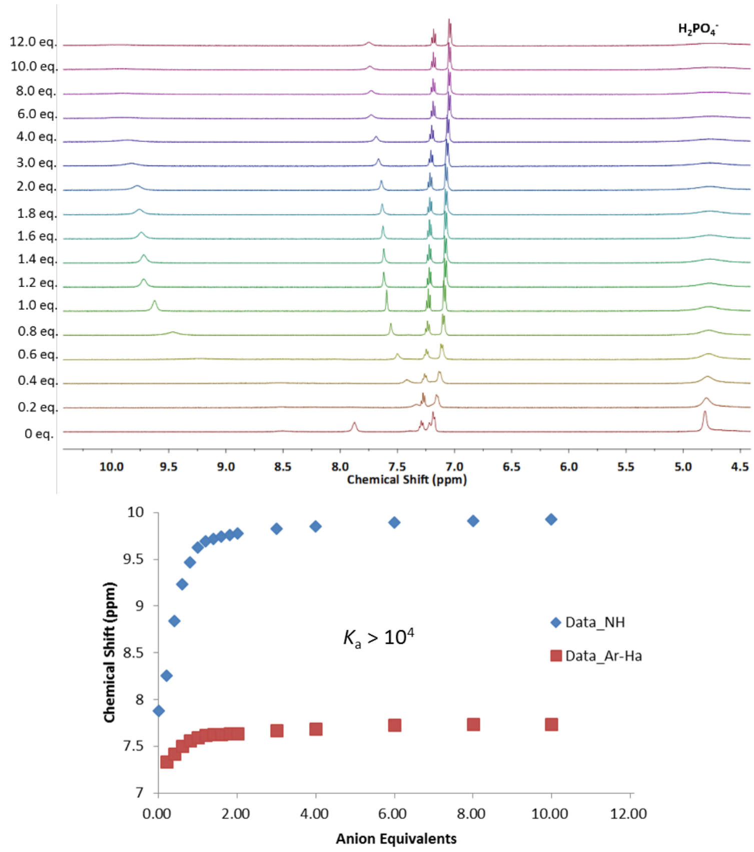

NMR stackplot (left) and changes observed in squaramide NH and aromatic proton (right).

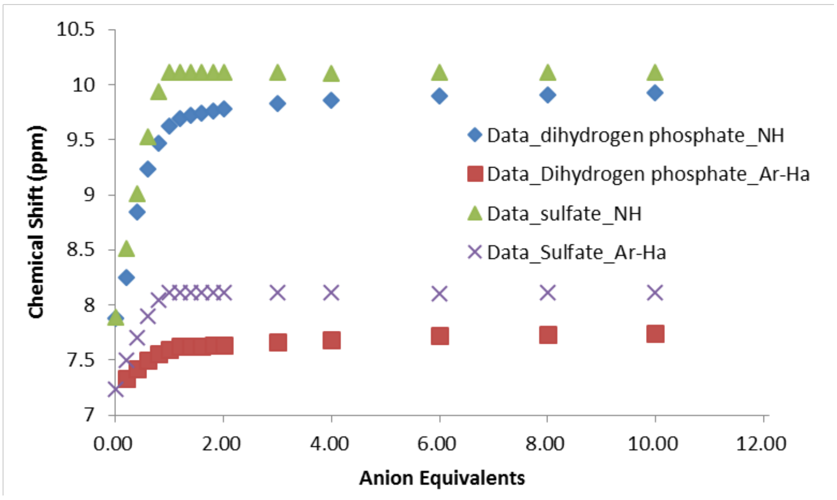

Note: Due to the strong binding of receptor **1** to both sulfate and dihydrogen phosphate (all constants higher that 10^4 M^{-1}), an accurate binding constant cannot be calculated. However, a qualitative assement based on the slope sof the binding isotherms (above) of **1** with each of these two anions, indicates that receptor **1** binds to sulfate with higher affinity than it does to dihydrogen phosphate.

(e):^{1}H NMR titration of **1** with TBACl (0 -12 eq.) in DMSO-d_6 at 300 K.

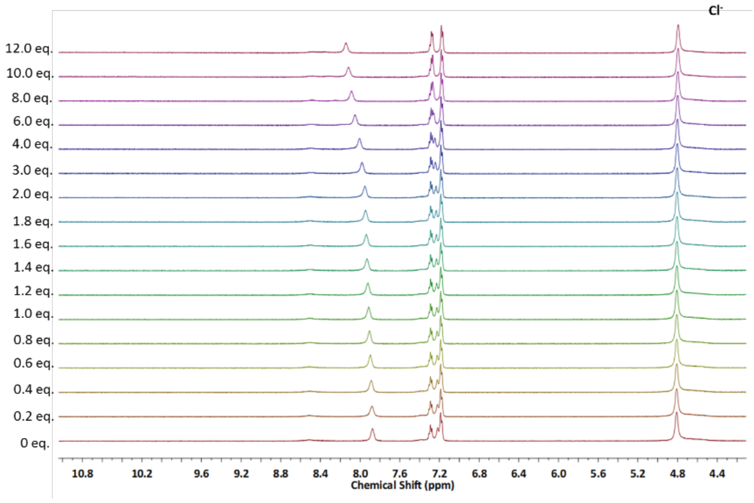

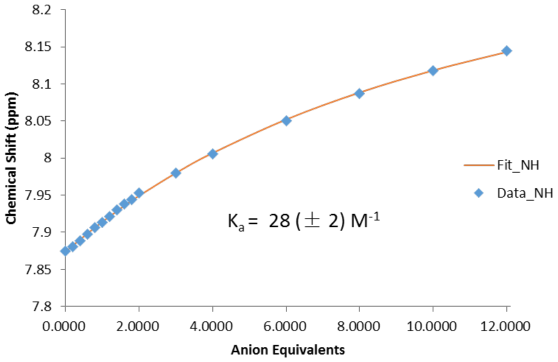

NMR stackplot (left) and fitplot (right) for squaramide NH proton.

(f): ^{1}H NMR titration of **1** with TBAHSO$_4$ (0 -12 eq.) in DMSO-d_6 at 300 K.

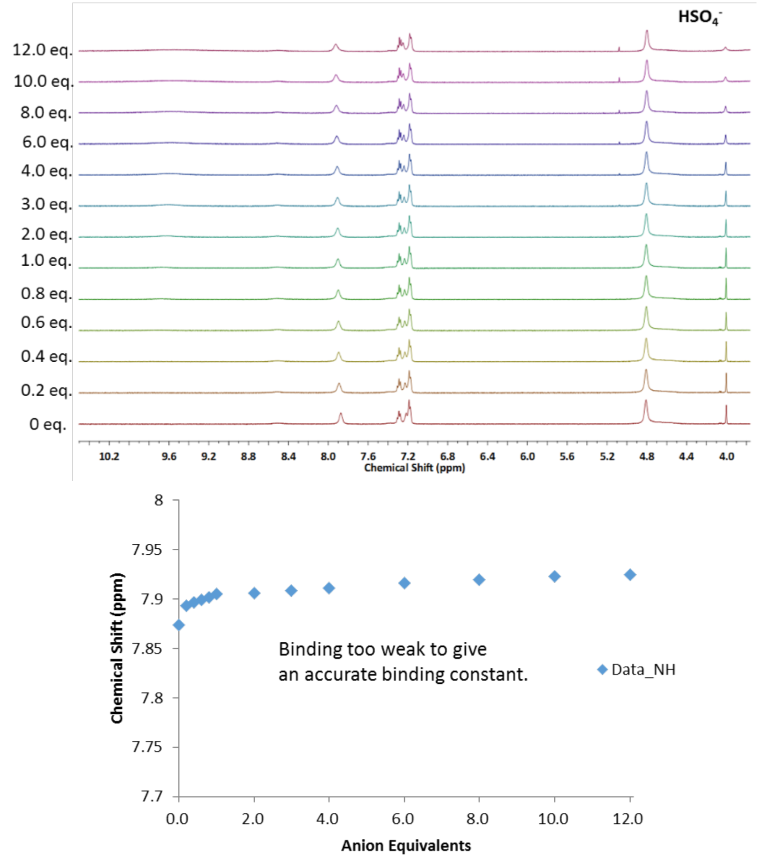

NMR stackplot (left) and fitplot (right) for squaramide NH proton (Binding too weak to determine an accurate binding constant.).

Figure S23: ^{1}H NMR Titrations of receptor **3** in DMSO-d_6 at 300 K**:** (a): Anion screening. (b): with $(TBA)_2SO_4$ (0 -12 eq.). (c): with TBAAcO (0 -12 eq.).

(a): Anion screening.

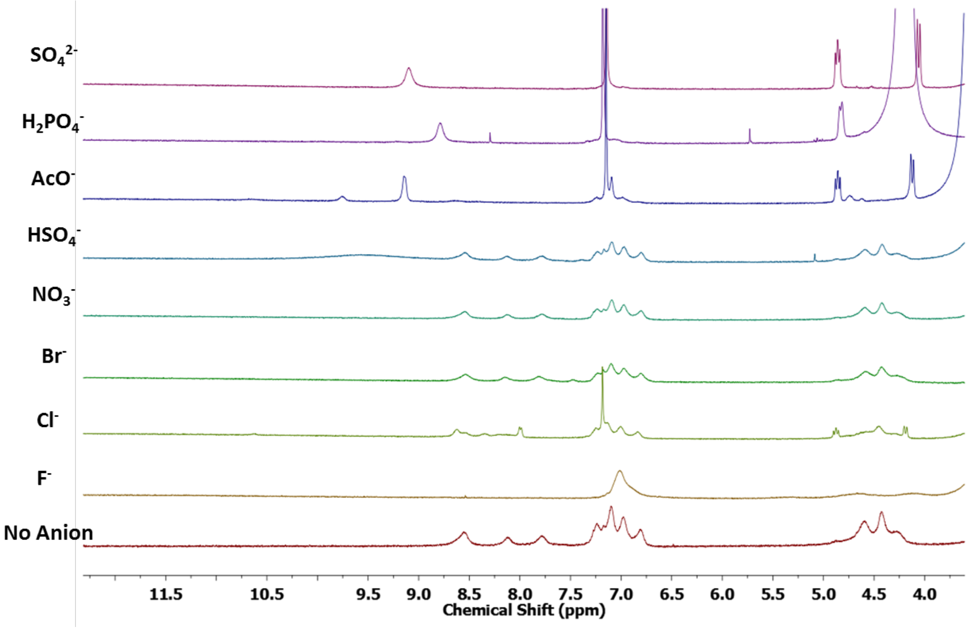

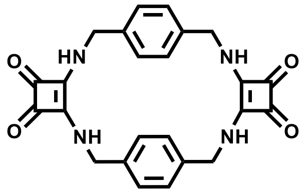

The structure of receptor **3**.

(b): ^{1}H NMR titration of **3** with $(TBA)_2SO_4$ (0 -12 eq.) in DMSO-d_6 at 300 K.

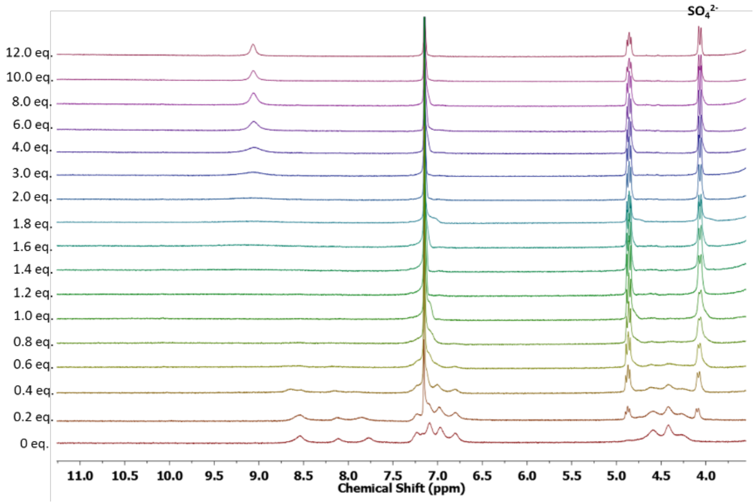

Note: Due to slow exchange of conformers on the NMR time scale at low sulfate concentrations, shifts of signals can not be readily followed to allow binding constants to be determined.

(c): ^{1}H NMR titration of **3** with TBAAcO (0 -12 eq.) in DMSO-d_6 at 300 K.

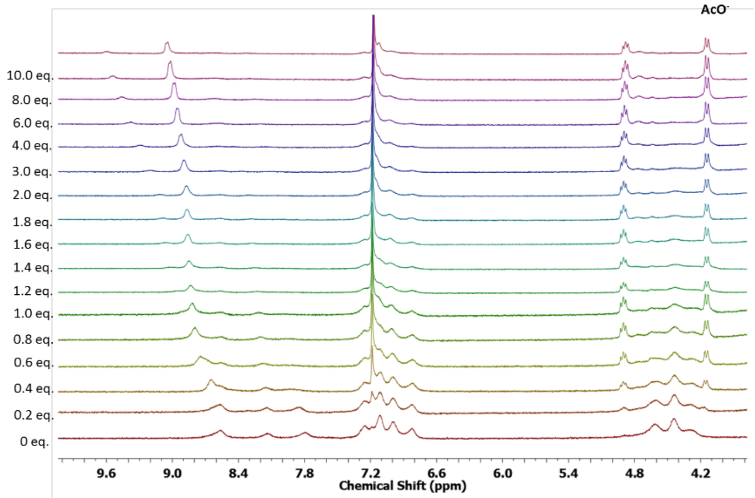

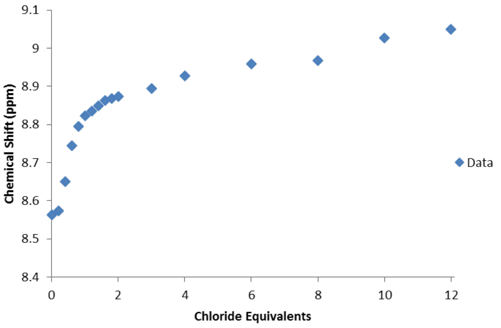

Fitplot for squaramide NH proton.

Note: Data could not be fit to a reasonable binding model.

Figure S24: ^{1}H NMR Titrations of receptor 2: (a): Anion screening. (b): ^{1}H NMR titration of **2** with $(TBA)_2SO_4$ (0 -12 eq.) in DMSO-d_6 at 300 K. (c): ^{1}H NMR titration of **2** with TBAAcO (0 -12 eq.) in DMSO-d_6 at 300 K. (d): ^{1}H NMR titration of **2** with $TBAH_2PO_4$ (0 -12 eq.) in DMSO-d_6 at 300 K. (e): ^{1}H NMR titration of **2** with TBACl (0 -12 eq.) in DMSO-d_6 at 300 K.

(a): Anion screening.

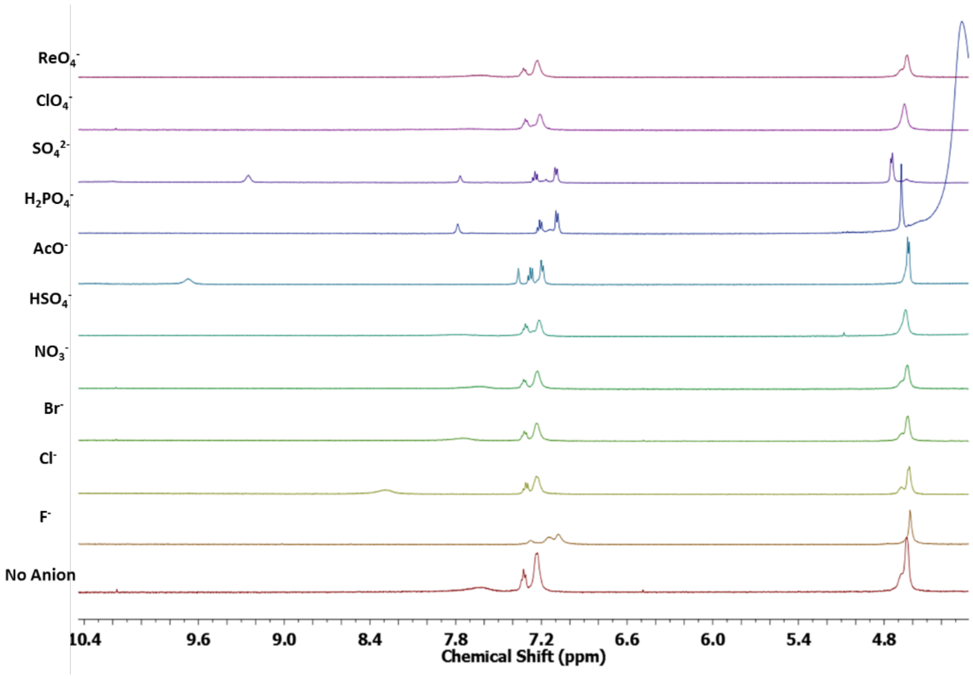

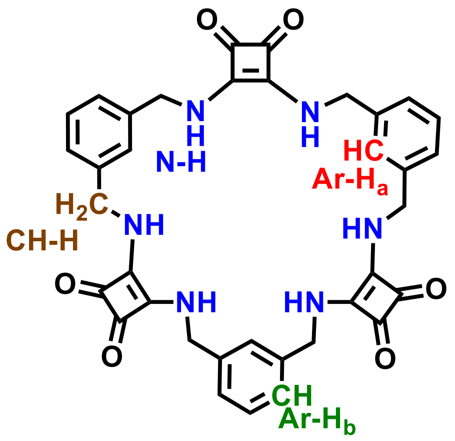

The structure of receptor **2**.

(b): ^{1}H NMR titration of **2** with $(TBA)_2SO_4$ (0 -12 eq.) in DMSO-d_6 at 300 K.

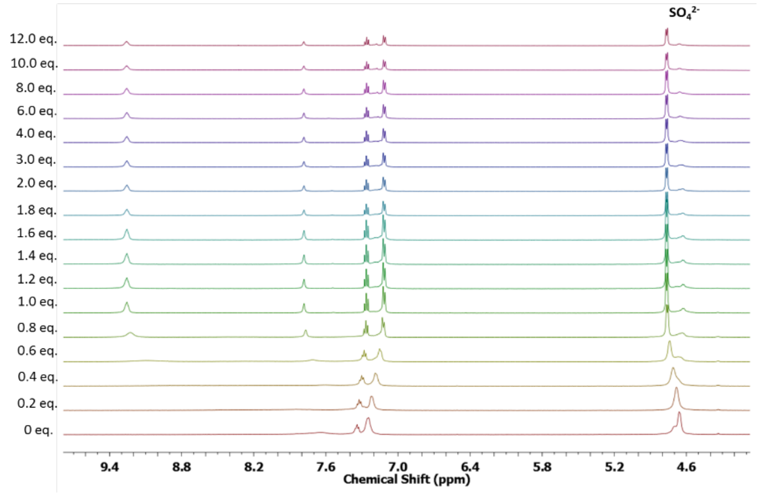

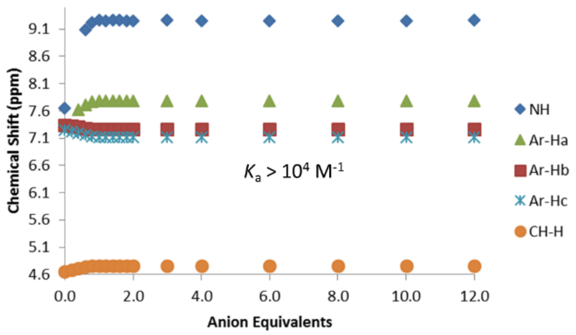

NMR stackplot (left) and fitplot (right) for squaramide NH, aromatic CH and methylene proton.

Note: $K_a > 10^4$. An accurate binding constant cannot be calculated due to the strong binding.

(c): ^{1}H NMR titration of **2** with TBAAcO (0 -12 eq.) in DMSO-d_6 at 300 K.

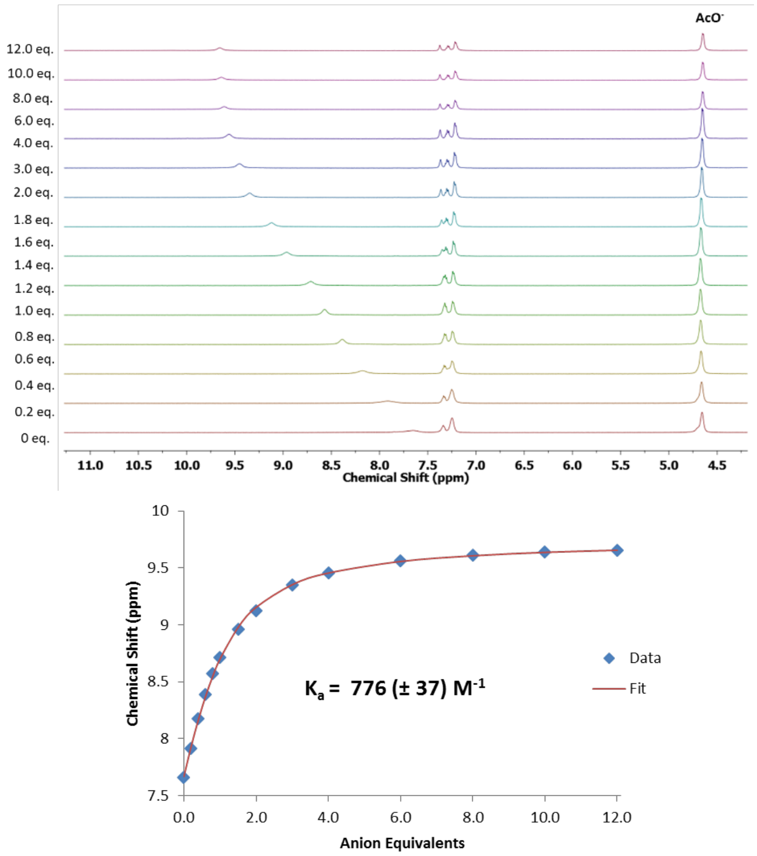

NMR stackplot (left) and fitplot (right) for squaramide NH proton.

(d): ^{1}H NMR titration of **2** with $TBAH_2PO_4$ (0 -12 eq.) in DMSO-d_6 at 300 K.

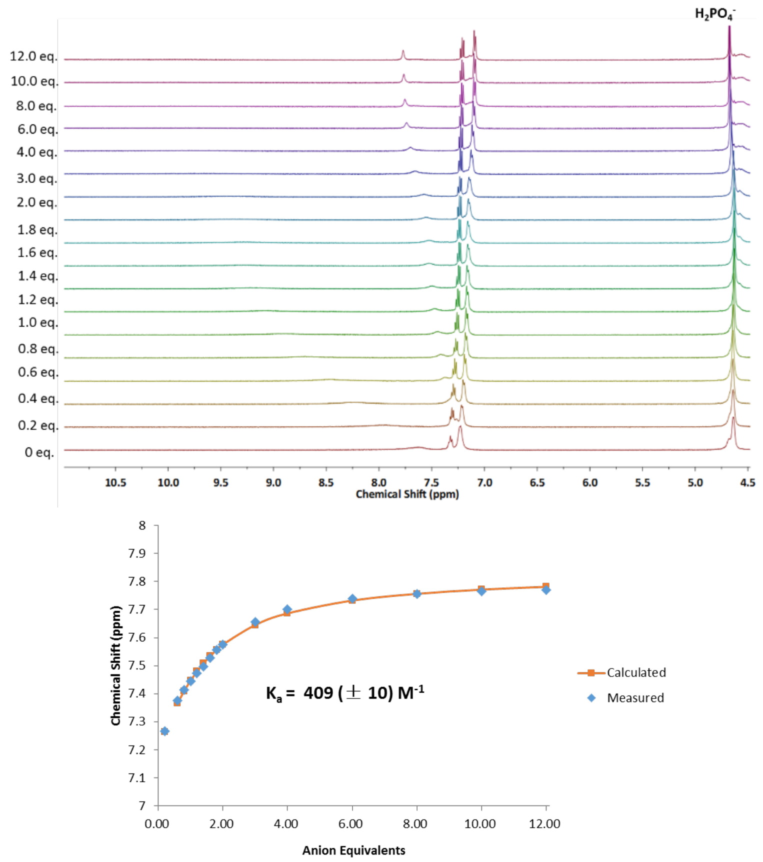

NMR stackplot (left) and fitplot (right) for squaramide aromatic proton.

(e): ^{1}H NMR titration of **2** with TBACl (0 -12 eq.) in DMSO-d_6 at 300 K.

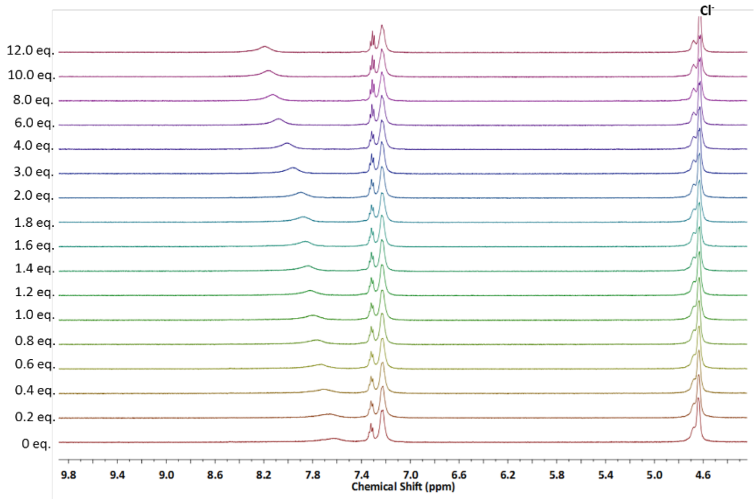

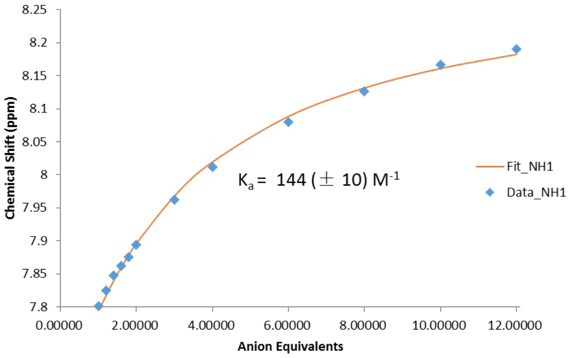

NMR stackplot (left) and fitplot for squaramide NH proton(right).

Figure S25: ^{1}H NMR Titrations of receptor **4** in DMSO-d_6 with $(TBA)_2SO_4$ (0 -12 eq.)at 300 K.

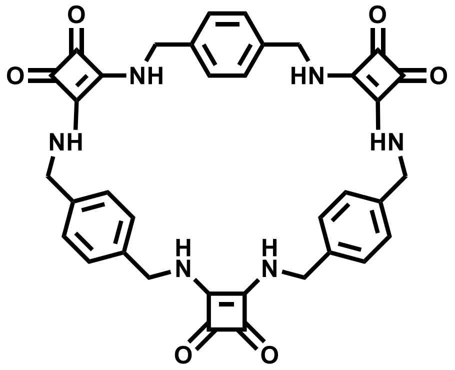

The structure of receptor **4**.

(a): ^{1}H NMR titration of **4** with $(TBA)_2SO_4$ (0 -12 eq.) in DMSO-d_6 at 300 K.

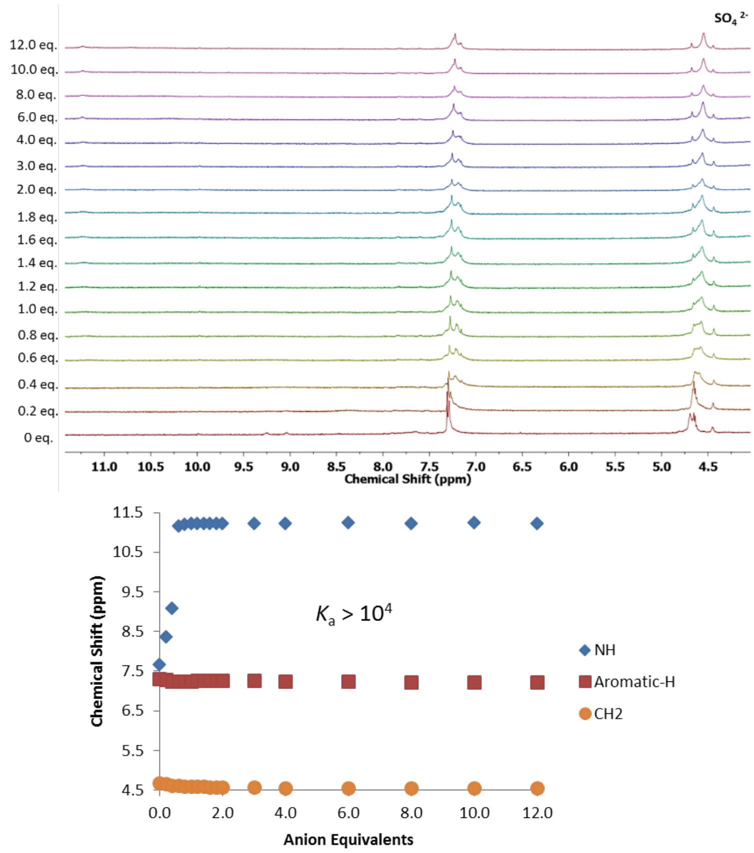

NMR stackplot (left) and changes observed in squaramide NH, aromatic CH and methylene proton (right).

Figure S26: ^{1}H NMR titration of **5** in 1:2 (v/v) H_2O:DMSO-d_6 mixture at 300 K. (a): with $(TBA)_2SO_4$ (0 -12 eq.). (b): with $TBAH_2PO_4$ (0 -12 eq.). (c): with $(TBA)_2SeO_4$ (0 -12 eq.). (d): with $(TBA)_2CrO_4$ (0 -12 eq.). (e): with $(TBA)_2CrO_4$ (0 -12 eq.). (f): with $TBAHCO_3$ (0 -12 eq.). (g): with $TBANO_3$ (0 -12 eq.). (h): with TBAAcO (0 -12 eq.). (i): with $TBABF_4$ (0 -12 eq.). (j): with $TBAClO_4$ (0 -12 eq.). (k): with TBA(*p*-toluenesulfonate) (0 -12 eq.). (l): with $TBAReO_4$ (0 -12 eq.).

(a): ^{1}H NMR titration of **5** with $(TBA)_2SO_4$ (0 -12 eq.) in DMSO-d_6 at 300 K.

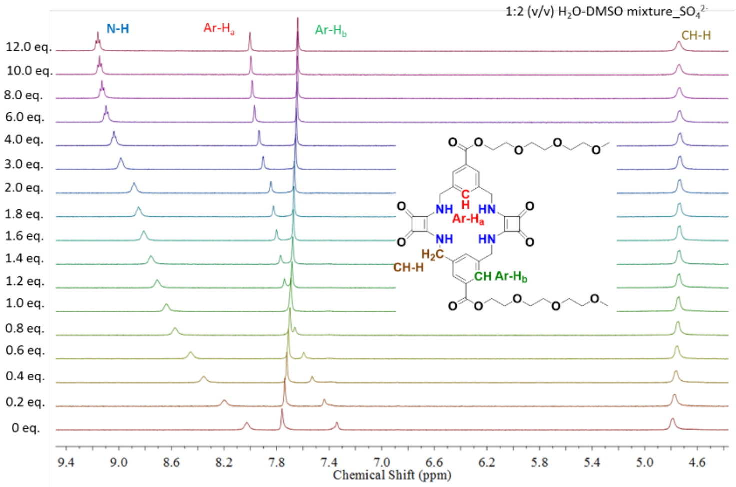

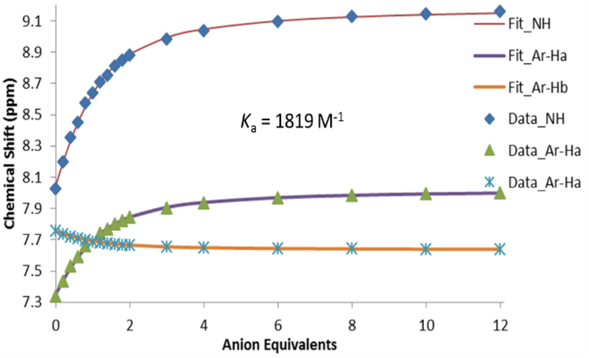

(b): ^{1}H NMR titration of **5** with $TBAH_2PO_4$ (0 -12 eq.) in DMSO-d_6 at 300 K.

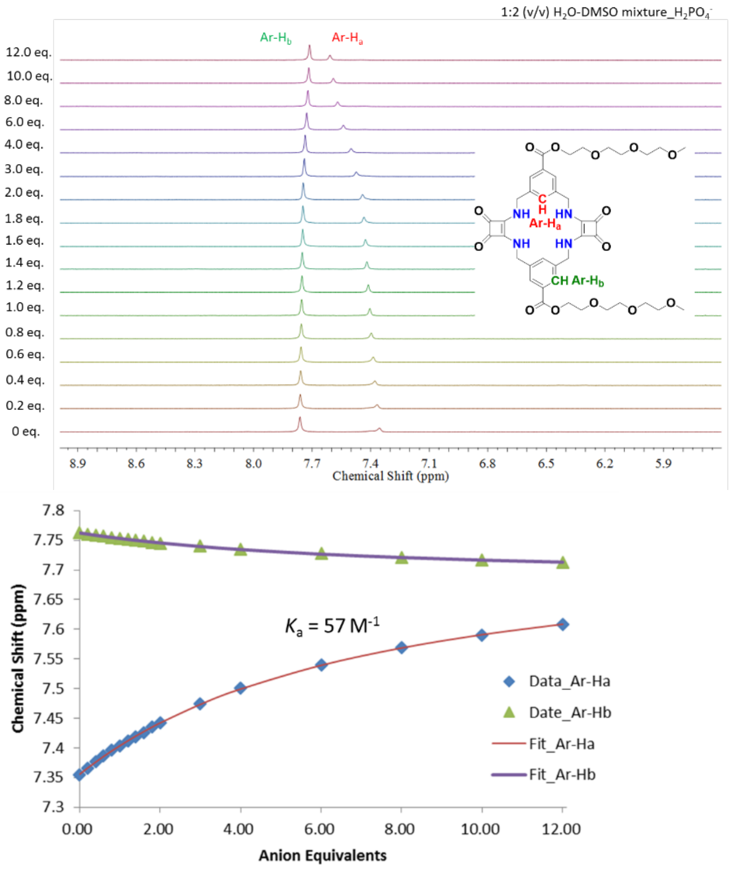

(c): ^{1}H NMR titration of **5** with $(TBA)_2SeO_4$ (0 -12 eq.) in DMSO-d_6 at 300 K.

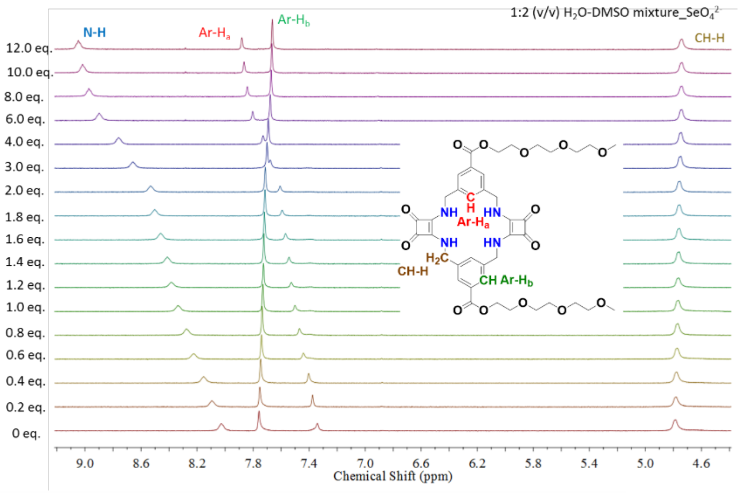

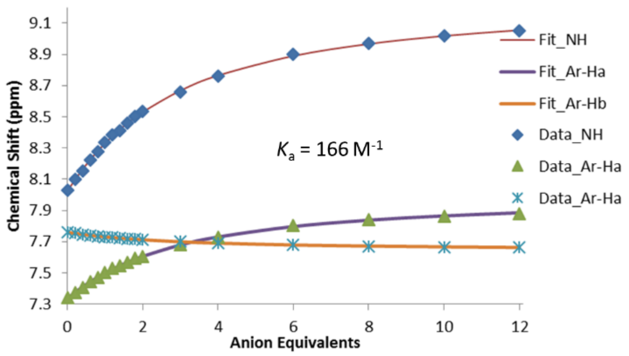

(d): ^{1}H NMR titration of **5** with $(TBA)_2Cr_2O_7$ (0 -12 eq.) in DMSO-d_6 at 300 K.

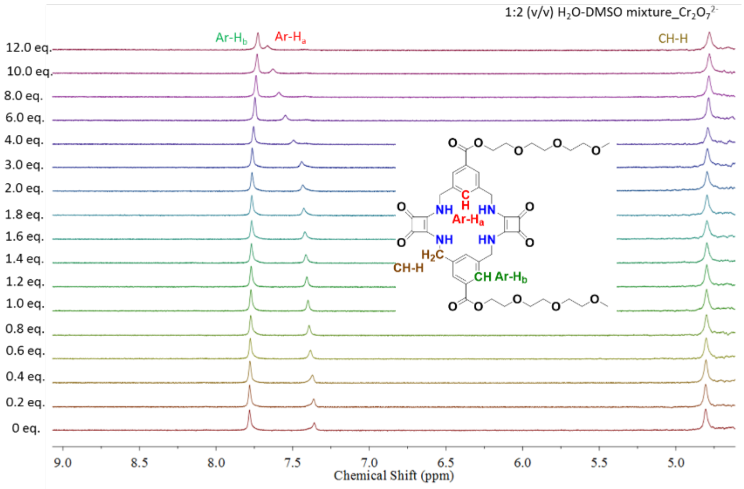

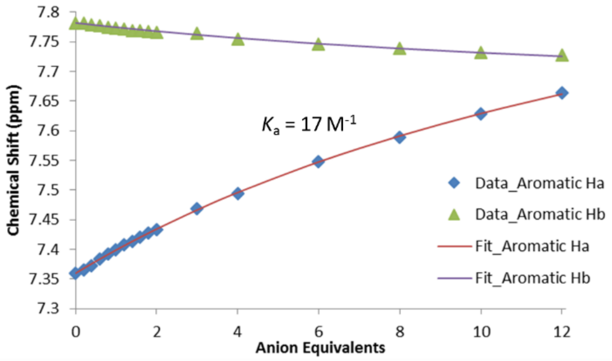

(e): ^{1}H NMR titration of **5** with $(TBA)_2CrO_4$ (0 -12 eq.) in DMSO-d_6 at 300 K.

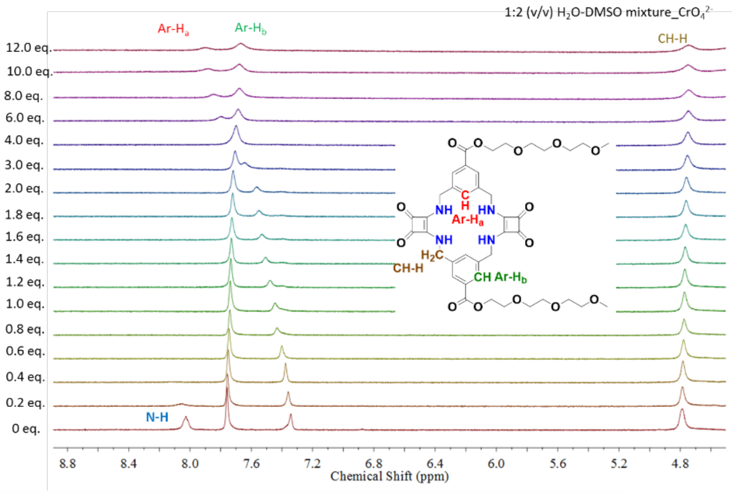

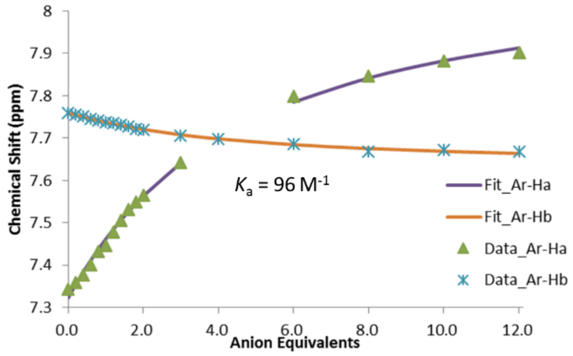

(f): ^{1}H NMR titration of **5** with $TBAHCO_3$ (0 -12 eq.) in DMSO-d_6 at 300 K.

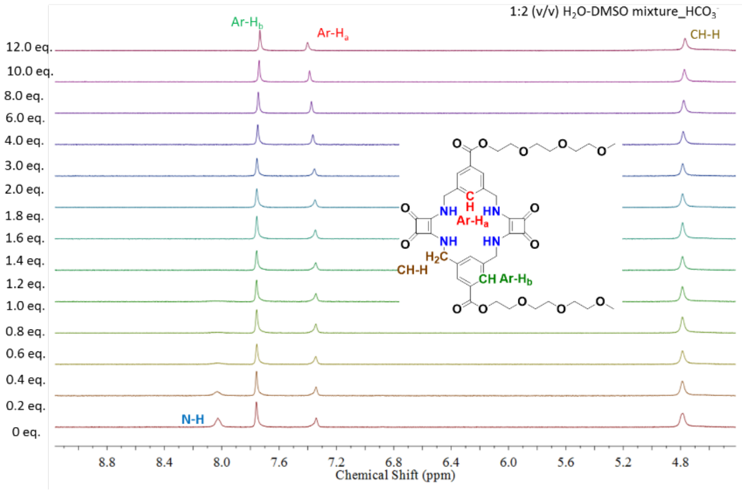

(g): ^{1}H NMR titration of **5** with $TBANO_3$ (0 -12 eq.) in DMSO-d_6 at 300 K.

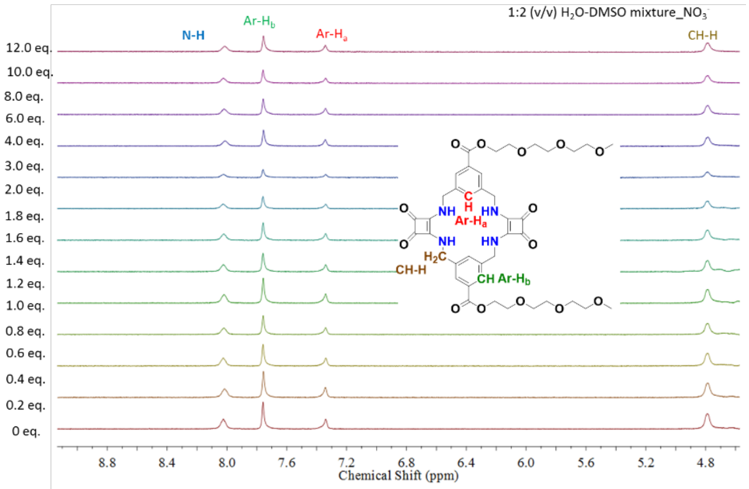

(h): ^{1}H NMR titration of **5** with TBAAcO (0 -12 eq.) in DMSO-d_6 at 300 K.

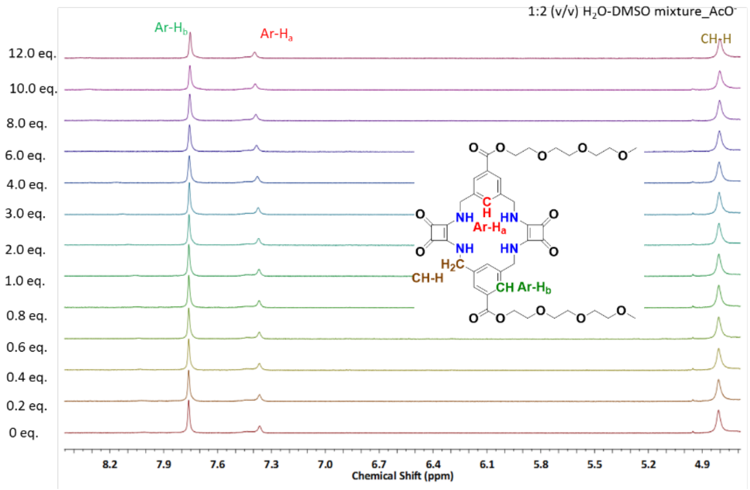

(i): ^{1}H NMR titration of **5** with $TBABF_4$ (0 -12 eq.) in DMSO-d_6 at 300 K.

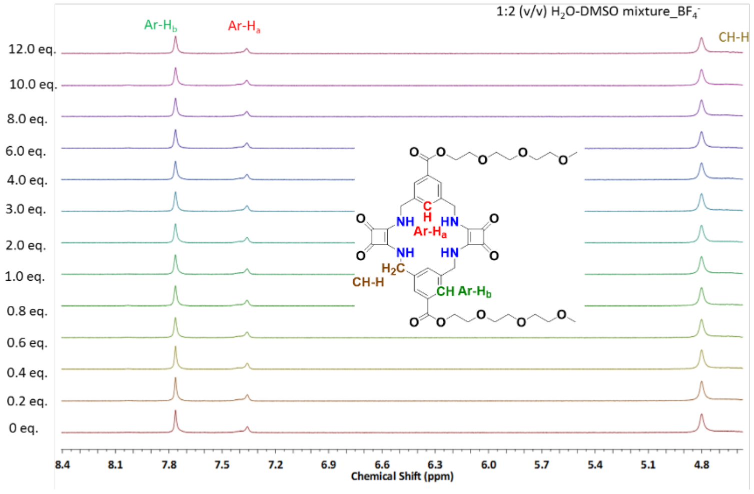

(j): ^{1}H NMR titration of **5** with $TBAClO_4$ (0 -12 eq.) in DMSO-d_6 at 300 K.

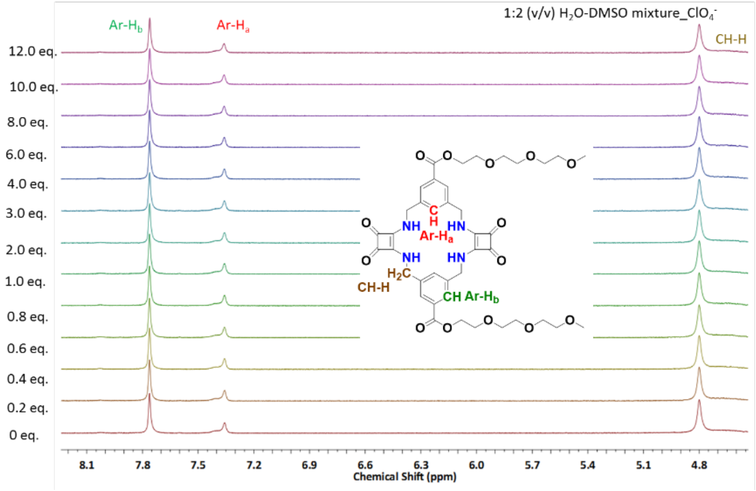

(k): ^{1}H NMR titration of **5** with TBA(*p*-toluenesulfonate) (0 -12 eq.) in DMSO-d_6 at 300 K.

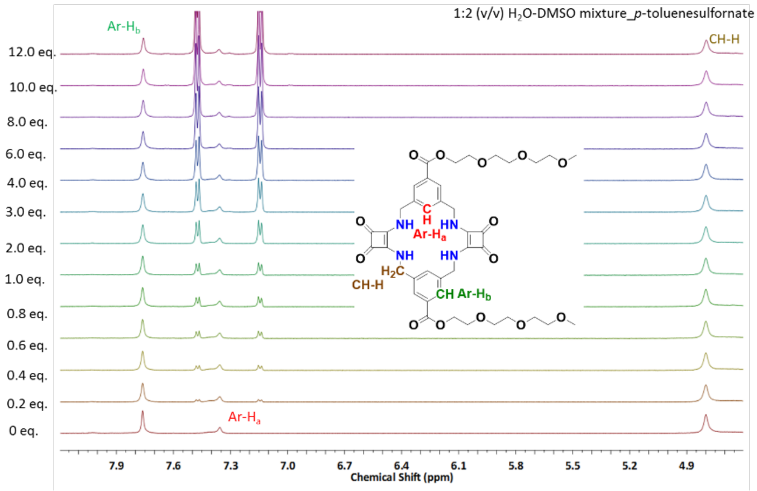

(l): ^{1}H NMR titration of **5** with $TBAReO_4$ (0 -12 eq.) in DMSO-d_6 at 300 K.

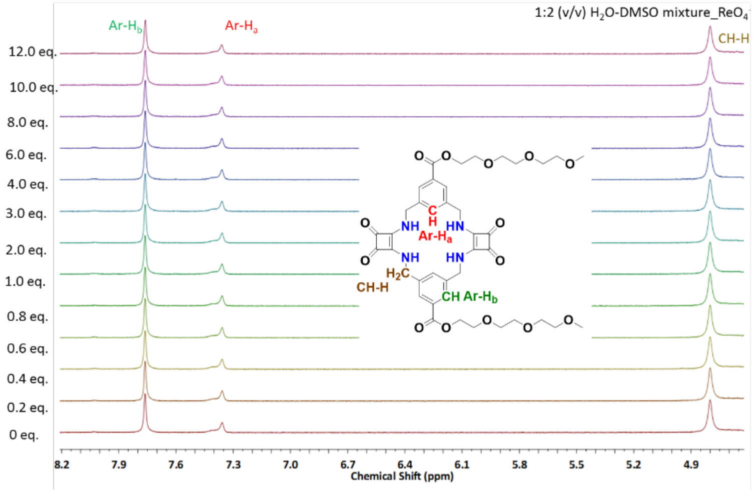

Figure S27: ^{1}H NMR titration of **5** in 1:2 (v/v) H_2O:DMSO-*d₆* Tris buffer (15mM, pH 9.1) mixture at 300 K. (a): with $(TBA)_2SO_4$ (0 -12 eq.). (b): with $(TBA)_2HPO_4$ (0 -12 eq.).

(a): ^{1}H NMR titration of **5** in 1:2 (v/v) H_2O:DMSO-d_6 Tris buffer (15mM, pH 9.1) mixture at 300 K $(TBA)_2SO_4$ (0 -12 eq.).

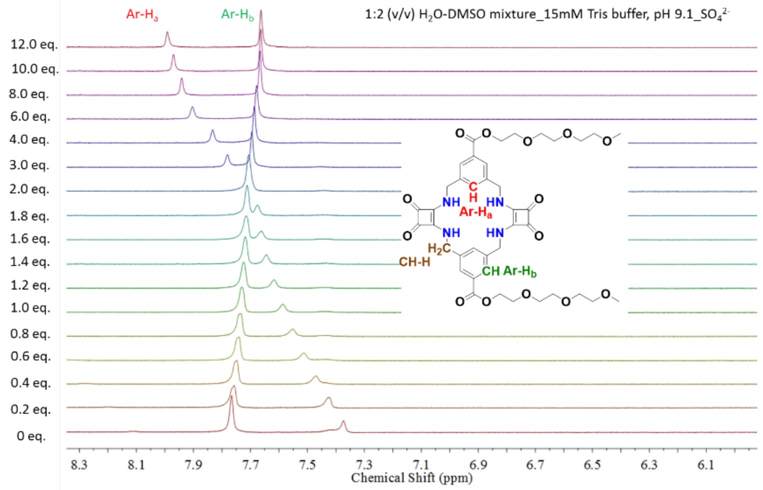

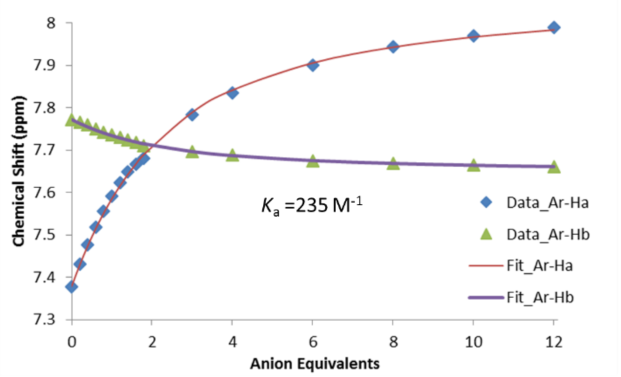

(b): ^{1}H NMR titration of **5** in 1:2 (v/v) H_2O:DMSO-d_6 Tris buffer (15mM, pH 9.1) mixture at 300 K with $(TBA)_2HPO_4$ (0 -12 eq.).

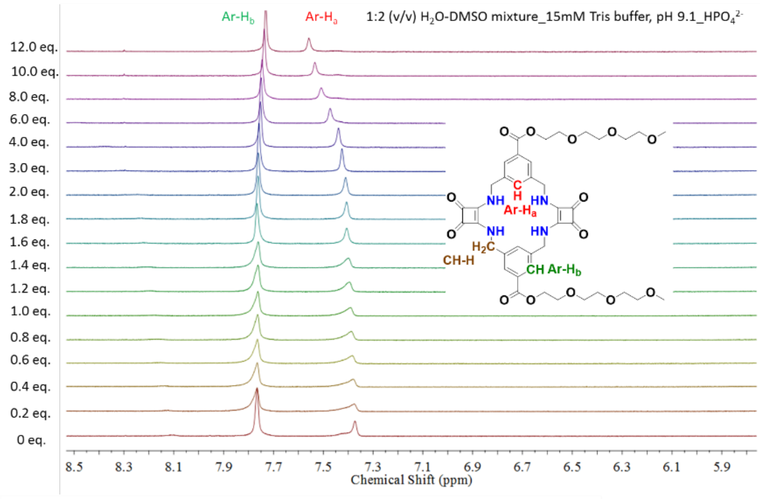

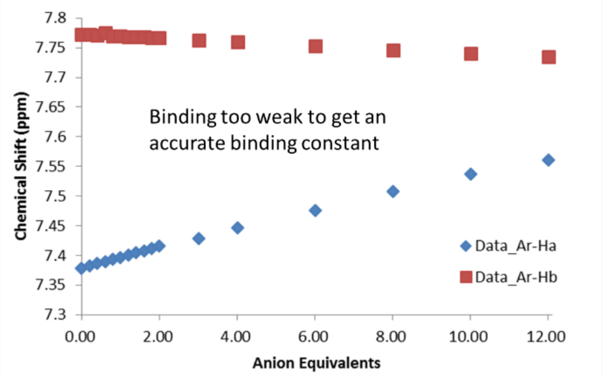

Figure S28: ^{1}H NMR titration of **6** in 1:2 (v/v) H_2O:DMSO-d_6 mixture at 300 K. (a): with $(TBA)_2SO_4$ (0 -12 eq.). (b): with $TBAH_2PO_4$ (0 -12 eq.). (c): with $(TBA)_2SeO_4$ (0 -12 eq.). (d): with $(TBA)_2CrO_4$ (0 -12 eq.). (e): with $(TBA)_2CrO_4$ (0 -12 eq.). (f): with $TBAHCO_3$ (0 -12 eq.). (g): with $TBANO_3$ (0 -12 eq.). (h): with TBAAcO (0 -12 eq.). (i): with $TBABF_4$ (0 -12 eq.). (j): with $TBAClO_4$ (0 -12 eq.). (k): with TBA *p*-toluenesulfornate (0 -12 eq.). (l): with $TBAReO_4$ (0 -12 eq.).

(a): ^{1}H NMR titration of **6** with $(TBA)_2SO_4$ (0 -12 eq.) in DMSO-d_6 at 300 K.

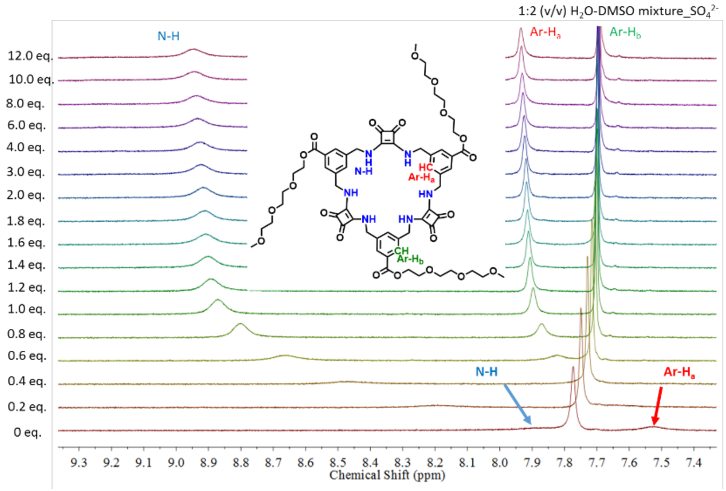

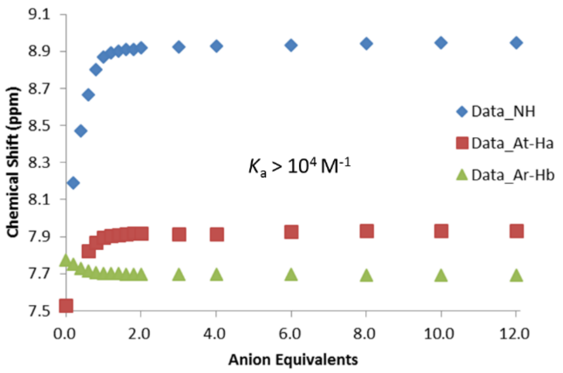

NMR stackplot (left) and changes observed in squaramide NH, aromatic CH and methylene proton (right).

(b): ^{1}H NMR titration of **6** with $TBAH_2PO_4$ (0 -12 eq.) in DMSO-d_6 at 300 K.

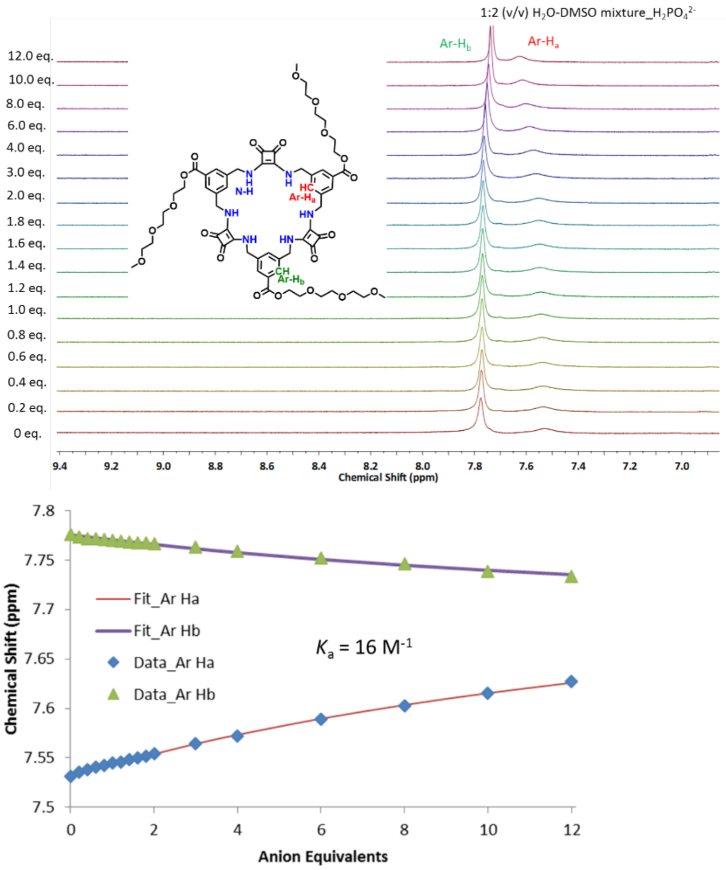

(c): ^{1}H NMR titration of **6** with $(TBA)_2SeO_4$ (0 -12 eq.) in DMSO-d_6 at 300 K.

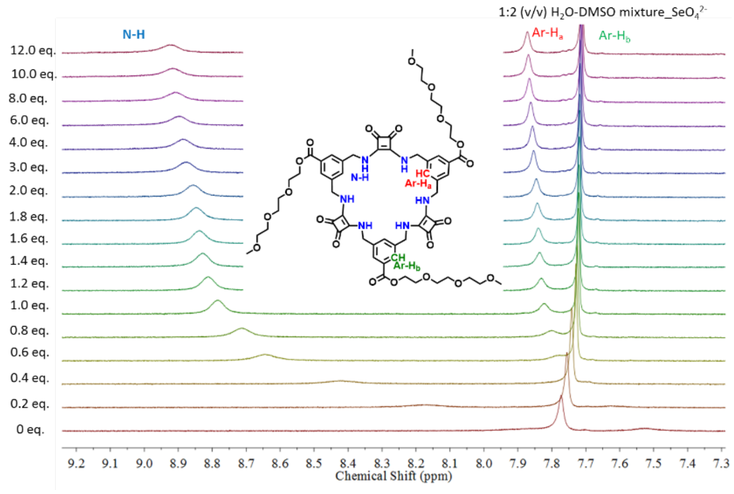

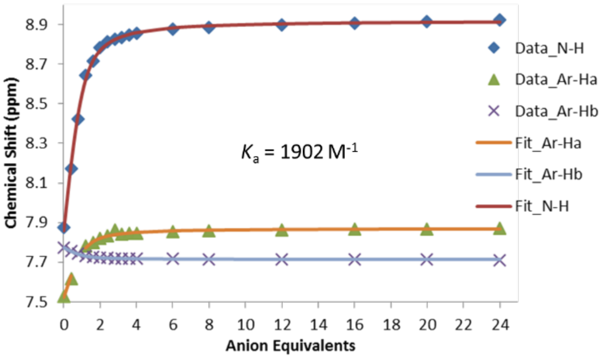

(d): ^{1}H NMR titration of **6** with $(TBA)_2Cr_2O_7$ (0 -12 eq.) in DMSO-d_6 at 300 K.

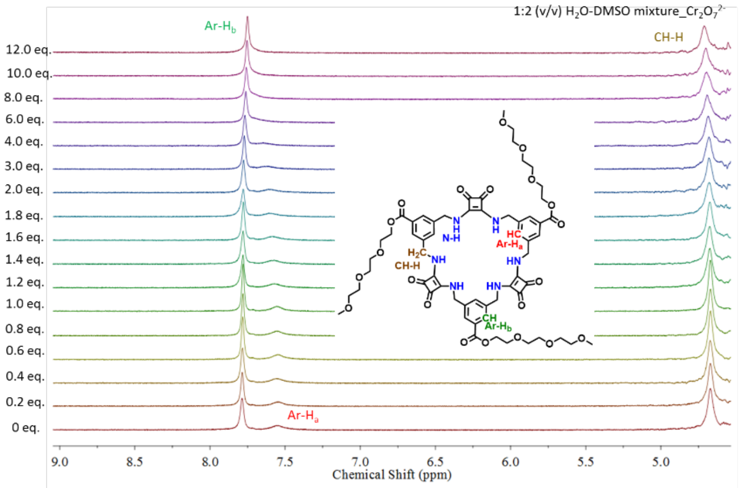

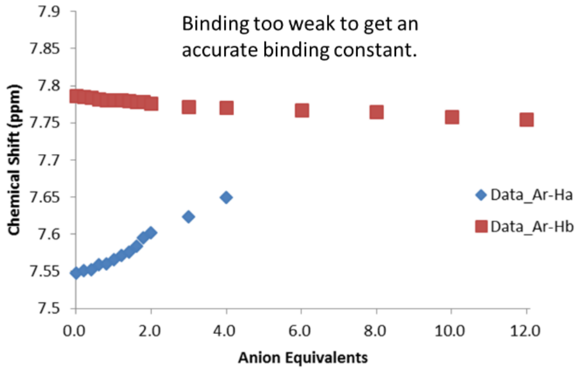

(e): ^{1}H NMR titration of **6** with $(TBA)_2CrO_4$ (0 -12 eq.) in DMSO-d_6 at 300 K.

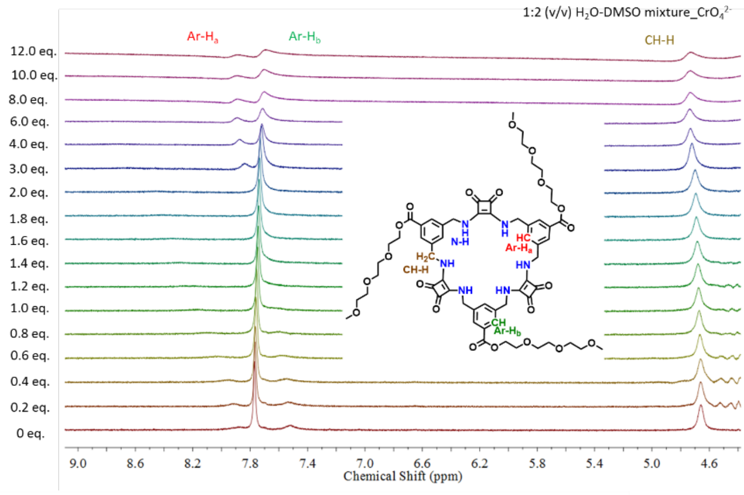

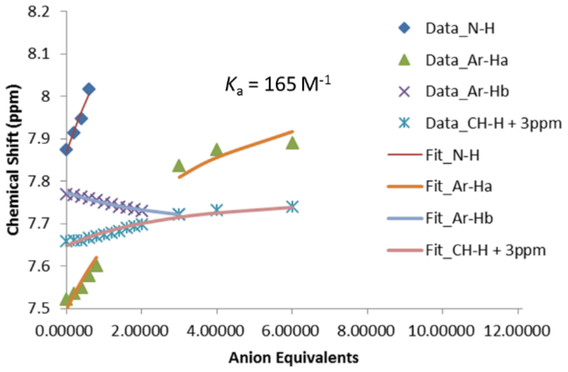

(f): ^{1}H NMR titration of **6** with $TBAHCO_3$ (0 -12 eq.) in DMSO-d_6 at 300 K.

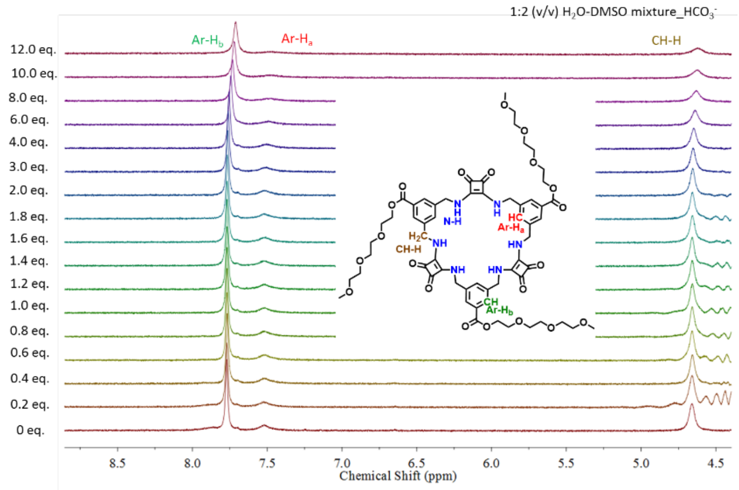

(g): ^{1}H NMR titration of **6** with $TBANO_3$ (0 -12 eq.) in DMSO-d_6 at 300 K.

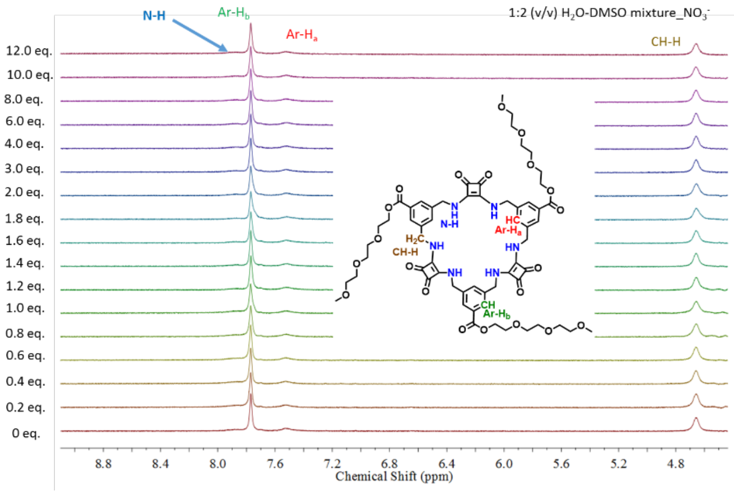

(h): ^{1}H NMR titration of **6** with TBAAcO (0 -12 eq.) in DMSO-d_6 at 300 K.

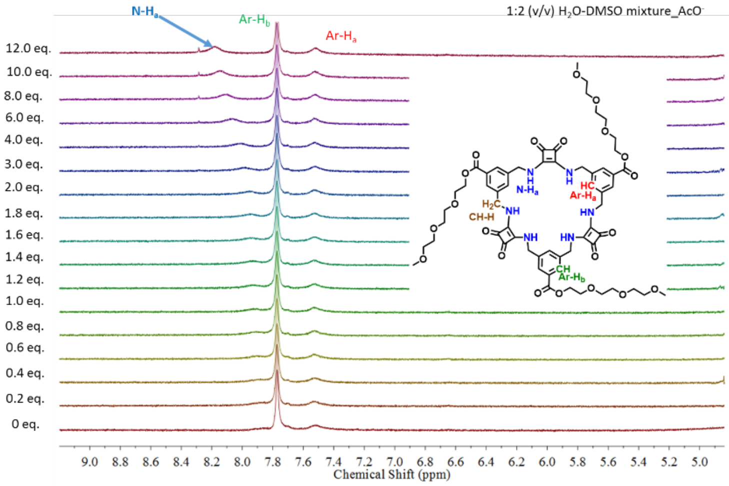

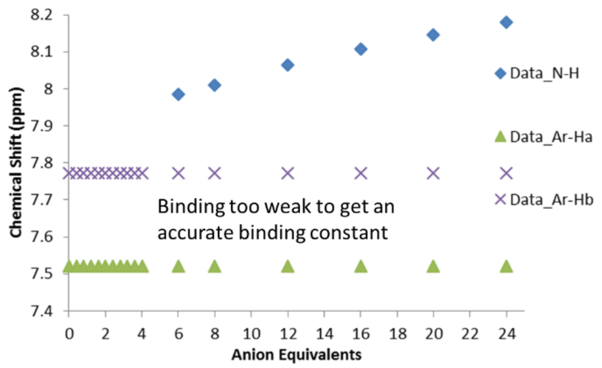

(i): ^{1}H NMR titration of **6** with $TBABF_4$ (0 -12 eq.) in DMSO-d_6 at 300 K.

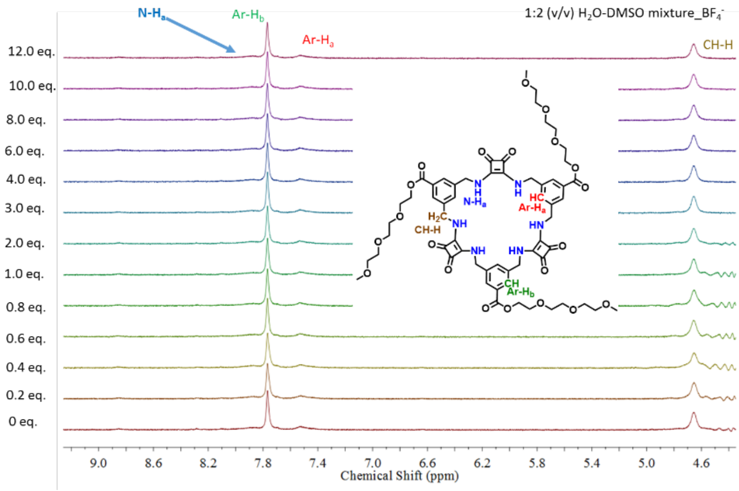

(j): ^{1}H NMR titration of **6** with $TBAClO_4$ (0 -12 eq.) in DMSO-d_6 at 300 K.

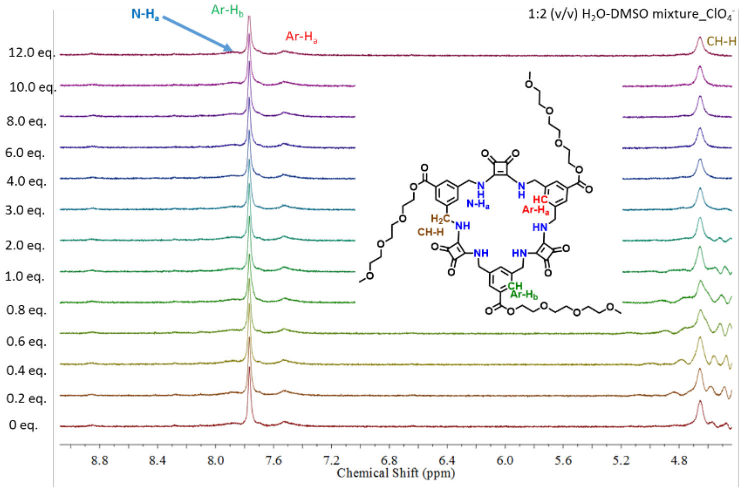

(k): ^{1}H NMR titration of **6** with TBA(*p*-toluenesulfonate) (0 -12 eq.) in DMSO-d_6 at 300 K.

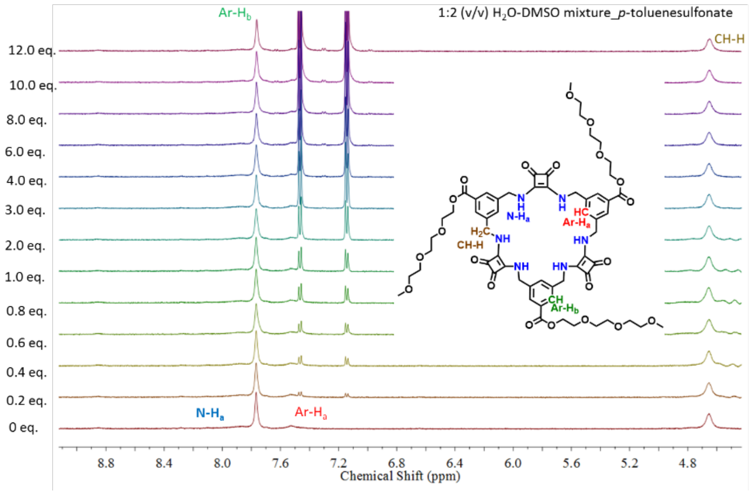

(l): ^{1}H NMR titration of **6** with TBAReO$_4$ (0 -12 eq.) in DMSO-d_6 at 300 K.

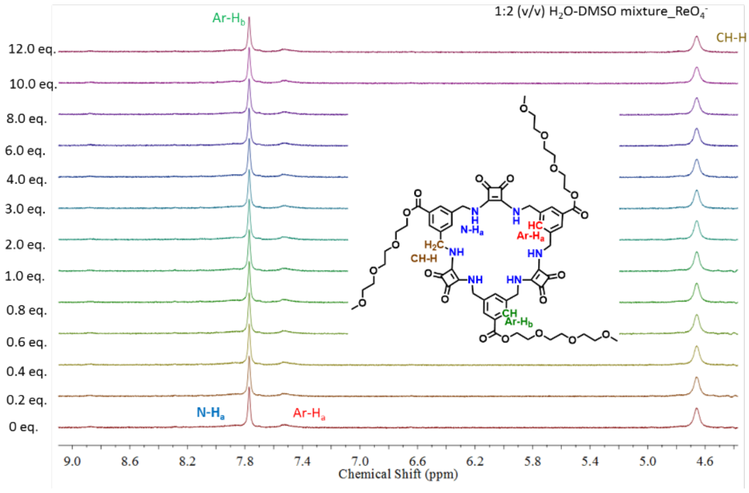

Figure S29: ^{1}H NMR titration of **6** in 1:2 (v/v) H_2O:DMSO-d_6 Tris buffer (15mM, pH 9.1) mixture at 300 K. (a): with $(TBA)_2SO_4$ (0 -12 eq.). (b): with $(TBA)_2HPO_4$ (0 -12 eq.).

(a): ^{1}H NMR titration of **6** in 1:2 (v/v) H_2O:DMSO-d_6 Tris buffer (15mM, pH 9.1) mixture at 300 K with $(TBA)_2SO_4$ (0 -12 eq.)

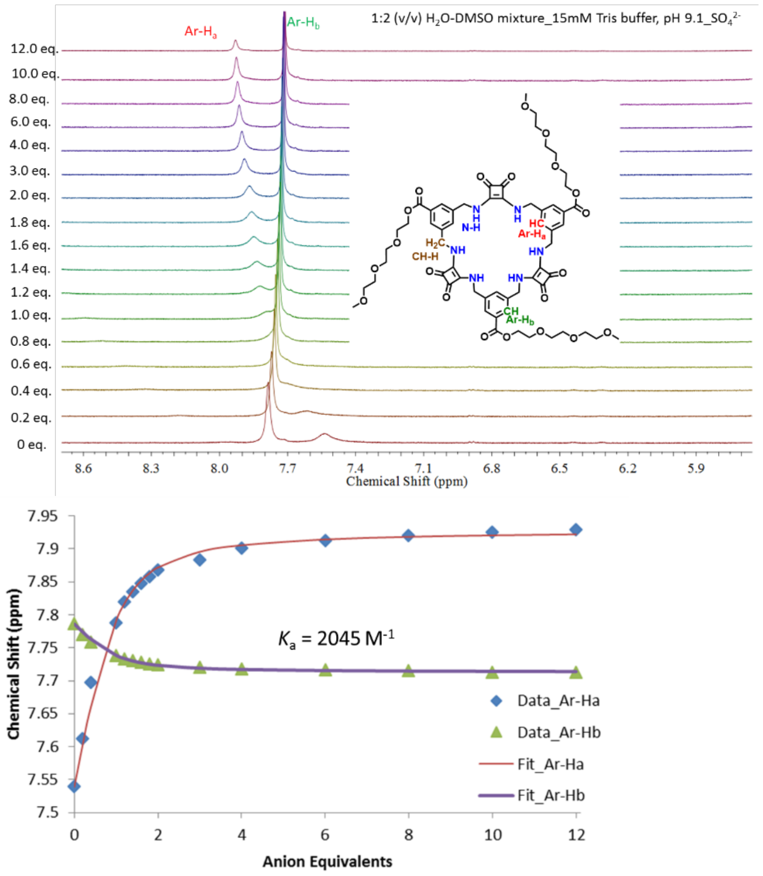

(b): ^{1}H NMR titration of **6** in 1:2 (v/v) H_2O:DMSO-d_6 Tris buffer (15mM, pH 9.1) mixture at 300 K with $(TBA)_2HPO_4$ (0 -12 eq.).

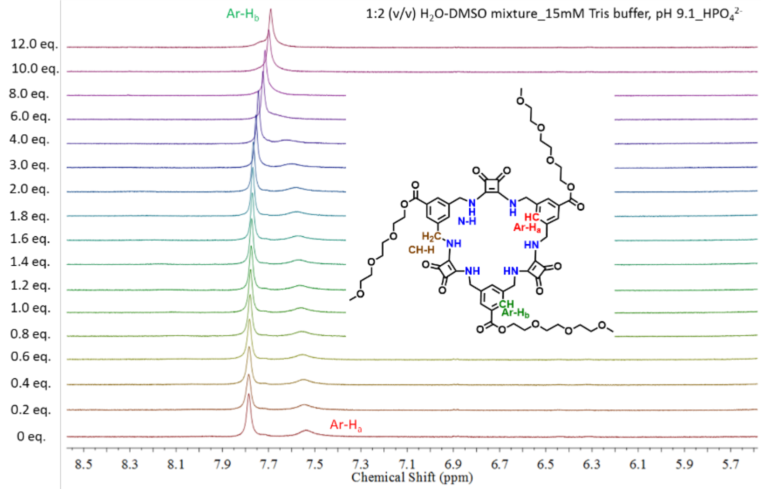

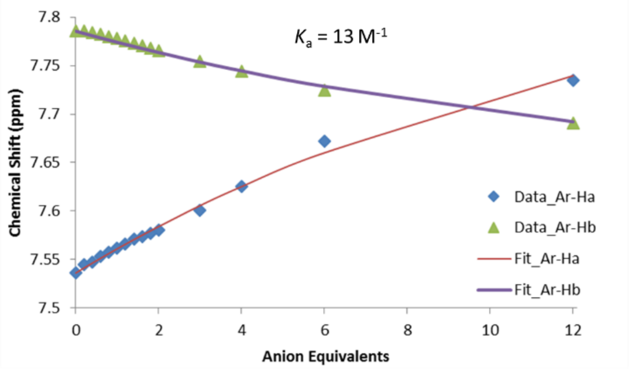

Figure S30: ^{1}H NMR SO_4^{2-} titration of **6** in 1:1 (v/v) H_2O-DMSO mixture (20mM Tris buffer, pH 7.4) with 1.5mM phosphates, 106mM Cl^-, 28 mM H_2CO_3/HCO_3^-.

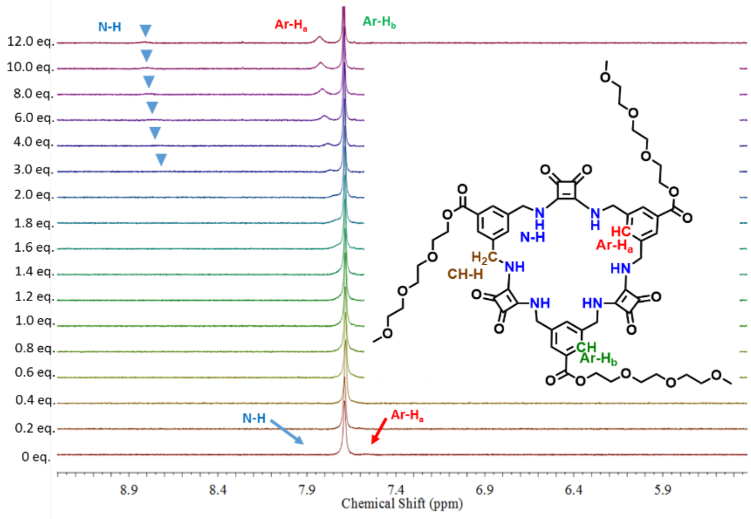

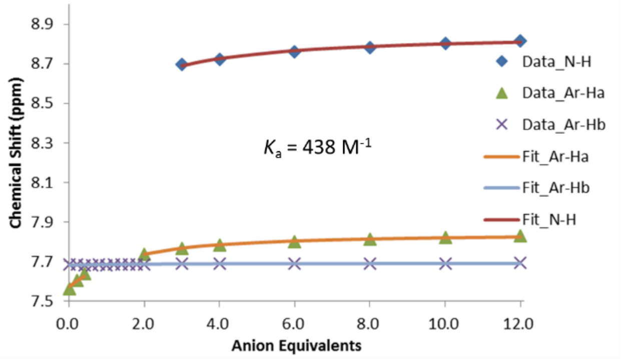

Figure S31. Modelled structure of the 1•SO_4^{2-} complex

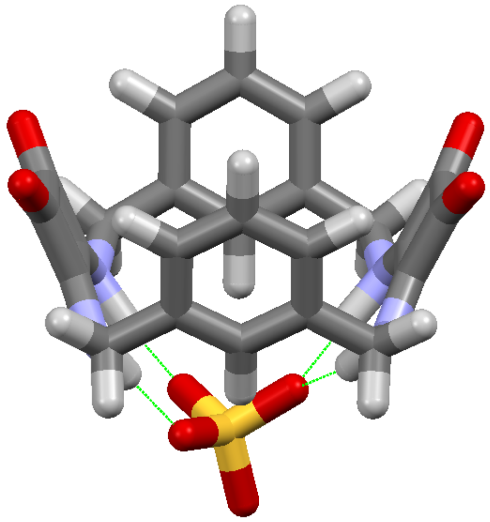

Side view

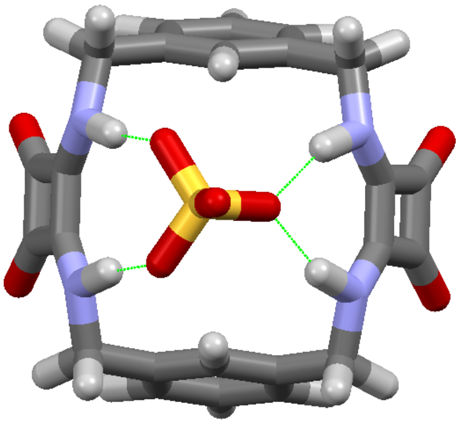

Top view

Single Crystal X-ray Diffraction Analysis Summary

Single crystals were attached with Exxon Paratone N to a nylon loop and quenched in a cold nitrogen gas stream from an Oxford Cryosystems Cryostream. The structures were solved with direct methods and refined with SHELXL-2014/7.[1] Solution and refinement were facilitated with WinGX[2] and ShelXle[3], and ORTEP3[4] for Windows was used to generate ORTEP[4] depictions of the molecules with 50% displacement ellipsoids (Figs. S1 to S3 and Fig. S5). Additional images were generated with Mercury[5] and least squares planes and additional geometry were calculated using XTAL.[6] A summary of crystallographic parameters is provided in Table S1 and a summary of squaramide macrocycle cavity geometry is given in Table S2.

Compound 1

Data were collected from a pale yellow prismatic crystal using a SuperNova Dual diffractometer equipped with an Atlas detector and employing mirror monochromated Cu (Kα) radiation from a micro-source. Cell constants were obtained from a least squares refinement against 4,050 reflections located between 9 and 152° 2θ. Data were collected at 150(1) Kelvin with ω scans to 152° 2θ. The data processing was undertaken with CrysAlis Pro[7] and a multi-scan absorption correction was applied[7] to the data.

The structure was solved in the space group $P\ \bar{1}$(#2) by direct methods with SIR97.[8] The asymmetric unit contains half of the squaramide macrocycle centred about an inversions site, together with a dimethyl sulfoxide solvate molecule. The non-hydrogen atoms in the asymmetric unit were modelled with anisotropic displacement parameters and in general a riding atom model was used for the hydrogen atoms. The amine hydrogen atoms were located and modelled with isotropic displacement parameters; the solvate and squaramide macrocycle are linked by hydrogen bond interactions (see Fig. S32). An ORTEP depiction of the molecule with 50% displacement ellipsoids is provided in Figure S32, where the superscript on the atom numbers refers to the symmetry operation -x, -y, 1-z.

As Figure S31 indicates, both squaramide moieties have hydrogen bond interactions with a DMSO solvate molecule:

Donor	Hydrogen	Acceptor	D-H(Å)	H-A(Å)	D-A(Å)	DHA Angle(°)
N(1)	H(1N)	O(3)	0.85(2)	2.02(2)	2.8332(15)	158.9(17)
N(2)	H(2N)	O(3)	0.872(19)	1.962(19)	2.7975(16)	160.0(17)

The length and width of the cavity is effectively determined by the separation between the four ‘corner’ carbon atoms that link the phenyl groups and squaramide groups and in **1** these distances are 6.062(2) Å across the squaramide group (distance between C5 and C12^{i}) and 5.075(2) Å across the phenyl group (distance between C5 and C12). The cavity shape is essentially rectangular, with unique internal corner angles of approximately 87.1° and 92.9 °.

Related by inversion, the least squares planes defined by the carbon atoms of the two squaramide groups are parallel and separated by 4.832(6)Å. The centroid to centroid distance is 7.233(2)Å and the relative lateral displacement or offset of the squaramide groups is approximately 5.38Å.

The least squares planes defined by the carbon atoms of the two phenyl groups are separated by a perpendicular distance of 3.672(5)Å and the centroid to centroid distance is 8.463(2)Å. The relative lateral displacement of the squaramide groups is 7.62Å.

The cavity plane is effectively defined by the four 'corner' carbon atoms that link the phenyl groups to the squaramide moieties (C5, C12, $C5^{i}$, $C12^{i}$) and the phenyl carbon located on the cavity edge (C11and $C11^{i}$). The largest deviation of a defining atom from the least squares plane defined with respect to these atom sites is 0.034(2)Å (C11).

The least squares planes of the squaramide and phenyl groups are respectively inclined at an angle of 60.65(6)° and 37.72(4)° with respect to the least squares plane of the cavity. The amide torsion angles (C7-C6-C5-N2 and C9-C10-C12-$N1^{i}$) are 125.3(1) and -152.5 (1) °. The nitrogen atoms are displaced 0.340(1)Å and 0.286(1)Å from the cavity plane.

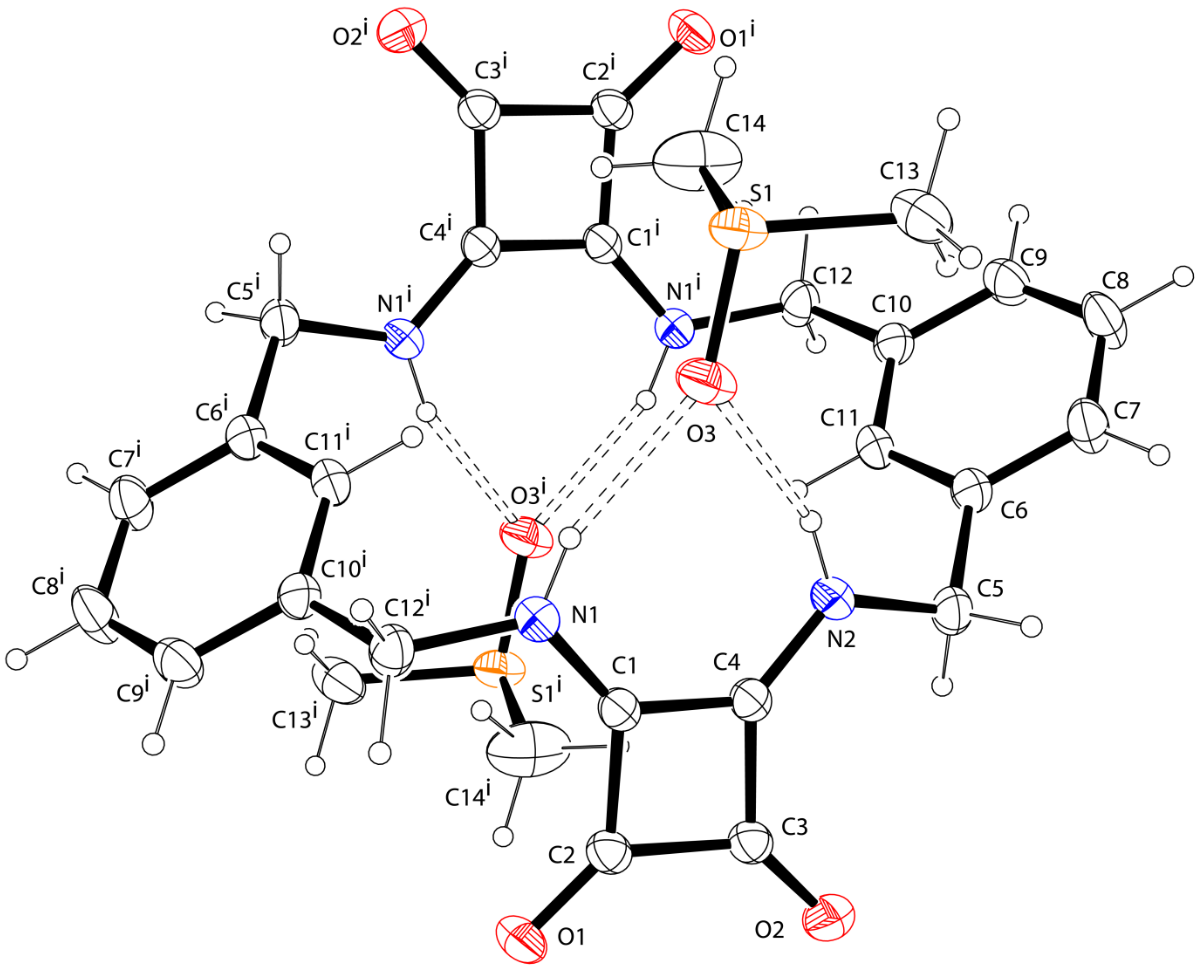

Figure S32. ORTEP depiction for **1** with 50% ellipsoids; the superscript on the atom numbers refers to the symmetry operation -x, -y, 1-z.

Compound 3

Data were collected from a colourless prismatic crystal mounted on a SuperNova Dual diffractometer equipped with an Atlas detector, with mirror monochromated Cu(Kα) radiation from a micro-source. Cell constants were obtained from a least squares refinement against 26,314 reflections located between 5 and 153° 2θ. Data were collected at 150(1) Kelvin with ω scans to 153° 2θ. The data processing was undertaken with CrysAlis Pro.[7]

The structure was solved in the space group $P\ \bar{1}$(#2) by direct methods with SHELXS-97.[1] The non-hydrogen atoms in the asymmetric unit were modelled with anisotropic displacement parameters and in general the hydrogen sits were modelled with isotropic displacement parameters. The amine nitrogen sites were located and modelled with a distance restraint and isotropic displacement parameter. The macrocycle is centred about an inversion site. An ORTEP depiction of the molecule with 50% displacement ellipsoids is provided in Figure S33, where the superscript on the atom numbers refers to the symmetry operation 1-x, -y, -z.

As Figure S33 depicts, the structure has complementary hydrogen bond interactions between the squaramide groups of adjacent molecules that link them in a chain like manner, with the chain running approximately across the (011) plane in the [01-1] direction.

The squaramide hydrogen bond geometry is summarised as follows:

Donor	Hydrogen	Acceptor		D-H(Å)	H-A(Å)	D-A(Å)	DHA Angle(°)
N(1)	H(1N)	O(3)		0.883(15)	2.018(15)	2.8594(17)	159.0(18)
N(2)	H(2N)	O(2)	2_646	0.899(14)	2.008(16)	2.8523(16)	155.8(17)

Where 2_646 corresponds to the symmetry operation 1-x, -y-1,1-z.

The length and width of the cavity is effectively determined by the separation between the four ‘corner’ carbon atoms that link the phenyl groups and squaramide groups and in **3** these distances are 4.732(3)Å across the squaramide group (C5 to $C12^{i}$) and 5.837(2)Å across the phenyl group (distance between C5 and C12). The cavity is shaped like a parallelogram with internal corner angles of 82.6° and 97.4°.

Related by inversion, the least squares planes defined by the carbon atoms of the two squaramide groups are parallel and separated by 6.288(6)Å. The centroid to centroid distance is 8.590(2)Å and the relative lateral displacement or offset of the squaramide groups is approximately 5.85Å.

The least squares planes defined by the carbon atoms of the two phenyl groups are separated by 4.635(2)Å and the centroid to centroid distance is 4.667(2)Å. The relative lateral displacement of the phenyl groups is approximately 0.55Å.

The cavity plane is effectively defined by the four carbon atoms that link the phenyl groups to the squaramide moieties (C5, C12, $C5^{i}$, $C12^{i}$) and the phenyl carbon atoms to which they are bonded (C6, C9, $C6^{i}$, $C9^{i}$). The largest deviation of a defining atom from the least square plane defined with respect to these atom sites is 0.021(2) Å (C9).

The least squares planes of the squaramide and phenyl groups are respectively inclined at an angle of 79.66(6)° and 88.30(4)° with respect to the least squares plane of the cavity. The amide torsion

angles defined by the edge of the phenyl group, the bond to the linking carbon and from that carbon to the amide nitrogen (C7-C6-C5,-N2 and C9-C10-C12-N1^{i}) are -81.8(2) and 164.9(2)º. As a consequence of this marked difference, one nitrogen (N1) is displaced 1.266(2)Å from the least squares plane of the cavity, while the second (N2) is 0.1434(2)Å from that plane.

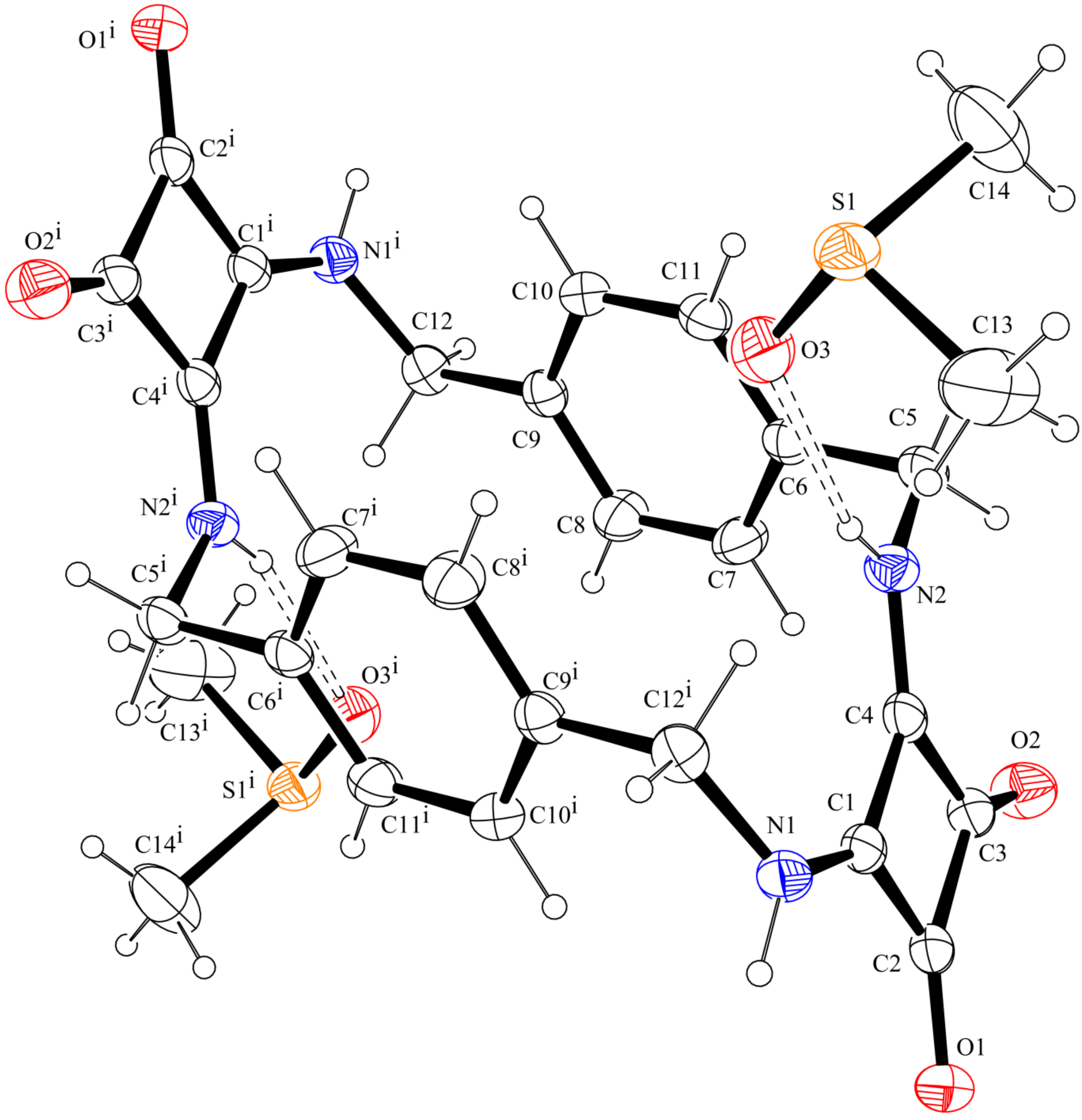

Figure S33. ORTEP depiction for **3** with 50% ellipsoids; the superscript on the atom numbers refers to the symmetry operation 1-x, -y, -z.

Complex 1-SO_4^{2-}

Data were collected from a colourless prismatic crystal mounted on an APEXII-FR591 diffractometer employing mirror monochromated MoKα radiation generated from a rotating anode. Cell constants were obtained from a least squares refinement against 18,518 reflections located between 5 and 59° 2θ. Data were collected at 100(1) Kelvin with ω+φ scans to 51° 2θ. The data processing was undertaken with APEX, SAINT and XPREP[8] and an empirical absorption correction determined with SADABS[9] was applied to the data

The structure was solved in the space group $P2_1/c$(#14) by direct methods with SHELXS-97.[1] The asymmetric unit contains the **1-SO_4^{2-}** complex anion, together with two tetrabutylammonium cations and two water molecules. The water hydrogens were located in final difference maps and their positions were refined and modelled with isotropic displacement parameters. In general the non-hydrogen atom sites were modelled with anisotropic displacement parameters and a riding atom model with isotropic displacement parameters was used for the hydrogen sites. The sulfate and part of a butyl residue on one of the cations are disordered over two orientations. Occupancies for the disordered butyl residue were refined and then fixed at 0.65 and 0.35.

Occupancies for the disordered sulfate sites were refined and then fixed at 0.75 and 0.25. The sulfate is bound to the macrocycle by hydrogen bonds and the disorder reflects two binding modes, with the dominant mode involving all four amide nitrogen protons and three sulfate oxygen atoms and the minor mode just two oxygen atoms (see Fig. S34). Isotropic displacement parameters were used for the minor occupancy sites. An ORTEP depiction of the molecule with 50% displacement ellipsoids is provided in Figure S34.

The length and width of the cavity is effectively determined by the separation between the four ‘corner’ carbon atoms that link the phenyl groups and squaramide groups and in **1** these distances are 6.070 (3) and 6.041(2) Å across the two squaramide groups (C5 to C24 and C12 to C17 distance respectively), and 5.050(4) and 4.998(3)Å across the phenyl groups (C5 to C12 and C17 to C24 respectively). The cavity shape is essentially rectangular, with internal corner angles of approximately 89.1° (at C5), 90.6° (at C12), 89.9° (at C17) and 90.6° (at C24).

The two least squares planes defined by the squaramide carbon atoms are inclined with respect to each other by 73.5(1)° and the two least squares planes of the phenyl groups form an angle of 50.78(8)° with respect to each other. The centroid to centroid distances between the squaramide groups is 7.152(3) Å and that for the phenyl groups is 7.323(2) Å.

The cavity plane is effectively defined by the four ‘corner’ carbon atoms that link the phenyl groups to the squaramide moieties (C5, C12, C17, C24) and the phenyl carbon located on the cavity edge (C11and C23). The largest deviation of a defining atom from the least squares plane defined with respect to these atoms is 0.060(2)Å (C23). The amide torsion angles (C7-C6-C5-N2, C9-C10-C12-N3, C19-C18-C17-N4 and C21-C22-C24-N1) are 136.5(3), -136.0(2), 112,4(2) and -111.5 (2)°. The nitrogen deviations from the plane are 0.393(2) (N1), 0.379(2) (N2),0.334(2) (N3) and 0.459(2) Å (N4).

The least squares planes of the squaramide are inclined at 57.39(9)° (C1-C2-C3-C4) and 49.12(8)° (C13-C14-C15-C16) with respect to the least squares plane of the cavity. The phenyl least squares planes are inclined at 52.68(6) ° (C6-C7-C8-C9-C10-C11) and 76.55(6)° (C18-C19-C20-C21) with respect to the cavity least squares plane.

The water molecules link adjacent macrocycle-sulfate complexes, with hydrogen bonds to sulfate oxygens and to a carbonyl oxygen of an adjacent macrocycle. The hydrogen bond linkage forms a chain extending in the direction of the *a* axis of the unit cell. In the asymmetric unit the anionic complex is 'nestled' between the pendant residues of a pair of tetrabutylammonium cations (see Fig. S35).

Key hydrogen bond geometry:

Donor	Hydrogen	Acceptor		D-H(Å)	H-A(Å)	D-A(Å)	DHA Angle(º)
N(1)	H(1N)	O(5)		0.88	2.09	2.847(2)	143.4
N(2)	H(2N)	O(5)		0.88	1.95	2.745(2)	148.7
N(3)	H(3N)	O(6A)		0.88	1.94	2.801(2)	165.4
N(3)	H(3N)	O(8B)		0.88	2.04	2.846(6)	151.2
N(4)	H(4N)	O(8A)		0.88	2.08	2.881(2)	151.5
N(4)	H(4N)	O(8B)		0.88	1.85	2.633(6)	146.8
O(1W)	H(1WA)	O(3)	2_545	0.91(4)	2.10(4)	2.951(2)	155(3)
O(1W)	H(1WB)	O(6A)		0.91(3)	2.04(3)	2.866(3)	151(3)
O(1W)	H(1WB)	O(6B)		0.91(3)	2.15(3)	3.033(6)	165(3)
O(2W)	H(2WA)	O(1)	2_645	1.00(5)	2.01(5)	2.938(3)	153(4)
O(2W)	H(2WB)	S(1)		0.95(5)	2.73(5)	3.538(2)	144(3)
O(2W)	H(2WB)	O(8A)		0.95(5)	2.01(5)	2.900(3)	156(4)
O(2W)	H(2WB)	O(7B)		0.95(5)	2.03(5)	2.856(7)	144(4)

Where 2_545 denotes symmetry operation -x, -y-1,-z and 2_645 corresponds to 1-x,-y-1,-z.

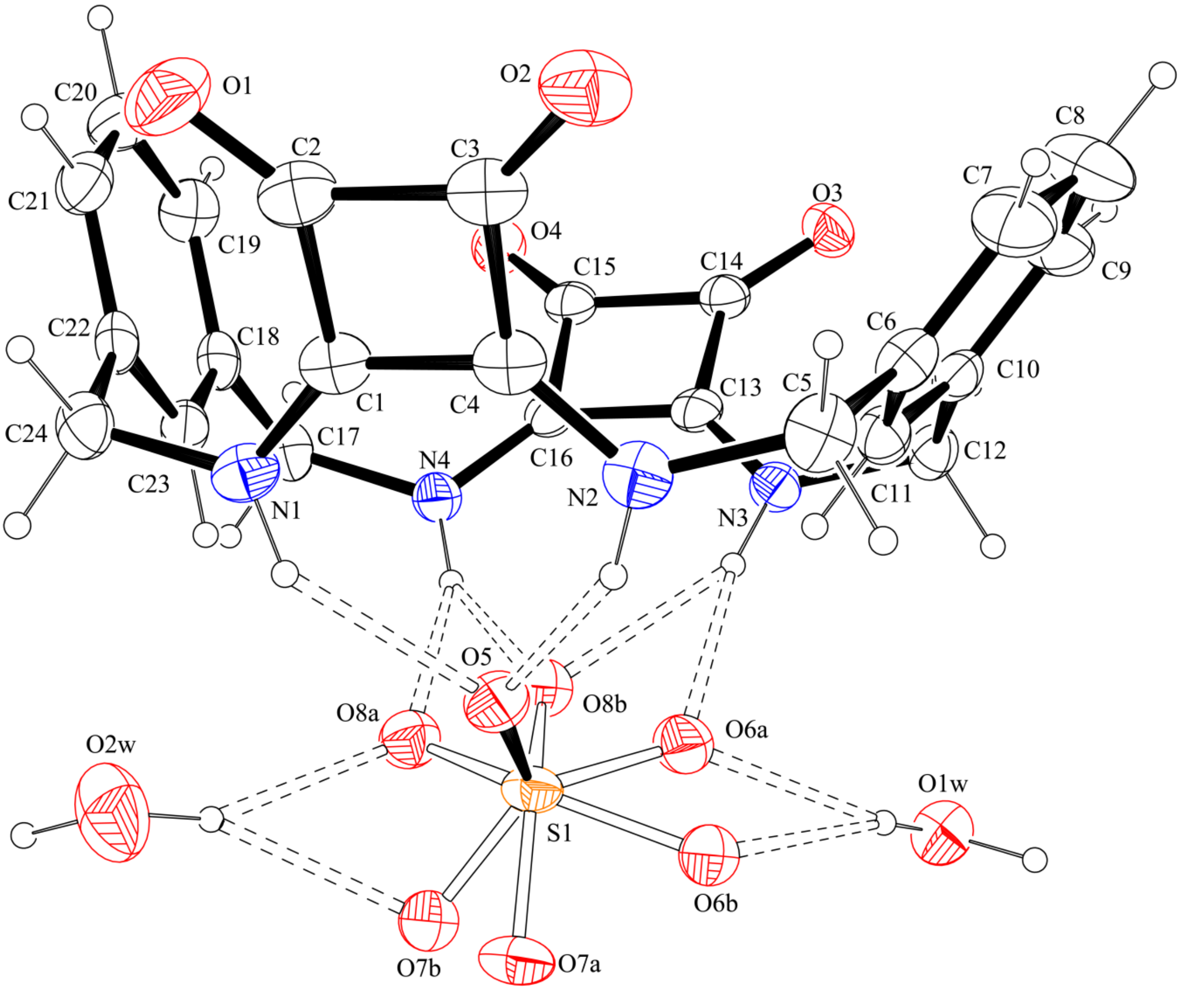

Figure S34. ORTEP depiction for **1-SO_4^{2-}** with 50% ellipsoids. The sulfate is disordered over two orientations about the S1-O5 axis. Occupancies were refined and then fixed at the second decimal place to be 0.75 (labels with ‘a’ as the suffix) and 0.25 (labels with ‘b’ as the suffix). Isotropic displacement parameters were used for the minor occupancy sites. The disorder reflects two binding modes, with the dominant mode involving three sulfate atoms and the minor mode just two oxygen atoms.

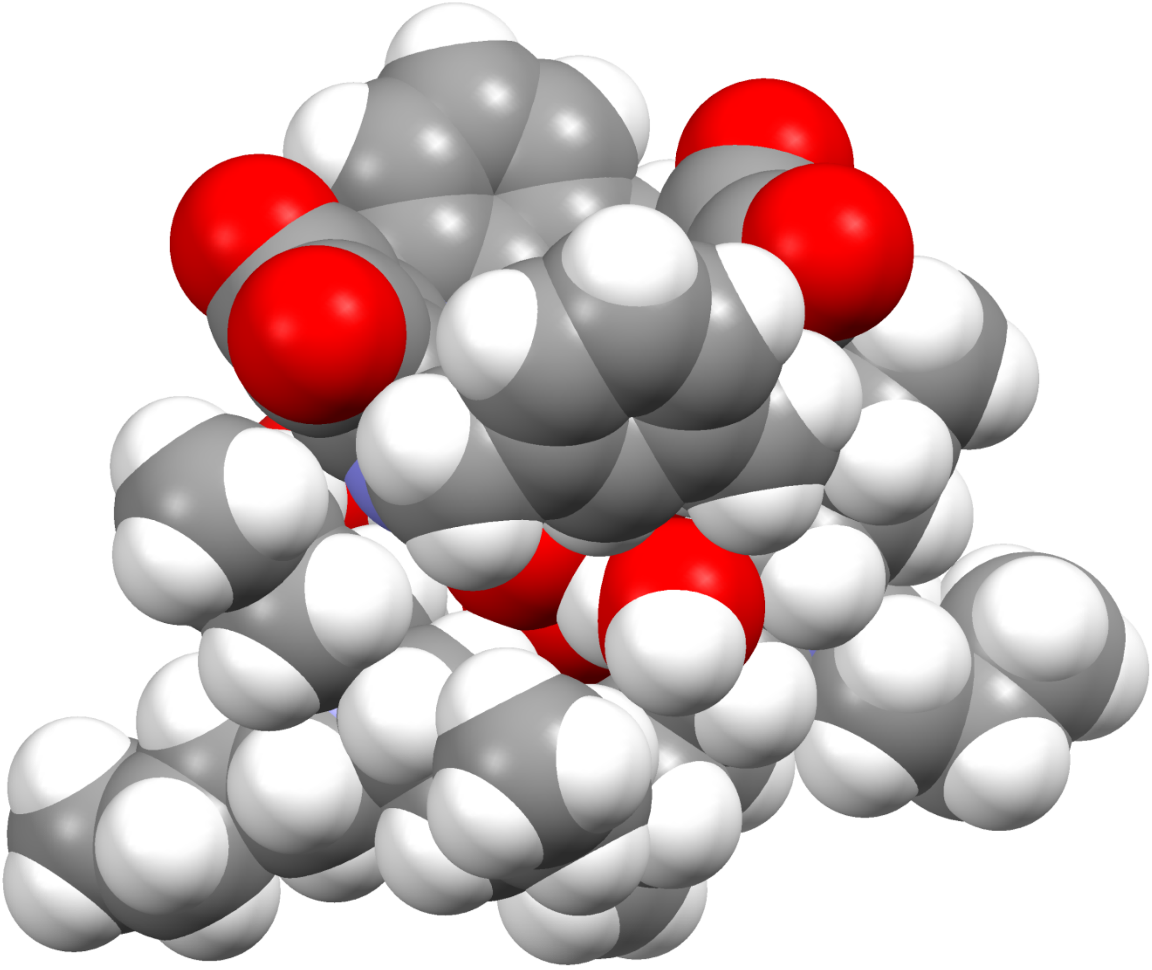

Figure S35. Space filling depiction of **1-SO_4^{2}** showing it 'nestled' against the two tetrabutylammonium cations present in the asymmetric unit. The bound sulfate anion is obscured by the cations and a water molecule.

Compound 5

Data were collected from a colourless prismatic crystal of **5** with a SuperNova Dual diffractometer equipped with an Atlas detector and employing mirror monochromated Cu (Kα) radiation from a micro-source. Cell constants were obtained from a least squares refinement against 5,084 reflections located between 8 and 150° 2θ. Data were collected at 150(1) Kelvin with ω scans to 153° 2θ. The data processing was undertaken with CrysAlis Pro.[7]

The data indicated a two component twinned or split crystal, with a 2.6° rotation about a non-integer axis and a minor component fraction of 0.33. The structure was solved in the space group $P\ \bar{1}$(#2) by direct methods using SHELXT.[1] The squaramide macrocycle molecule is centred about an inversion centre and the asymmetric unit accordingly contains half of the molecule. The pendant alkyl ether residue was modelled as being disordered over two orientations, with site occupancies refined and then fixed at 0.7 and 0.3. In general the non-hydrogen atom sites were modelled with anisotropic displacement parameters, with the minor occupancy sites modelled with isotropic displacement parameters. The amine hydrogen sites were located in difference maps and modelled with isotropic displacement parameters. A riding atom model was used for the remainder of the hydrogen sites. An ORTEP depiction of the molecule with 50% displacement ellipsoids is provided in Figure S36, where the superscript indicates the inversion operation; 2-x, 1-y, -z.

The length and width of the cavity is effectively determined by the separation between the four 'corner' carbon atoms that link the phenyl groups and squaramide groups and in **5** these distances are 6.125(4) across the squaramide group (distance between C5 and C12^{i}) and 5.031(4) Å across the phenyl group (distance between C5 and C12). The cavity shape is essentially rectangular, with an internal corner angle of 89.9°.

The least squares planes defined by the carbon atoms of the two squaramide groups are separated by 4.891(4)Å and the centroid to centroid distance is 5.179(3)Å. The relative lateral displacement or offset of the squaramide groups is approximately 1.70Å.

The least squares planes defined by the carbon atoms of the two phenyl groups are separated by 5.067(8)Å and the centroid to centroid distance is 8.308(4)Å. The relative lateral displacement of the squaramide groups is 6.58Å.

The cavity plane is effectively defined by the four 'corner' carbon atoms that link the phenyl groups to the squaramide moieties (C5, C12, C5^{i}, C12^{i}) and the phenyl carbon located on the cavity edge (C11 and C11^{i}). The largest deviation of a defining atom from the least squares plane defined by these atom sites is 0.008(3)Å.

The least squares planes of the squaramide and phenyl groups are respectively inclined at an angle of 77.9(1)° and 53.23(7)° with respect to the least squares plane of the cavity. The amide torsion angles (C7-C6-C5-N2 and C9-C10-C12-N1^{i}) are 133.9(2) and -108.2(3)°. The nitrogen atoms are displaced 0.281(3)Å (N1) and 0.298(3) Å (N2) from the cavity plane.

As Figures S37 and S38 depict, the least squares planes of the inversion related squaramide groups are parallel and hydrogen bonds link adjacent macrocycles to form a pseudo-rectangular channel parallel to the *a* axis and centred on (0,1/2,0).

Key hydrogen bond geometry:

Donor	Hydrogen	Acceptor		D-H(Å)	H-A(Å)	D-A(Å)	DHA Angle(°)
N(1)	H(1N)	O(1)	1_455	0.87(3)	1.94(3)	2.762(3)	157(3)
N(2)	H(2N)	O(2)	1_455	1.02(3)	1.87(3)	2.786(3)	147(2)

Where 1_455 indicates the symmetry operation x-1, y, z

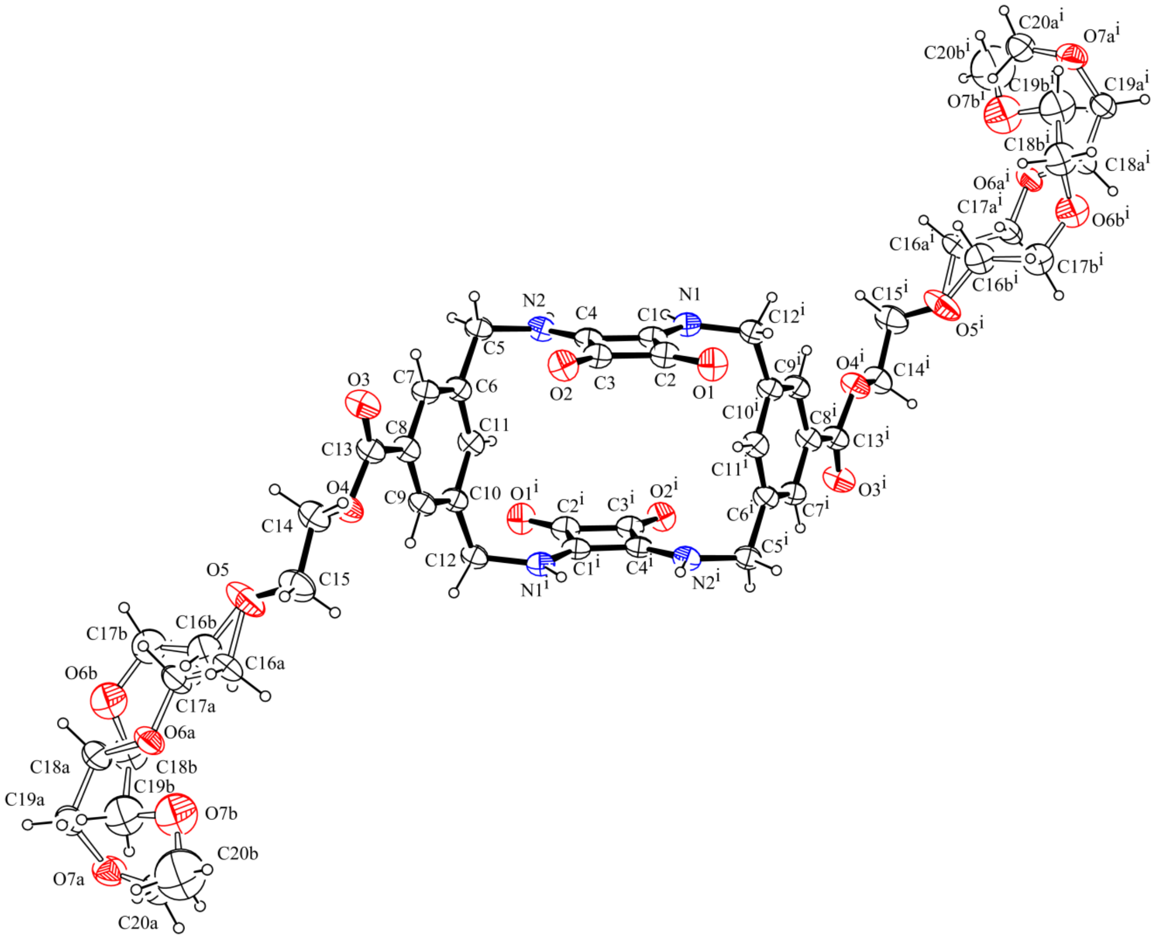

Figure S36. ORTEP depiction for **5** with 50% ellipsoids; the superscript indicates the inversion operation; 2-x, 1-y, -z. The pendant alkyl ether residue was modelled as being disordered over two orientations, with site occupancies refined and then fixed at 0.7 and 0.3. The minor occupancy sites modelled with isotropic displacement parameters.

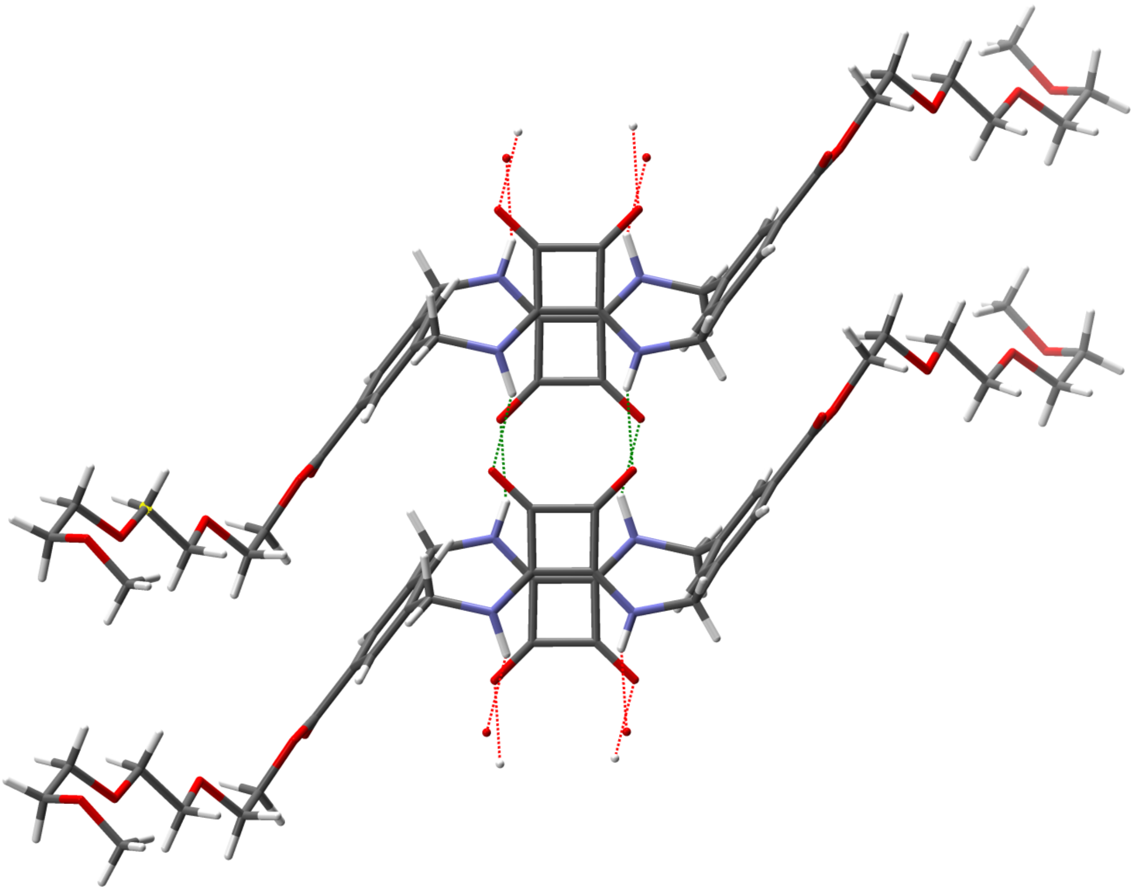

Figure S37. Hydrogen bonds link adjacent molecules to form an approximately rectangular channel parallel to the *a* axis and centred on (0,1/2,0) (see Figure S7). The centroid to centroid distance between the squaramide moieties is 5.179(3) Å and the two squaramides have a relative 'slippage' of 1.70 Å.

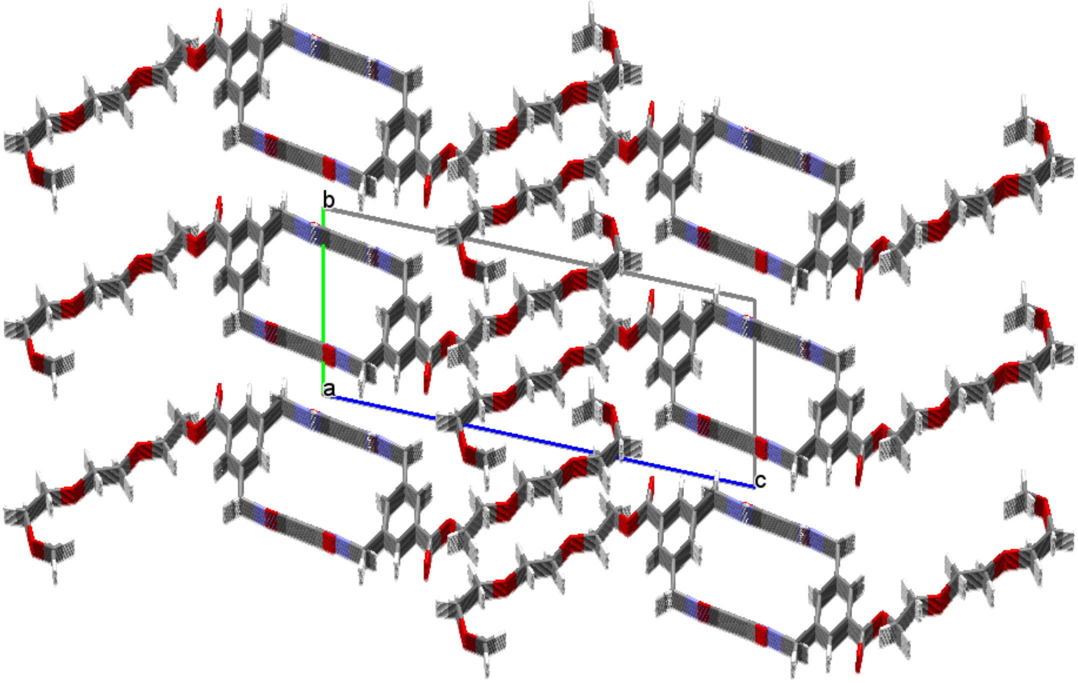

Figure S38. View down the *a* axis of the unit cell showing the pseudo-rectangular channels arising from hydrogen bonds between adjacent squaramides (see Figure S6). The channels are centred on (0,1/2,0) and are parallel to the *a* axis. The least squares planes of the squaramides. are separated by 4.891(4) Å

Table S1. Summary of crystallographic data

	**1**	**3**	**1-SO$_4^{2-}$**	**5**
Model Formula	$C_{28}H_{32}N_4O_6S_2$	$C_{28}H_{32}N_4O_6S$	$C_{56}H_{96}N_6O_{10}S$	$C_{40}H_{48}N_4O_{14}$
Molecular Weight	584.69	584.69	1045.44	808.82
Crystal System, Z	triclinic, 1	triclinic, 1	monoclinic,4	triclinic, 1
Space Group	$P\ \bar{1}$(#2)	$P\ \bar{1}$(#2)	P2$_1$/c(#14)	$P\ \bar{1}$(#2)
a	8.4474(5) Å	8.4511(2) Å	20.3065(19) Å	6.0434(3) Å
b	8.6414(4) Å	8.5911(2) Å	12.0238(10) Å	8.9035(5) Å
c	10.5047(6) Å	10.8922(2) Å	25.441(2) Å	20.1417(10) Å
α	101.755(4)°	73.717(2)°	90	101.174(5)°
β	103.383(5)°	84.157(2)°	110.374(5)°	91.268(4)°
γ	98.623(4)°	69.545(2)°	90	107.342(5)°
V	714.54(7) Å^3	711.23(3) Å^3	5823.1(9) Å^3	1011.20(10) Å^3
Size (mm)	0.168x0.139x0.063	0.150x0.110x0.070	0.12x0.10x0.08	0.216x0.030x0.020
Crystal Colour	pale yellow	colourless	colourless	colourless
Crystal Habit	prismatic	prismatic	prismatic	rectangular
Temperature	150(1) Kelvin	150(1) Kelvin	100(1) Kelvin	150(1) Kelvin
λ(Cu Kα)	1.5418 Å	1.5418 Å	0.71073 Å	1.5418 Å
μ(Cu Kα)	2.098 mm^{-1}	2.108 mm^{-1}	0.115 mm^{-1}	0.848 mm^{-1}
$T_{min,max}$	0.917, 1.00	0.857, 1.00	0.907, 1.000	0.813, 1.00
$2\theta_{max}$	152.29°	152.55°	50.70°	152.58°
hkl range	-10 10,-8 10,-13 10	-9 10,-10 10,-13 13	-24 24,-14 14,-30 30	-7 7,-11 11,-25 25
N	6264	26314	129874	9488
N_{ind}	2933(R_{merge} 0.0170)	2962(R_{merge} 0.021)	10639(R_{merge} 0.0619)	non-merohedral twin
N_{obs}	2716(I > 2σ(I))	2896(I > 2σ(I))	8458(I > 2σ(I))	4992(I > 2σ(I))
N_{var}	191	189	696	301
Residuals* $R1(F)$, $wR2(F^2)$	0.0328, 0.0902	0.0358, 0.0881	0.0482, 0.1182	0.0509, 0.1096
GoF(all)	1.323	1.310	1.184	1.176
Residual Density (e$^-$Å^{-3})	-0.292, 0.375	-0.426, 0.300	-0.414, 0.517	-0.324, 0.356
a, b*	0.04, 0.1	0.03, 0.3	0.035, 0.4	0.3,0

*$R1 = \Sigma||F_o| - |F_c||/\Sigma|F_o|$ for $F_o > 2\sigma(F_o)$; $wR2 = (\Sigma w(F_o^2 - F_c^2)^2/\Sigma(wF_c^2)^2)^{1/2}$ all reflections with weight w:

$w=1/[\sigma^2(F_o^2)+(aP)^2+ bP]$ where $P=(F_o^2+2F_c^2)/3$

Table S2. Summary of squaramide (SqA) macrocycle cavity geometry

	**1**	**3**	**1+SO_4^{2-}**	**5**
Cavity dimension 1[a] (Å)	6.062(2)	4.732(3)	6.070 (3)<br>6.041(2)	6.125(4)
Cavity dimension 2[b] (Å)	5.075 (2)	5.837(2)	5.050(4)<br>4.998(3)	5.031(4)
Cavity corner angles (°)	87.1, 92.9	82.6,97.4	89.1,90.68<br>9.9,90.6	90.1,89.9
SqA plane to SqA plane angle (°)	0	0	73.5(1)	0
SqA plane separation (Å)	4.832(6)	6.288(6)	-	4.891(4)
SqA to SqA centroid distance (Å)	7.233(2)	8.590(2	7.152(3)	5.179(3)
SqA to SqA lateral offset (Å)	5.38	5.85	-	1.70
Phenyl plane to phenyl plane angle (°)	0	0	50.78(8)	0
Phenyl plane separation (Å)	3.672(5)	4.635(2)	-	5.067(8)
Phenyl to phenyl centroid distance (Å)	8.463(2)	4.667(2)	7.323(2)	8.308(4)
Phenyl to phenyl lateral offset (Å)	7.62	0.55	-	6.58
SqA plane to cavity plane(s) angle(s) (°)	60.65(6)	79.66(6)	57.39(9)<br>49.12(8)	77.9(1)
Phenyl plane to cavity plane(s) angle(s) (°)	37.72(4)	88.30(4)	52.68(6)<br>76.55	53.23(7)
Nitrogen distance from cavity plane	0.340(1)<br>0.286(1)	1.266(2)<br>0.1434(2)	0.393(2)<br>0.379(2)<br>0.334(2)<br>0.459(2)	0.281(3)<br>0.298(3)
Amide torsion angles	125.3(1)<br>-152.5 (1)	81.8(2)<br>164.9(2)	136.5(3)<br>-136.0(2)<br>112,4(2)<br>-111.5 (2)	133.9(2)<br>-108.2(3)

[a]Distance between cavity 'corner' carbon atoms across a squaramide group. [b]Distance between cavity 'corner' carbon atoms across a phenyl group.

X-ray crystallography References

1. (a) Sheldrick, G.M.; SHELX97 Programs for Crystal Structure Analysis. University of Göttingen. Institüt für Anorganische Chemie der Universität, Tammanstrasse 4, D-3400 Göttingen, Germany, 1998. (b) G. M. Sheldrick, Acta Crystallogr., Sect. A: Found. Crystallogr., 2008, 64, 112-122. (c) Sheldrick, G. M. (2015), Acta Cryst. C71, 3-8.
2. WinGX, Farrugia, L. J. (1999) J. Appl. Cryst., 32, 837-838.
3. ShelXle: a Qt graphical user interface for SHELXL; C. B. Hübschle, G. M. Sheldrick and B. Dittrich. J. Appl. Cryst. (2011). 44, 1281-1284.
4. (a) ORTEP for Windows. L. J. Farrugia, J. Appl. Crystallogr., 1997, 30, 565. (b) ORTEP-III. M. N. Burnett and C. K. Johnson, ORTEP-III Report ORNL-6895. Oak Ridge National Laboratory, Tennessee, USA, 1996.
5. (a) Mercury CSD 2.0 - New Features for the Visualization and Investigation of Crystal Structures . C. F. Macrae, I. J. Bruno, J. A. Chisholm, P. R. Edgington, P. McCabe, E. Pidcock, L. Rodriguez-Monge, R. Taylor, J. van de Streek and P. A. Wood, J. Appl. Cryst., 41, 466-470, 2008. (b) Mercury: visualization and analysis of crystal structures. C. F. Macrae, P. R. Edgington, P. McCabe, E. Pidcock, G. P. Shields, R. Taylor, M. Towler and J. van de Streek, J. Appl. Cryst., 39, 453-457, 2006
6. Hall, S.R., du Boulay, D.J. & Olthof-Hazekamp, R. (1999) Eds. Xtal3.6 System . University of Western Australia.
7. CrysAlisPro Version 1.171.37.31d. Agilent Technologies, 2013.
8. A. Altomare, M. C. Burla, M. Camalli, G. L. Cascarano, C. Giacovazzo, A. Guagliardi, A. G. G. Moliterni, G. Polidori and R. Spagna, J. Appl. Crystallogr., 1999, 32, 115-119.
7. Bruker (2012); APEX, SAINT and XPREP. Area detector control and data integration and reduction software. Bruker Analytical X-ray Instruments Inc., Madison, Wisconsin, USA.
9. (a) Sheldrick, G.M.; SADABS. Empirical absorption correction program for area detector data. University of Göttingen, Germany, 1996. (b) Blessing, R.H.; Acta Cryst. (1995) A51 33 - 38.
